# Supplementary figures and images for: Staphylococcus epidermidis uses the SrrAB regulatory system to modulate oxidative stress and intracellular survival in mouse macrophage cell line Ana-1
Source: mSystems. 2025 Apr 22;10(5):e01737-24. doi: 10.1128/msystems.01737-24 (PMC12090800; doi:10.1128/msystems.01737-24)

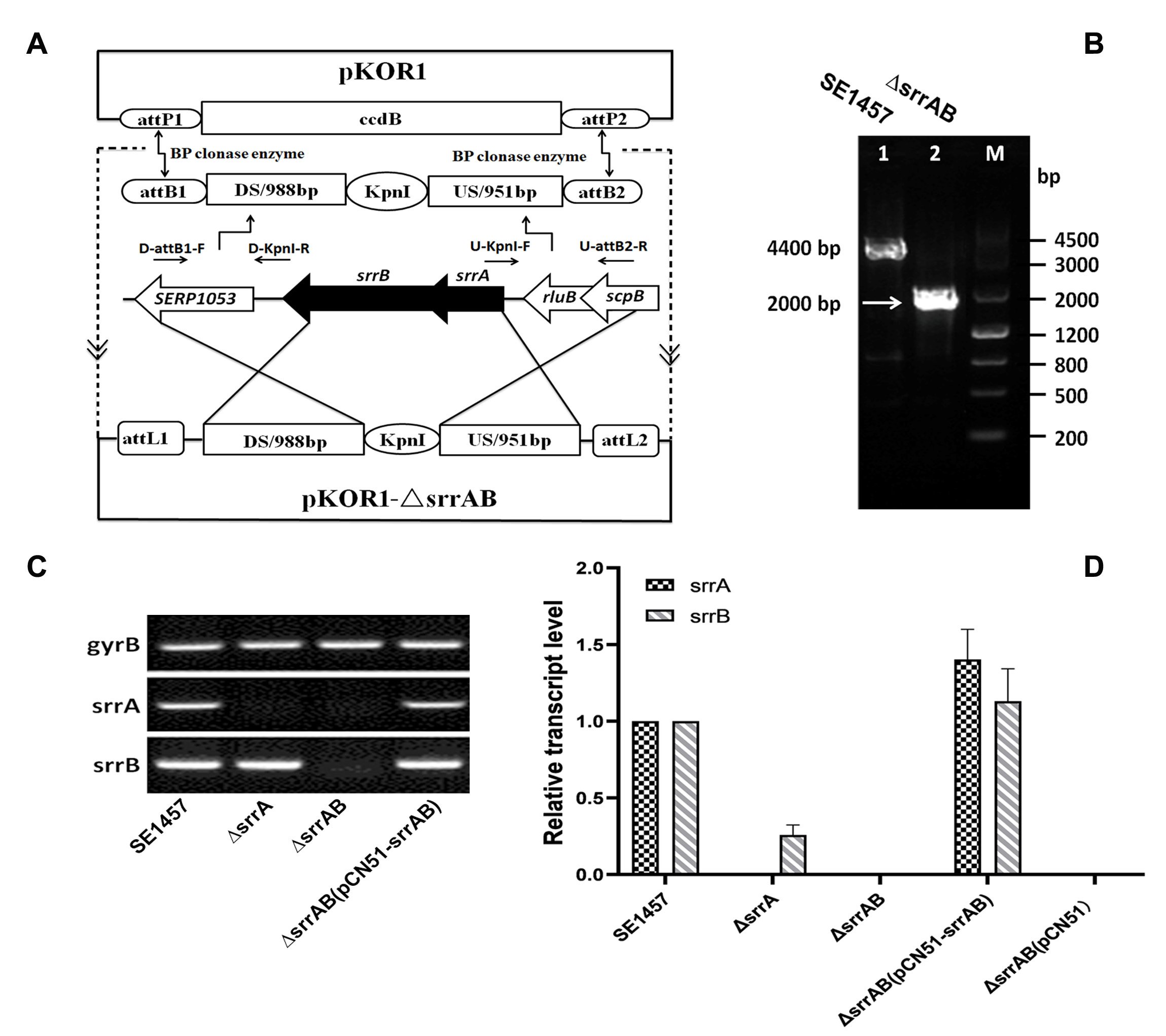

Supplement: Fig. S1 — Construction of srrAB deletion in SE1457 by allelic replacement. [file msystems.01737-24-s0001.tif]

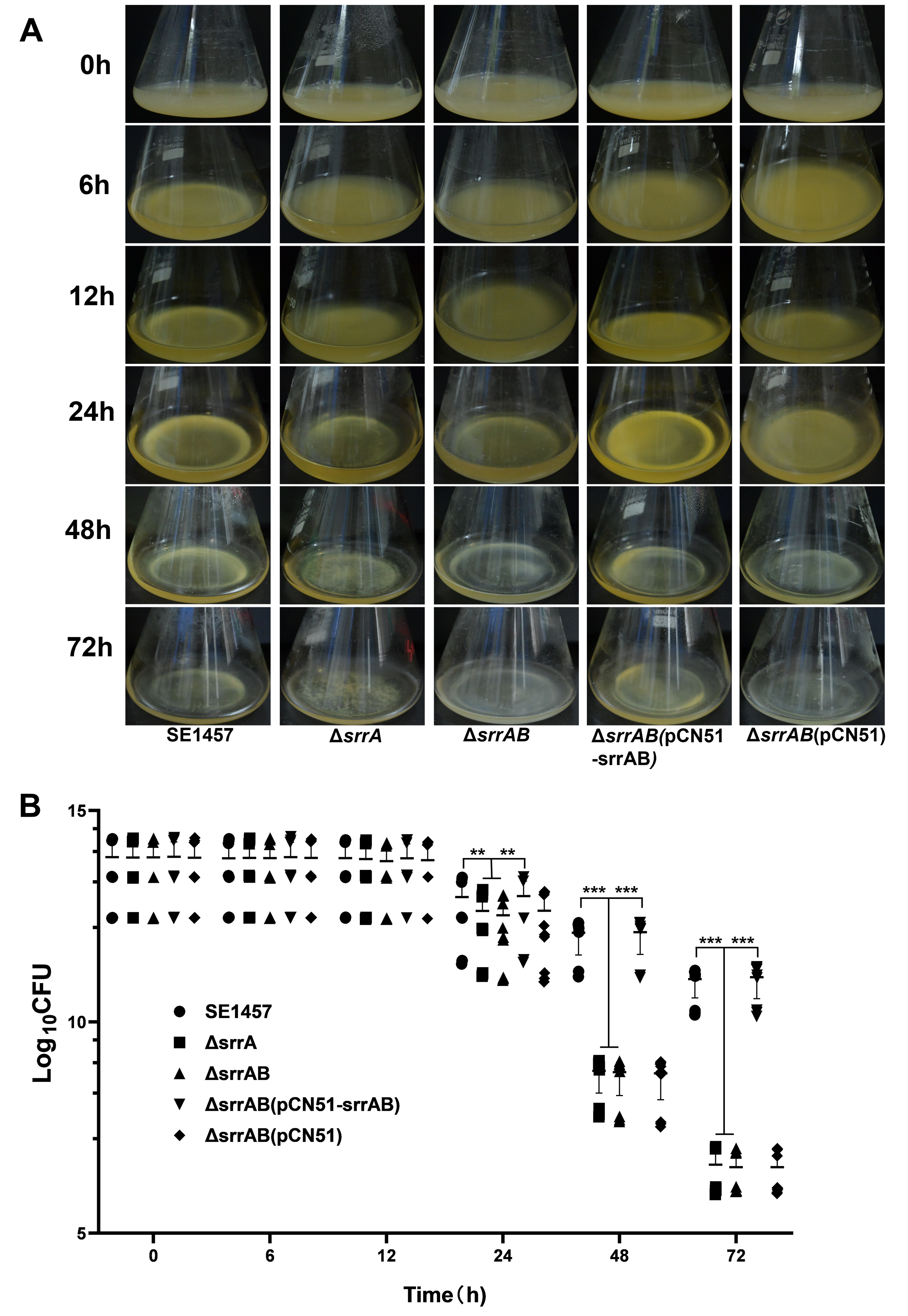

Supplement: Fig. S2 — Viability detection by CFU counting. [file msystems.01737-24-s0002.tif]
