# Supplementary material for: Staphylococcus epidermidis uses the SrrAB regulatory system to modulate oxidative stress and intracellular survival in mouse macrophage cell line Ana-1
Source: mSystems. 2025 Apr 22;10(5):e01737-24. doi: 10.1128/msystems.01737-24 (PMC12090800; doi:10.1128/msystems.01737-24)
Supplement: Legends — for supplemental figures and tables. [file msystems.01737-24-s0003.docx]

**Supplemental materials**

**Fig S1 Construction of *srrAB* deletion in SE1457 by allelic replacement.** (A) The downstream (988 bp) and upstream (951 bp) flanking sequences of *srrAB* were amplified from SE1457 genomic DNA, and ligated into the fragment of DS-US using the enzyme *EcoR*I, then amplified with primers containing attB1 and attB2 sites at 5’ end. The fragment of attB1-DS-US-attB2 was inserted into pKOR1 using the BP Clonase enzyme, yielding the recombinant plasmid pKOR1-∆*srrAB*, which was then transformed into SE1457 for allelic replacement. (B) Confirmation of the *srrAB* deletion mutant by PCR using the primer pairs srrA-DS-F/srrA-US-R. Lane 1, genomic DNA of SE1457 parent strain was designated as a template; lane 2, genomic DNA of the *srrAB* deletion mutant as the template; M, DNA marker III. Due to approximately 2400 bp deletion of *srrAB* genes, the PCR-fragment amplified from the *srrAB* deletion mutant was smaller than that of SE1457. The *srrAB* deletion mutant was also confirmed by reverse transcription (RT)-PCR (C) and Real-time quantitative (qRT)-PCR (D). The *srrA* deletion mutant (*∆srrA*) derived from SE1457 was designated as a control, the levels of *srrA/srrB* transcripts were normalized against the level of *gyrB* (housekeeping gene) transcript. Data represented the mean ± SD from three independent experiments.

**Fig S2 Viability detection by CFU counting.** SE1457 *srrAB* isogenic mutants were diluted (1:200) in fresh TSB medium, and incubated at 37℃ with shaking. Overnight cultures were removed and kept at room temperature for 72 h (A). At each time point (0, 6, 12, 24, 48, 72h), bacterial suspension was washed twice with normal saline, and serially diluted (10-fold). Each aliquot of 100 μL was spotted onto a TSA plate for CFU counting (3 petri dishes per dilution) (B). Data represented the mean ± SD from four independent experiments. ***, *P*＜0.001 (Δ*srrAB* mutant versus SE1457, Δ*srrAB*(pCN51*-srrAB*) versus Δ*srrAB* mutant）

**Table S1 Effect of *srrAB* deletion on the intracellular ROS production in *S. epidermidis*-infected Ana-1 cells**

The mouse Ana-1 cells co-incubated with *S. epidermidis* strains were stained by DCFH-DA for 20 min in dark and determined by flow cytomyter. Of 50000 cells counted, the number of ROS-positive cells was shown. The experiments were repeated at least 3 times, and the data represent as means ± SD. **, *P* ＜0.01 [ΔsrrA mutant versus SE1457, Δ*srrAB* mutant versus SE1457, Δ*srrAB*(pCN51-*srrAB*) versus Δ*srrAB*(pCN51).
