# Supplementary material for: Staphylococcus epidermidis uses the SrrAB regulatory system to modulate oxidative stress and intracellular survival in mouse macrophage cell line Ana-1
Source: mSystems. 2025 Apr 22;10(5):e01737-24. doi: 10.1128/msystems.01737-24 (PMC12090800; doi:10.1128/msystems.01737-24)
Supplement: Table S1 — Effect of srrAB deletion on the intracellular ROS production in S. epidermidis-infected Ana-1 cells [file msystems.01737-24-s0004.docx]

**Supplemental materials**

**Table S1 Effect of *srrAB* deletion on the intracellular ROS production in *S. epidermidis*-infected Ana-1 cells**

| Strains | Total Count | DCF Count |
| --- | --- | --- |
| SE1457 | 47685.67±195.45 | 4998.67±339.14 |
| Δ*srrA* | 47696.33±101.64 | 8988.33±120.5^**^ |
| Δ*srrAB* | 48016.67±46.23 | 10707.67±186.69^**^ |
| Δ*srrAB*(pCN51-*srrAB*) | 47689.33±239.79 | 4489.67±123.29 |
| Δ*srrAB*(pCN51) | 46370±190.44 | 8937.33±334.45^**^ |
| Rousp | 46857.33±17.56 | 4941±116.04 |
