# Supplementary material for: Staphylococcus epidermidis uses the SrrAB regulatory system to modulate oxidative stress and intracellular survival in mouse macrophage cell line Ana-1
Source: mSystems. 2025 Apr 22;10(5):e01737-24. doi: 10.1128/msystems.01737-24 (PMC12090800; doi:10.1128/msystems.01737-24)
Supplement: Table S2 — RNA Seq analysis of the srrAB deletion mutant in S. epidermidis. [file msystems.01737-24-s0005.pdf]

Table S2 RNA Seq analysis of the *srrAB* deletion mutant in *S. epidermidis*

gene id	gene name	description	locus	log2FC	Pvalue	Qvalue	updown
SE2169	SERP2169	Putative pyruvate, phosphate dikinase regulatory protei	CP000029.1:2198607-2199425	-5.92375	2.6E-133	5.5E-130	DOWN
SE2170	ppdK	pyruvate, phosphate dikinase	CP000029.1:2199427-2202054	-4.52617	1.85E-97	1.95E-94	DOWN
SE2352	arcC	Carbamate kinase 1	CP000029.1:2397500-2398432	-4.4533	2.13E-91	1.5E-88	DOWN
SE2351	arcB-2	Ornithine carbamoyltransferase	CP000029.1:2396476-2397477	-4.29468	2.11E-83	1.11E-80	DOWN
SE2379	SERP2379	L-2,3-butanediol dehydrogenase	CP000029.1:2425762-2426535	-3.97566	2.55E-81	1.08E-78	DOWN
SE2312	mqo-3	malate:quinone-oxidoreductase	CP000029.1:2350001-2351497	5.665563	1.87E-79	6.58E-77	UP
SE1054	srrB	sensor histidine kinase ResE	CP000029.1:1099964-1101733	inf (-)	9.27E-76	2.79E-73	DOWN
SE2365	pflA	pyruvate formate-lyase-activating enzyme	CP000029.1:2411378-2412133	-3.48339	4.5E-70	1.19E-67	DOWN
SE1055	srrA	DNA-binding response regulator ResD	CP000029.1:1101714-1102439	-5.33329	8.94E-67	2.1E-64	DOWN
SE0364	saeS	sensor histidine kinase	CP000029.1:364454-365419	4.748559	2.25E-66	4.74E-64	UP
SE2321	SERP2321	-	CP000029.1:2359074-2359586	-3.46006	7.56E-66	1.45E-63	DOWN
SE0365	saeR	Response regulator SaeR	CP000029.1:365509-366198	4.834276	9.81E-65	1.73E-62	UP
SE2114	SERP2114	PTS system, glucose-specific IIABC component	CP000029.1:2137627-2139654	4.53623	1.14E-64	1.85E-62	UP
SE2353	SERP2353	Uncharacterized protein	CP000029.1:2398597-2400162	-3.3767	9.27E-64	1.4E-61	DOWN
SE2366	pflB	Formate acetyltransferase	CP000029.1:2412155-2414401	-3.14027	2.45E-61	3.45E-59	DOWN
SE0095	SERP0095	cystathionine beta-lyase	CP000029.1:80406-81551	6.790525	9.61E-61	1.27E-58	UP
SE0366	SERP0366	-	CP000029.1:366173-366646	5.175099	5.46E-60	6.78E-58	UP
SE0653	purQ	Phosphoribosylformylglycinamidine synthase subunit Pu	CP000029.1:646768-647439	4.724593	9.26E-56	1.09E-53	UP
SE2257	SERP2257	L-2,3-butanediol dehydrogenase	CP000029.1:2285934-2286722	-3.34372	3.29E-55	3.66E-53	DOWN
SE0654	purL	Phosphoribosylformylglycinamidine synthase subunit Pu	CP000029.1:647432-649621	4.285986	3.04E-54	3.21E-52	UP
SE2380	SERP2380	drug resistance transporter, EmrB/QacA subfamily, puta	CP000029.1:2426704-2428080	-3.05037	1.65E-53	1.66E-51	DOWN
SE0646	qoxB	Quinol oxidase subunit 2	CP000029.1:640787-641911	-2.83122	1.76E-53	1.69E-51	DOWN
SE0643	qoxD	Quinol oxidase subunit 4	CP000029.1:637917-638207	-2.7777	5E-52	4.59E-50	DOWN
SE0644	qoxC	Quinol oxidase subunit 3	CP000029.1:638204-638809	-2.68318	9.13E-50	7.75E-48	DOWN
SE0656	purM	Phosphoribosylformylglycinamidine cyclo-ligase	CP000029.1:651077-652108	3.996427	9.18E-50	7.75E-48	UP
SE0645	qoxA	Quinol oxidase subunit 1	CP000029.1:638799-640787	-2.66781	1.95E-49	1.58E-47	DOWN
SE1772	SERP1772	-	CP000029.1:1816431-1816691	-2.65317	5.43E-49	4.24E-47	DOWN
SE0651	purC	Phosphoribosylaminoimidazole-succinocarboxamide sy	CP000029.1:645802-646536	4.716609	3.64E-48	2.75E-46	UP
SE0655	purF	amidophosphoribosyltransferase	CP000029.1:649600-651084	3.888448	9.55E-48	6.95E-46	UP
SE0657	purN	phosphoribosylglycinamide formyltransferase	CP000029.1:652108-652674	4.110486	5.23E-47	3.68E-45	UP
SE2051	SERP2051	-	CP000029.1:2073388-2073579	-2.58182	6.02E-47	4.1E-45	DOWN
SE2390	sspB	-	CP000029.1:2440678-2441865	-2.53708	5.08E-46	3.35E-44	DOWN
SE0367	SERP0367	-	CP000029.1:366697-367134	4.840399	2.22E-45	1.42E-43	UP
SE2382	SERP2382	thiamin biosynthesis lipoprotein ApbE	CP000029.1:2431209-2432126	-3.66499	2.53E-45	1.57E-43	DOWN
SE2381	SERP2381	Probable NADH-dependent flavin oxidoreductase YqiG	CP000029.1:2428109-2431126	-2.75973	2.33E-44	1.4E-42	DOWN
SE0650	purK	N5-carboxyaminoimidazole ribonucleotide synthase	CP000029.1:644674-645801	3.859307	6.79E-44	3.98E-42	UP
SE2134	SERP2134	aminotransferase, classes I and II	CP000029.1:2159602-2160762	-2.66356	3.44E-43	1.97E-41	DOWN
SE2364	SERP2364	Acetylornithine deacetylase	CP000029.1:2410022-2411239	-2.97478	7.02E-43	3.9E-41	DOWN
SE0658	purH	phosphoribosylaminoimidazolecarboxamide formyltrans	CP000029.1:652691-654169	3.65926	2.02E-42	1.09E-40	UP
SE1250	gapA-2	Glyceraldehyde-3-phosphate dehydrogenase 2	CP000029.1:1287163-1288188	-2.38778	3.56E-42	1.88E-40	DOWN
SE1273	SERP1273	universal stress protein family	CP000029.1:1318052-1318552	-2.35298	8.13E-42	4.18E-40	DOWN
SE0765	uraA	uracil permease	CP000029.1:762940-764232	3.917248	3.24E-41	1.63E-39	UP
SE0063	SERP0063	sodium:dicarboxylate symporter family protein	CP000029.1:50679-52067	3.750518	1.77E-40	8.7E-39	UP
SE1295	fhs	formate--tetrahydrofolate ligase	CP000029.1:1341346-1343013	3.576431	5.72E-40	2.75E-38	UP
SE0659	purD	phosphoribosylamine--glycine ligase	CP000029.1:654195-655436	3.497453	1.09E-38	5.11E-37	UP
SE2250	arcA	Arginine deiminase	CP000029.1:2277613-2278848	-2.27768	1.26E-38	5.8E-37	DOWN
SE0257	SERP0257	alcohol dehydrogenase, zinc-containing	CP000029.1:265167-266189	-2.22642	2.57E-38	1.15E-36	DOWN
SE2183	nrdD	Escherichia coli K-12	*nrdD*	-2.47799	3E-36	1.32E-34	DOWN
SE0324	scdA	Iron-sulfur cluster repair protein ScdA	CP000029.1:328812-329486	-2.34166	2.09E-35	9.01E-34	DOWN
SE2261	manA-2	Mannose-6-phosphate isomerase	CP000029.1:2291376-2292326	-2.07136	1.3E-34	5.47E-33	DOWN
SE2249	arcB-1	Ornithine carbamoyltransferase	CP000029.1:2276501-2277508	-2.11172	1.71E-33	7.08E-32	DOWN
SE2324	SERP2324	dihydrolipoamide acetyltransferase	CP000029.1:2360269-2361546	-1.98757	2.41E-33	9.77E-32	DOWN
SE0258	SERP0258	-	CP000029.1:266272-266664	-2.00664	2.55E-33	9.96E-32	DOWN
SE1734	pyrG	CTP synthase	CP000029.1:1772905-1774512	3.442059	2.55E-33	9.96E-32	UP
SE0519	dltB	dltB protein	CP000029.1:504627-505841	3.282928	1.67E-32	6.42E-31	UP
SE0325	SERP0325	Uncharacterized protein	CP000029.1:329658-330803	-1.90358	2.87E-31	1.08E-29	DOWN
SE1761	SERP1761	-	CP000029.1:1798883-1799038	-2.46944	2.76E-30	1.02E-28	DOWN
SE2003	SERP2003	amino acid ABC transporter, ATP-binding protein	CP000029.1:2021139-2021870	3.31049	1.51E-29	5.48E-28	UP
SE0259	SERP0259	-	CP000029.1:266777-266887	-1.96163	2.04E-29	7.31E-28	DOWN
SE0521	dltD	dltD protein	CP000029.1:506092-507330	3.106373	2.65E-29	9.34E-28	UP
SE2325	SERP2325	TPP-dependent acetoin dehydrogenase E1 beta-subunit	CP000029.1:2361560-2362600	-1.76838	7.82E-29	2.71E-27	DOWN
SE0083	SERP0083	-	CP000029.1:69480-69548	-1.78329	1.69E-28	5.76E-27	DOWN
SE2004	SERP2004	Amino acid ABC transproter, permease protein, putative	CP000029.1:2021867-2022586	3.437832	4.57E-28	1.53E-26	UP
SE2391	sspC	-	CP000029.1:2441883-2442200	-1.83117	6.69E-28	2.21E-26	DOWN
SE2247	arcD	Arginine/ornithine antiporter, putative	CP000029.1:2274833-2276236	-1.8389	1.04E-26	3.38E-25	DOWN
SE2260	SERP2260	PTS system, fructose-specific IIABC component	CP000029.1:2289420-2291360	-1.65805	2.23E-26	7.13E-25	DOWN
SE1446	purB	Adenylosuccinate lyase	CP000029.1:1511231-1512526	2.917622	1.13E-25	3.55E-24	UP
SE0635	SERP0635	transcriptional regulator, MarR family	CP000029.1:626997-627416	-1.74211	2.44E-25	7.57E-24	DOWN
SE1445	SERP1445	conserved hypothetical protein	CP000029.1:1510804-1511106	3.286578	6.6E-25	2.02E-23	UP
SE2128	SERP2128	1-pyrroline-5-carboxylate dehydrogenase	CP000029.1:2152253-2153797	-1.56295	1.05E-24	3.17E-23	DOWN
SE0930	fmtC	virulence factor MprF	CP000029.1:945777-948299	2.963706	1.07E-24	3.18E-23	UP
SE1132	glyS	Glycine--tRNA ligase	CP000029.1:1170931-1172322	2.817321	1.95E-24	5.72E-23	UP
SE0097	SERP0097	ABC transporter, ATP-binding protein	CP000029.1:81798-82823	4.64132	8.21E-24	2.38E-22	UP

SE1758	SERP1758	-	CP000029.1:1794229-1795380	2.968458	9.09E-24	2.59E-22	UP
SE0962	SERP0962	ABC transporter, ATP-binding protein	CP000029.1:979345-980952	2.890057	1.81E-23	5.09E-22	UP
SE1428	SERP1428	-	CP000029.1:1489727-1490263	-1.63061	4.92E-23	1.37E-21	DOWN
SERP_Set	SERP_Setm	-	CP000029.1:457703-458061	-1.46578	5.96E-23	1.63E-21	DOWN
SE2182	nrdG	Anaerobic ribonucleoside-triphosphate reductase-activa	CP000029.1:2215128-2215664	-2.59969	6.33E-23	1.71E-21	DOWN
SE1489	hld	-	CP000029.1:1557987-1558064	-1.46146	6.75E-23	1.8E-21	DOWN
SE2545	serS	seryl-tRNA synthetase	CP000029.1:2603756-2605042	-1.4535	1.12E-22	2.95E-21	DOWN
SE0526	SERP0526	Uncharacterized protein	CP000029.1:510104-510463	-1.55835	3.07E-22	8.01E-21	DOWN
SE2080	SERP2080	Escherichia coli K-12	CP000029.1:2106578-2108017	-1.40681	7.92E-22	2.04E-20	DOWN
SE2326	SERP2326	TPP-dependent acetoin dehydrogenase E1 alpha-subuni	CP000029.1:2362671-2363624	-1.412	1.43E-21	3.63E-20	DOWN
SE0649	purE	phosphoribosylaminoimidazole carboxylase, catalytic su	CP000029.1:644205-644687	2.891325	1.99E-21	5E-20	UP
SE1189	SERP1189	HTH-type transcriptional regulator CymR	CP000029.1:1224793-1225215	-1.54002	4.02E-21	9.98E-20	DOWN
SE1676	SERP1676	S1 RNA binding domain protein	CP000029.1:1720367-1722517	2.76764	8.78E-21	2.16E-19	UP
SE2192	cysH	phosphoadenosine phosphosulfate reductase	CP000029.1:2226564-2227295	5.096511	1.33E-20	3.23E-19	UP
SE1688	SERP1688	DEAD-box ATP-dependent RNA helicase CshA	CP000029.1:1730758-1732287	3.031727	1.88E-20	4.5E-19	UP
SE1773	SERP1773	Heme-degrading monooxygenase	CP000029.1:1816844-1817158	-2.02066	2.68E-20	6.35E-19	DOWN
SE0652	purS	phosphoribosylformylglycinamidine synthase, PurS prot	CP000029.1:646506-646766	4.284392	3.72E-20	8.72E-19	UP
SE0098	SERP0098	ABC transporter, permease protein, putative	CP000029.1:82826-83485	3.490977	2.51E-19	5.82E-18	UP
SE2147	SERP2147	Hypothetical cytosolic protein	CP000029.1:2173685-2174401	3.265828	2.67E-19	6.13E-18	UP
SE1760	glmS	Glutamine--fructose-6-phosphate aminotransferase [isor	CP000029.1:1797037-1798842	-1.27156	3.11E-19	7.07E-18	DOWN
SE1994	SERP1994	Uncharacterized protein	CP000029.1:2012875-2014350	2.930249	3.42E-19	7.67E-18	UP
SE0907	SERP0907	conserved hypothetical protein	CP000029.1:917887-918912	2.662757	4.13E-19	9.18E-18	UP
SE0392	SERP0392	Di-or tripeptide:H+ symporter	CP000029.1:394195-395700	2.585518	1.31E-18	2.88E-17	UP
SE2103	dep	Gamma glutamyl transpeptidase	CP000029.1:2127505-2129109	2.917124	1.48E-18	3.21E-17	UP
SE0706	ctaB	protoheme IX farnesyltransferase	CP000029.1:703965-704876	-1.24333	1.64E-18	3.54E-17	DOWN
SE1294	SERP1294	penicillin-binding protein 1A	CP000029.1:1340351-1341256	-1.34544	2.23E-18	4.76E-17	DOWN
SE1790	lacE	PTS system lactose-specific EIICB component	CP000029.1:1836395-1838143	-1.27377	2.69E-18	5.68E-17	DOWN
SE0099	SERP0099	ABC transporter, substrate-binding protein, putative	CP000029.1:83529-84374	2.974723	2.93E-18	6.12E-17	UP
SE2327	SERP2327	dihydrolipoamide dehydrogenase	CP000029.1:2363666-2365018	-1.22168	4.34E-18	8.99E-17	DOWN
SE0518	dltA	D-alanine-activating enzyme/D-alanine-D-alanyl carrier	CP000029.1:503173-504630	2.531145	4.91E-18	9.96E-17	UP
SE2005	SERP2005	amino acid ABC transporter, amino acid-binding protei	CP000029.1:2022567-2023355	2.756788	4.9E-18	9.96E-17	UP
SE0475	SERP0475	-	CP000029.1:473564-473800	-1.19575	5.04E-18	1.01E-16	DOWN
SE2001	fmhA	-	CP000029.1:2019216-2020463	2.803281	6.02E-18	1.2E-16	UP
SE2148	SERP2148	conserved hypothetical protein	CP000029.1:2174678-2176009	2.550161	6.1E-18	1.2E-16	UP
SE0315	SERP0315	efflux ABC transporter, permease protein	CP000029.1:318088-319977	2.518453	7.46E-18	1.46E-16	UP
SE1681	SERP1681	Endoribonuclease MazF	CP000029.1:1725803-1726165	3.748219	1.15E-17	2.22E-16	UP
SE0721	pheS	Phenylalanine--tRNA ligase alpha subunit	CP000029.1:717590-718648	2.670324	1.2E-17	2.3E-16	UP
SE0539	kapB	kinase-associated protein B	CP000029.1:523118-523498	-1.52894	2.95E-17	5.6E-16	DOWN
SE1789	lacG	6-phospho-beta-galactosidase	CP000029.1:1834967-1836379	-1.20464	3.35E-17	6.32E-16	DOWN
SE0480	SERP0480	organic hydroperoxide resistance protein	CP000029.1:476297-476719	-1.13825	4.68E-17	8.74E-16	DOWN
SE2035	SERP2035	AGAP006727-PA	CP000029.1:2056056-2057411	-1.27194	6.92E-17	1.28E-15	DOWN
SE1895	SERP1895	conserved hypothetical protein	CP000029.1:1917426-1918115	-1.12572	1.07E-16	1.96E-15	DOWN
SE1050	SERP1050	conserved hypothetical protein	CP000029.1:1097109-1098071	-1.26611	2.41E-16	4.39E-15	DOWN
SE0561	SERP0561	Uncharacterized protein	CP000029.1:552009-552830	-1.51928	2.64E-16	4.73E-15	DOWN
SE1416	SERP1416	cytosolic long-chain acyl-CoA thioester hydrolase famil	CP000029.1:1480132-1480662	2.446552	2.63E-16	4.73E-15	UP
SE0663	SERP0663	Oleate hydratase	CP000029.1:658883-660658	-1.08584	2.75E-16	4.87E-15	DOWN
SE2536	purA	Adenylosuccinate synthetase	CP000029.1:2592839-2594122	2.429251	3.41E-16	5.99E-15	UP
SE1243	rpmI	ribosomal protein L35	CP000029.1:1278698-1278898	2.6373	4.64E-16	8.1E-15	UP
SE2550	recF	DNA replication and repair protein RecF	CP000029.1:2611754-2612869	-1.08012	7.51E-16	1.3E-14	DOWN
SE0887	SERP0887	Uncharacterized protein	CP000029.1:900121-900852	2.852001	8.55E-16	1.47E-14	UP
SE0888	SERP0888	sensor histidine kinase	CP000029.1:900852-901946	2.486401	8.75E-16	1.49E-14	UP
SE1771	SERP1771	Escherichia coli K-12	CP000029.1:1815745-1816254	-1.15379	1.23E-15	2.07E-14	DOWN
SE2173	SERP2173	conserved hypothetical protein	CP000029.1:2204338-2204784	-1.25393	1.56E-15	2.61E-14	DOWN
SE1272	ald	Alanine dehydrogenase 2	CP000029.1:1316789-1317904	-1.04231	1.73E-15	2.88E-14	DOWN
SE0390	bmrU	bmrU protein	CP000029.1:392617-393540	-1.15452	2.25E-15	3.71E-14	DOWN
SE0828	cdsA	Phosphatidate cytidylyltransferase	CP000029.1:831728-832510	2.448092	2.36E-15	3.86E-14	UP
SE0815	SERP0815	DNA processing protein DprA, putative	CP000029.1:818735-819607	-1.33683	2.39E-15	3.89E-14	DOWN
SE0897	hom	homoserine dehydrogenase	CP000029.1:907781-909061	-1.26298	2.49E-15	4.01E-14	DOWN
SE2330	SERP2330	ABC transporter, periplasmic substrate-binding protein	CP000029.1:2367078-2367989	-1.19835	2.92E-15	4.68E-14	DOWN
SE0310	SERP0310	Uncharacterized protein	CP000029.1:313927-314433	-1.19404	3.3E-15	5.23E-14	DOWN
SE1238	clpX	ATP-dependent Clp protease ATP-binding subunit ClpX	CP000029.1:1273310-1274572	2.315271	5.54E-15	8.73E-14	UP
SE1239	tig	trigger factor	CP000029.1:1274859-1276160	2.316113	6.72E-15	1.05E-13	UP
SE0617	SERP0617	-	CP000029.1:611678-611896	-1.08511	7.1E-15	1.1E-13	DOWN
SE2541	SERP2541	Homoserine O-acetyltransferase	CP000029.1:2599586-2600554	-1.32971	1.06E-14	1.64E-13	DOWN
SE0909	lexA	LexA repressor	CP000029.1:919266-919886	-1.0349	1.08E-14	1.65E-13	DOWN
SE1689	murF	UDP-N-acetylmuramoylalanyl-D-glutamyl-2,6-diaminop	CP000029.1:1732587-1733942	2.37132	1.09E-14	1.65E-13	UP
SE0620	SERP0620	fatty acid desaturase family protein	CP000029.1:614935-615276	-1.08676	1.15E-14	1.74E-13	DOWN
SE1269	SERP1269	universal stress protein family	CP000029.1:1314095-1314508	-0.95675	3.46E-14	5.17E-13	-
SE0483	SERP0483	Thioredoxin, putative	CP000029.1:478402-478725	-1.1976	3.72E-14	5.53E-13	DOWN
SE1973	SERP1973	general stress protein 26	CP000029.1:1992431-1992853	-0.95037	4.04E-14	5.96E-13	-
SE0139	rplY	ribosoma protein L25	CP000029.1:129394-130053	2.331026	5.08E-14	7.39E-13	UP
SE1020	pbp2	penicillin-binding protein	CP000029.1:1063849-1066080	2.2137	5.05E-14	7.39E-13	UP
SE2034	SERP2034	Uncharacterized protein	CP000029.1:2054061-2055740	2.264131	6.13E-14	8.86E-13	UP
SE1959	SERP1959	-	CP000029.1:1977478-1978125	-0.9427	6.7E-14	9.62E-13	-
SE0520	dltC	D-alanyl carrier protein	CP000029.1:505859-506095	3.34611	7.28E-14	1.04E-12	UP
SE1351	SERP1351	-	CP000029.1:1407309-1408217	2.313953	9.61E-14	1.36E-12	UP

SE0307	SERP0307	-	CP000029.1:310246-310746	-0.94473	9.76E-14	1.37E-12	-
SERP_SeS	SERP_SeSR	-	CP000029.1:101004-101277	-0.91826	1.29E-13	1.81E-12	-
SE1438	gatA	glutamyl-tRNA(Gln) amidotransferase, A subunit	CP000029.1:1500371-1501828	2.20282	1.73E-13	2.4E-12	UP
SE0576	SERP0576	Regulatory protein Spx	CP000029.1:570409-570804	-0.90433	1.86E-13	2.57E-12	-
SE0648	folD	Bifunctional protein FolD	CP000029.1:643140-644000	2.233593	2.81E-13	3.85E-12	UP
SE1786	SERP1786	zinc-containing alcohol dehydrogenase (ADH)	CP000029.1:1831590-1832594	-0.89723	2.85E-13	3.88E-12	-
SE2375	SERP2375	-	CP000029.1:2423457-2424296	-3.69153	3.16E-13	4.27E-12	DOWN
SE0578	SERP0578	Adapter protein MecA 1	CP000029.1:572719-573441	-0.88619	4.64E-13	6.24E-12	-
SE1747	deoD	purine nucleoside phosphorylase	CP000029.1:1785492-1786202	2.19058	5.04E-13	6.73E-12	UP
SE1793	lacC	Tagatose-6-phosphate kinase	CP000029.1:1839464-1840396	-1.14691	7.58E-13	1.01E-11	DOWN
SE0582	SERP0582	protozoan/cyanobacterial globin family protein	CP000029.1:577659-578102	-0.92454	8.29E-13	1.09E-11	-
SE0886	SERP0886	ABC transporter, ATP-binding protein	CP000029.1:899249-900124	3.720547	1.14E-12	1.49E-11	UP
SE1986	narH	Nitrate reductase, beta subunit	CP000029.1:2002415-2003968	-0.86207	1.17E-12	1.52E-11	-
SE1038	SERP1038	Heptaprenyl diphosphate synthase component 2	CP000029.1:1083802-1084761	2.170952	1.29E-12	1.67E-11	UP
SE2082	SERP2082	Uncharacterized protein	CP000029.1:2109202-2109483	-0.84951	1.34E-12	1.72E-11	-
SE1739	SERP1739	-	CP000029.1:1778516-1779706	2.253265	1.48E-12	1.9E-11	UP
SE0610	SERP0610	membrane protein, TerC family	CP000029.1:603719-604525	2.295257	1.67E-12	2.13E-11	UP
SE2469	SERP2469	Alcohol dehydrogenase 1A	CP000029.1:2521511-2522638	-0.84643	1.98E-12	2.5E-11	-
SE2293	icaA	Poly-beta-1,6-N-acetyl-D-glucosamine synthase	CP000029.1:2334220-2335458	-1.74893	2.21E-12	2.77E-11	DOWN
SE0780	SERP0780	-	CP000029.1:780337-780540	-0.88174	2.39E-12	2.99E-11	-
SE2337	SERP2337	Uncharacterized protein	CP000029.1:2375760-2376383	-0.88997	2.89E-12	3.59E-11	-
SE1244	infC	translation initiation factor IF-3	CP000029.1:1278926-1279453	2.135962	2.91E-12	3.6E-11	UP
SE1400	bcp	bacterioferritin comigratory protein	CP000029.1:1464425-1464883	-1.06022	3.45E-12	4.23E-11	DOWN
SE1284	rpsD	30S ribosomal protein S4	CP000029.1:1328357-1328959	2.1112	3.55E-12	4.33E-11	UP
SE1128	dnaG	DNA primase	CP000029.1:1167172-1168968	2.12071	4.45E-12	5.4E-11	UP
SE1318	leuS	Leucine--tRNA ligase	CP000029.1:1377441-1379855	2.119262	4.57E-12	5.52E-11	UP
SE0088	SERP0088	-	CP000029.1:74748-74996	-1.35465	6.34E-12	7.56E-11	DOWN
SE0457	SERP0457	-	CP000029.1:461015-461248	-1.91377	6.33E-12	7.56E-11	DOWN
SE0822	SERP0822	-	CP000029.1:827327-827422	2.331022	7.56E-12	8.96E-11	UP
SE0347	mdh	Malate dehydrogenase	CP000029.1:346930-347871	-0.80675	8.53E-12	1E-10	-
SE0947	femB	-	CP000029.1:962794-964047	2.114893	8.54E-12	1E-10	UP
SE2314	SERP2314	YceI family protein	CP000029.1:2352715-2353230	-0.86265	9.5E-12	1.11E-10	-
SE0948	SERP0948	Uncharacterized protein	CP000029.1:965066-965830	-1.36395	1.32E-11	1.54E-10	DOWN
SE0843	SERP0843	stage III sporulation protein E	CP000029.1:852616-855009	2.10949	1.46E-11	1.68E-10	UP
SE1984	narI	Respiratory nitrate reductase, gamma subunit	CP000029.1:2001177-2001854	-0.79144	1.46E-11	1.68E-10	-
SE0066	SERP0066	-	CP000029.1:54197-54604	-0.77778	2.07E-11	2.36E-10	-
SE0396	nrdI-1	Protein NrdI	CP000029.1:398129-398527	-1.08536	2.08E-11	2.36E-10	DOWN
SE0288	SERP0288	-	CP000029.1:290114-290221	-1.45556	2.11E-11	2.39E-10	DOWN
SE2156	ldh	L-lactate dehydrogenase	CP000029.1:2183384-2184334	-0.75892	2.38E-11	2.67E-10	-
SE0132	SERP0132	veg protein	CP000029.1:123037-123300	-0.76228	2.55E-11	2.85E-10	-
SE2137	SERP2137	Possible transmembrane acyltransferase	CP000029.1:2162461-2164269	2.224244	2.86E-11	3.17E-10	UP
SE2133	SERP2133	D-lactate dehydrogenase, putative	CP000029.1:2158591-2159589	-1.16083	3.07E-11	3.39E-10	DOWN
SE0470	SERP0470	-	CP000029.1:471450-471686	-0.91233	3.33E-11	3.66E-10	-
SE1242	rplT	50S ribosomal protein L20	CP000029.1:1278290-1278646	2.040031	4.5E-11	4.93E-10	UP
SE1487	sdrH	Putative uncharacterized protein	CP000029.1:1555024-1556469	-0.79209	4.93E-11	5.36E-10	-
SE1270	SERP1270	metallo-beta-lactamase family protein	CP000029.1:1314809-1315498	-0.73765	4.98E-11	5.39E-10	-
SE2058	gntK	Gluconate kinase	CP000029.1:2083341-2084882	2.080251	5.51E-11	5.94E-10	UP
SE1381	SERP1381	conserved hypothetical protein	CP000029.1:1438769-1439113	2.086358	5.79E-11	6.21E-10	UP
SE2077	SERP2077	sodium/hydrogen exchanger family protein	CP000029.1:2103160-2104947	-0.74806	5.96E-11	6.36E-10	-
SE1316	SERP1316	Surface antigen SasC	CP000029.1:1365621-1376699	-0.77297	7.24E-11	7.68E-10	-
SE1728	rho	transcription termination factor Rho	CP000029.1:1764422-1765738	2.104255	7.35E-11	7.72E-10	UP
SE1960	tagF	Teichoic acid biosynthesis protein F, putative	CP000029.1:1978254-1980419	2.060988	7.35E-11	7.72E-10	UP
SE0604	SERP0604	conserved hypothetical protein	CP000029.1:596485-596994	-0.72154	7.94E-11	8.29E-10	-
SE0150	SERP0150	ATP-dependent zinc metalloprotease FtsH	CP000029.1:140672-142774	2.00579	9.11E-11	9.48E-10	UP
SE1129	SERP1129	conserved hypothetical protein	CP000029.1:1169142-1169960	2.081535	1.14E-10	1.18E-09	UP
SE0575	trpS	tryptophanyl-tRNA synthetase	CP000029.1:569129-570118	-0.75958	1.22E-10	1.25E-09	-
SE1797	SERP1797	NAD-dependent protein deacetylase	CP000029.1:1842789-1843529	-1.18354	1.51E-10	1.55E-09	DOWN
SE1037	SERP1037	Nucleoside diphosphate kinase	CP000029.1:1083213-1083662	-0.91652	1.85E-10	1.89E-09	-
SE0707	SERP0707	conserved hypothetical protein	CP000029.1:704902-705363	-0.74104	1.87E-10	1.89E-09	-
SE1925	SERP1925	conserved hypothetical protein	CP000029.1:1943847-1944527	-1.05337	1.87E-10	1.89E-09	DOWN
SE1201	SERP1201	Protein-export membrane protein SecDF	CP000029.1:1239114-1241405	1.973358	1.95E-10	1.96E-09	UP
SE2155	budB	acetolactate synthase, catabolic	CP000029.1:2181657-2183321	-0.68792	2.07E-10	2.07E-09	-
SE1675	SERP1675	Protein SprT-like	CP000029.1:1719919-1720374	2.424144	2.22E-10	2.21E-09	UP
SE1373	SERP1373	HIT family protein	CP000029.1:1430230-1430655	-0.77661	2.4E-10	2.38E-09	-
SE0525	SERP0525	UPF0349 protein LMOf2365_2366	CP000029.1:509829-510074	-1.87843	2.51E-10	2.48E-09	DOWN
SE0753	SERP0753	Uncharacterized protein	CP000029.1:752090-752758	-0.93235	2.54E-10	2.49E-09	-
SE1985	narJ	Respiratory nitrate reductase, delta subunit, putative	CP000029.1:2001847-2002437	-0.7134	2.59E-10	2.53E-09	-
SE1838	SERP1838	glucose uptake protein	CP000029.1:1867104-1867967	2.251757	2.74E-10	2.67E-09	UP
SE1784	SERP1784	-	CP000029.1:1829149-1829685	-0.67269	3.24E-10	3.14E-09	-
SE2184	SERP2184	-	CP000029.1:2217830-2218135	-0.84889	3.54E-10	3.42E-09	-
SE0511	lipA	Lipoyl synthase	CP000029.1:498445-499359	-0.6773	3.72E-10	3.57E-09	-
SE1240	SERP1240	-	CP000029.1:1276321-1277244	2.275473	4.3E-10	4.1E-09	UP
SE0135	SERP0135	putative endoribonuclease L-PSP	CP000029.1:125402-125782	-0.66332	4.32E-10	4.11E-09	-
SE0273	SERP0273	Uncharacterized protein	CP000029.1:278475-279251	-0.68812	4.56E-10	4.32E-09	-
SE0018	SERP0018	Esterase	CP000029.1:13768-15813	-1.21661	4.95E-10	4.66E-09	DOWN
SE2246	SERP2246	HTH-type transcriptional regulator ArcR	CP000029.1:2273928-2274614	-0.94232	5.04E-10	4.71E-09	-

SE2373	SERP2373	-	CP000029.1:2421360-2422652	-4.11994	5.04E-10	4.71E-09	DOWN
SE1888	SERP1888	Putative 2-hydroxyacid dehydrogenase SAOUHSC_025	CP000029.1:1910736-1911689	-0.66685	5.08E-10	4.72E-09	-
SE1785	SERP1785	alcohol dehydrogenase, zinc-containing	CP000029.1:1830481-1831491	-0.65847	5.17E-10	4.79E-09	-
SE0979	SERP0979	norQ protein, putative	CP000029.1:997023-997814	2.311923	5.38E-10	4.96E-09	UP
SE0978	SERP0978	conserved hypothetical protein	CP000029.1:995121-997010	2.043225	5.79E-10	5.31E-09	UP
SE2012	garK	glycerate 2-kinase 1	CP000029.1:2029466-2030617	-0.82502	5.82E-10	5.32E-09	-
SE2076	SERP2076	Fructose-1,6-bisphosphatase class 3	CP000029.1:2100973-2102937	1.977471	6.5E-10	5.91E-09	UP
SE1024	asnS	Asparagine--tRNA ligase	CP000029.1:1068035-1069327	1.933679	9.41E-10	8.53E-09	UP
SE1095	SERP1095	-	CP000029.1:1139714-1140292	-0.82689	9.48E-10	8.55E-09	-
SE1720	SERP1720	lipoprotein, putative	CP000029.1:1759007-1759531	-0.65267	9.92E-10	8.87E-09	-
SE2185	cysC	adenylylsulfate kinase	CP000029.1:2218459-2219058	2.374378	9.88E-10	8.87E-09	UP
SE1293	tyrS	Tyrosine--tRNA ligase	CP000029.1:1338682-1339947	3.781755	1.3E-09	1.16E-08	UP
SE0829	SERP0829	membrane-associated zinc metalloprotease, putative	CP000029.1:832744-834030	1.933726	1.37E-09	1.22E-08	UP
SE1983	SERP1983	-	CP000029.1:2000705-2001157	-0.69233	1.42E-09	1.25E-08	-
SE1437	gatB	Aspartyl/glutamyl-tRNA(Asn/Gln) amidotransferase sul	CP000029.1:1498931-1500358	1.915716	1.67E-09	1.47E-08	UP
SE0348	SERP0348	Uncharacterized protein	CP000029.1:348005-349561	-0.62053	1.87E-09	1.63E-08	-
SE0431	yvcD	TPR domain protein	CP000029.1:435275-436714	1.975105	1.87E-09	1.63E-08	UP
SE1672	leuD	3-isopropylmalate dehydratase, small subunit	CP000029.1:1711750-1712319	-0.838	2.62E-09	2.28E-08	-
SE0854	SERP0854	-	CP000029.1:865710-865925	-0.80632	3.01E-09	2.61E-08	-
SE1792	lacD	Tagatose 1,6-diphosphate aldolase	CP000029.1:1838483-1839460	-0.78445	3.33E-09	2.87E-08	-
SE0595	SERP0595	excinuclease ABC, A subunit	CP000029.1:588630-590888	1.921674	3.35E-09	2.87E-08	UP
SE0244	SERP0244	oxidoreductase, aldo/keto reductase family	CP000029.1:250971-251909	-0.59458	3.39E-09	2.89E-08	-
SE1277	SERP1277	thiol peroxidase	CP000029.1:1321604-1322098	-0.62455	3.5E-09	2.98E-08	-
SE0741	SERP0741	acetyltransferase, GNAT family	CP000029.1:737745-738185	-0.60097	3.59E-09	3.03E-08	-
SE1281	SERP1281	Septation ring formation regulator EzrA	CP000029.1:1325837-1327531	1.885106	3.59E-09	3.03E-08	UP
SE2049	SERP2049	oxidoreductase, short chain dehydrogenase/reductase fa	CP000029.1:2071904-2072596	-0.6351	3.76E-09	3.16E-08	-
SE2191	cysJ	sulfite reductase (NADPH) flavoprotein alpha-compone	CP000029.1:2224422-2226266	1.963146	3.95E-09	3.31E-08	UP
SE0487	SERP0487	Toprim domain protein	CP000029.1:480035-480421	-0.71931	3.99E-09	3.33E-08	-
SE0746	pbp1	Penicillin-binding protein 2B	CP000029.1:741901-744228	1.927856	4.23E-09	3.51E-08	UP
SE0664	SERP0664	-	CP000029.1:660702-660794	-0.82944	4.39E-09	3.63E-08	-
SE2201	SERP2201	putative transport protein, sodium:solute symport family	CP000029.1:2234763-2236355	1.894486	4.47E-09	3.68E-08	UP
SE0079	SERP0079	Uncharacterized protein	CP000029.1:66815-67198	1.915421	4.72E-09	3.86E-08	UP
SE0691	SERP0691	-	CP000029.1:687017-687553	-0.59534	4.71E-09	3.86E-08	-
SE0601	fabI	Enoyl-[acyl-carrier-protein] reductase [NADPH] FabI	CP000029.1:593100-593870	2.124576	4.75E-09	3.87E-08	UP
SE0743	mraZ	Transcriptional regulator MraZ	CP000029.1:740125-740556	-0.68278	5.85E-09	4.75E-08	-
SE0034	metE	5-methyltetrahydropteroyltriglutamate--homocysteine m	CP000029.1:28455-30701	-0.62362	6.98E-09	5.65E-08	-
SE1296	ccpA	catabolite control protein A	CP000029.1:1343433-1344422	-0.61696	7.06E-09	5.69E-08	-
SE0951	SERP0951	peptide/opine/nickel uptake family ABC transporter, AT	CP000029.1:967733-968509	2.019488	7.17E-09	5.75E-08	UP
SE1987	narG	respiratory nitrate reductase, alpha subunit	CP000029.1:2003958-2007641	-0.56973	7.54E-09	6.03E-08	-
SE2190	cysI	Sulfite reductase [NADPH] hemoprotein beta-compone	CP000029.1:2222684-2224402	1.868821	8.87E-09	7.07E-08	UP
SE0152	cysK	Cysteine synthase	CP000029.1:144247-145179	1.879798	9.12E-09	7.23E-08	UP
SE0354	SERP0354	-	CP000029.1:353959-354429	-0.71942	9.15E-09	7.23E-08	-
SE1938	corA	Magnesium transport protein CorA	CP000029.1:1954368-1955315	-0.68413	9.2E-09	7.25E-08	-
SE2061	SERP2061	transcriptional regulator, MerR family	CP000029.1:2087471-2088187	-0.82278	1.05E-08	8.27E-08	-
SE1690	SERP1690	D-alanine--D-alanine ligase	CP000029.1:1733954-1735027	1.940552	1.17E-08	9.14E-08	UP
SE0703	SERP0703	cell division protein FtsW	CP000029.1:697539-698762	1.945168	1.26E-08	9.8E-08	UP
SE2267	SERP2267	-	CP000029.1:2301140-2301316	-0.55116	1.39E-08	1.08E-07	-
SE1749	SERP1749	-	CP000029.1:1786960-1787370	-0.54718	1.44E-08	1.11E-07	-
SE1982	SERP1982	sensory box sensor histidine kinase	CP000029.1:1999661-2000695	-0.57752	1.44E-08	1.11E-07	-
SE1481	SERP1481	[Candida] glabrata	CP000029.1:1543045-1544334	-0.55019	1.48E-08	1.14E-07	-
SE0736	SERP0736	-	CP000029.1:736115-736279	-0.56231	1.54E-08	1.18E-07	-
SE1019	recU	Holliday junction resolvase RecU	CP000029.1:1063223-1063852	1.84815	1.54E-08	1.18E-07	UP
SE0963	lysC	aspartate kinase, monofunctional class	CP000029.1:981561-982763	2.11716	1.64E-08	1.24E-07	UP
SE1380	SERP1380	DNA repair exonuclease family protein	CP000029.1:1437302-1438498	-0.61438	1.64E-08	1.24E-07	-
SE0549	argG	Argininosuccinate synthase	CP000029.1:534348-535553	-1.39429	1.69E-08	1.27E-07	DOWN
SE1245	SERP1245	Uncharacterized protein	CP000029.1:1279661-1281163	1.878844	1.9E-08	1.43E-07	UP
SE0906	guaC	guanosine monophosphate reductase	CP000029.1:916896-917873	1.945118	1.96E-08	1.46E-07	UP
SE1794	lacB	ribose 5-phosphate isomerase B	CP000029.1:1840409-1840924	-1.38324	1.95E-08	1.46E-07	DOWN
SE0138	prsA	ribose-phosphate pyrophosphokinase	CP000029.1:128259-129224	1.82373	2.02E-08	1.5E-07	UP
SE0141	mfd	transcription-repair coupling factor	CP000029.1:130875-134384	1.845956	2.1E-08	1.56E-07	UP
SE0795	plsX	fatty acid/phospholipid synthesis protein PlsX	CP000029.1:795444-796421	2.006571	2.11E-08	1.56E-07	UP
SE1115	SERP1115	5-formyltetrahydrofolate cyclo-ligase	CP000029.1:1154793-1155344	-0.62417	2.67E-08	1.96E-07	-
SE2018	SERP2018	Membrane protein, putative	CP000029.1:2036441-2038270	1.987002	2.75E-08	2.02E-07	UP
SE1948	tcaA	-	CP000029.1:1962990-1964378	1.934367	2.83E-08	2.07E-07	UP
SE1362	SERP1362	-	CP000029.1:1418574-1419308	-1.06016	2.91E-08	2.11E-07	DOWN
SE1669	leuA	2-isopropylmalate synthase	CP000029.1:1707788-1709323	-0.58298	2.91E-08	2.11E-07	-
SE0311	SERP0311	-	CP000029.1:314542-315465	-0.62833	3.07E-08	2.22E-07	-
SE2340	SERP2340	drug resistance transporter, EmrB/QacA family	CP000029.1:2385517-2386935	-0.52389	3.09E-08	2.23E-07	-
SE1070	malA	glycosyl hydrolase family protein	CP000029.1:1114441-1116096	-0.51349	3.16E-08	2.27E-07	-
SE1719	glyA	serine hydroxymethyltransferase	CP000029.1:1757744-1758982	-0.51304	3.29E-08	2.35E-07	-
SE1377	SERP1377	-	CP000029.1:1433159-1433263	-0.62159	3.41E-08	2.43E-07	-
SE1835	SERP1835	xanthine/uracil permease family protein	CP000029.1:1863231-1864565	1.82092	3.74E-08	2.66E-07	UP
SE2157	SERP2157	transcriptional regulator, LysR family	CP000029.1:2185156-2186037	-0.83192	3.82E-08	2.71E-07	-
SE1670	leuB	3-isopropylmalate dehydrogenase	CP000029.1:1709320-1710363	-0.60713	3.99E-08	2.82E-07	-
SE0929	SERP0929	membrane protein, putative	CP000029.1:944306-945523	1.834995	4.17E-08	2.93E-07	UP
SE2334	SERP2334	Probable metabolite transport protein CsbC	CP000029.1:2371584-2372924	1.787424	4.27E-08	2.99E-07	UP

SE2271	SERP2271	-	CP000029.1:2304431-2304787	-0.75978	4.43E-08	3.1E-07	-
SE2119	SERP2119	-	CP000029.1:2144149-2144364	-0.49893	4.46E-08	3.11E-07	-
SE0389	SERP0389	aldehyde-alcohol dehydrogenase	CP000029.1:389609-392218	-0.62385	4.6E-08	3.19E-07	-
SE1370	SERP1370	-	CP000029.1:1427468-1428085	1.858061	5.09E-08	3.51E-07	UP
SE1673	ilvA	L-threonine dehydratase biosynthetic IlvA	CP000029.1:1712334-1713602	-0.64438	5.08E-08	3.51E-07	-
SE2543	SERP2543	AzlC family protein	CP000029.1:2601169-2601861	-1.02595	5.22E-08	3.59E-07	DOWN
SE0754	ylmF	Cell division protein SepF	CP000029.1:752772-753365	-0.52551	5.41E-08	3.71E-07	-
SE0061	SERP0061	2-oxo-hepta-3-ene-1,7-dioate hydratase	CP000029.1:48882-49661	-1.0023	5.54E-08	3.79E-07	DOWN
SE0840	rpsO	ribosomal protein S15	CP000029.1:847803-848072	1.808186	5.7E-08	3.88E-07	UP
SE2105	capA	capA domain protein	CP000029.1:2129263-2130336	2.331038	7.28E-08	4.94E-07	UP
SE0423	SERP0423	5'-deoxynucleotidase SO_2484	CP000029.1:426379-427020	-0.61042	7.46E-08	5.04E-07	-
SE0867	glpK	Glycerol kinase	CP000029.1:880616-882115	1.751299	7.48E-08	5.04E-07	UP
SE0885	cls-1	Cardiolipin synthase	CP000029.1:897599-899071	1.750681	8.46E-08	5.69E-07	UP
SE0011	SERP0011	transcriptional regulator, TetR family	CP000029.1:7397-7936	2.107269	8.52E-08	5.71E-07	UP
SE0065	SERP0065	-	CP000029.1:53404-54069	-0.66984	9.27E-08	6.17E-07	-
SE0876	femC	glutamine synthetase, type I	CP000029.1:890486-891826	1.740224	9.25E-08	6.17E-07	UP
SE0033	SERP0033	Uncharacterized protein	CP000029.1:27690-28433	-0.623	9.51E-08	6.31E-07	-
SE2374	SERP2374	-	CP000029.1:2422645-2423460	-2.89799	9.7E-08	6.42E-07	DOWN
SE1329	SERP1329	Uncharacterized protein	CP000029.1:1390222-1391706	1.797381	1E-07	6.61E-07	UP
SE1919	hutG	Formimidoylglutamase	CP000029.1:1939672-1940607	-0.47085	1.14E-07	7.52E-07	-
SE1444	SERP1444	Heptaprenylglyceryl phosphate synthase	CP000029.1:1509788-1510480	-0.49832	1.17E-07	7.66E-07	-
SE2200	SERP2200	-	CP000029.1:2233912-2234016	2.090734	1.18E-07	7.73E-07	UP
SE2174	SERP2174	-	CP000029.1:2204812-2205030	-0.66533	1.26E-07	8.23E-07	-
SE2195	SERP2195	dihydrolipoamide dehydrogenase	CP000029.1:2228098-2229441	-0.47939	1.33E-07	8.61E-07	-
SE1369	SERP1369	-	CP000029.1:1426403-1426906	-0.46985	1.35E-07	8.75E-07	-
SE0103	SERP0103	Hydrolase, NUDIX family	CP000029.1:88346-88732	-0.83044	1.38E-07	8.93E-07	-
SE0290	sitC	ABC transporter, substrate-binding protein, putative	CP000029.1:293030-293959	1.732062	1.4E-07	9.01E-07	UP
SE1697	SERP1697	Membrane protein insertase YidC 2	CP000029.1:1740116-1740988	1.795307	1.45E-07	9.3E-07	UP
SE0015	SERP0015	ABC transporter, ATP-binding protein	CP000029.1:10895-11581	2.893644	1.47E-07	9.4E-07	UP
SE2091	SERP2091	-	CP000029.1:2117944-2118120	-1.13774	1.63E-07	1.04E-06	DOWN
SE0156	lysS	lysyl-tRNA synthetase	CP000029.1:147452-148939	1.735476	1.68E-07	1.07E-06	UP
SE0696	SERP0696	GTP-binding protein TypA, putative	CP000029.1:691695-693542	1.744809	1.68E-07	1.07E-06	UP
SE0863	hexA	DNA mismatch repair protein MutS	CP000029.1:874143-876764	1.76729	1.7E-07	1.08E-06	UP
SE0972	SERP0972	cold shock protein CspC	CP000029.1:990579-990812	1.726818	1.78E-07	1.12E-06	UP
SE2363	brnQ-2	Branched-chain amino acid transport system carrier pro	CP000029.1:2408310-2409668	1.77771	1.79E-07	1.13E-06	UP
SE0672	cydA	Cytochrome d ubiquinol oxidase, subunit I, putative	CP000029.1:667579-668934	-0.68448	1.95E-07	1.22E-06	-
SE1958	SERP1958	Teichoic acid biosynthesis protein F, putative	CP000029.1:1975667-1977346	1.775516	1.98E-07	1.24E-06	UP
SE2284	SERP2284	Phosphonates ABC transporter, permease protein CC03	CP000029.1:2324726-2325526	2.202703	2.02E-07	1.26E-06	UP
SE1072	SERP1072	peptidase T	CP000029.1:1117752-1118876	-0.50201	2.06E-07	1.28E-06	-
SE2186	sat	sulfate adenylyltransferase	CP000029.1:2219074-2220252	1.787648	2.1E-07	1.3E-06	UP
SE2199	SERP2199	-	CP000029.1:2232343-2233452	1.769863	2.11E-07	1.3E-06	UP
SE0529	SERP0529	Putative histidine porter YuiF	CP000029.1:513929-515245	1.953293	2.13E-07	1.31E-06	UP
SE1981	SERP1981	DNA-binding response regulator, LuxR family	CP000029.1:1998976-1999632	-0.47453	2.15E-07	1.32E-06	-
SE2176	betA	Oxygen-dependent choline dehydrogenase	CP000029.1:2206974-2208692	-0.44991	2.18E-07	1.33E-06	-
SE0337	SERP0337	-	CP000029.1:338622-339302	-0.45995	2.52E-07	1.54E-06	-
SE0926	parC	DNA topoisomerase 4 subunit A	CP000029.1:939046-941448	1.772118	2.53E-07	1.54E-06	UP
SE1671	leuC	3-isopropylmalate dehydratase, large subunit	CP000029.1:1710379-1711749	-0.54119	2.55E-07	1.55E-06	-
SE1791	lacF	PTS system, lactose-specific IIA component	CP000029.1:1838149-1838463	-0.89503	2.68E-07	1.62E-06	-
SE0760	SERP0760	glyoxalase family protein	CP000029.1:759097-759894	-2.79846	2.71E-07	1.63E-06	DOWN
SE1691	SERP1691	cell division protein, FtsW/RodA/SpoVE family	CP000029.1:1735418-1736629	1.781398	2.71E-07	1.63E-06	UP
SE2052	SERP2052	Uncharacterized protein	CP000029.1:2073731-2076589	1.939838	2.81E-07	1.68E-06	UP
SE1776	SERP1776	iron compound ABC transporter, permease protein	CP000029.1:1819969-1821000	1.965151	2.88E-07	1.72E-06	UP
SE0408	SERP0408	conserved hypothetical protein	CP000029.1:408970-409290	-0.45498	2.95E-07	1.76E-06	-
SE1048	SERP1048	-	CP000029.1:1094204-1095586	-0.42838	3.03E-07	1.8E-06	-
SE0921	acnA	Aconitate hydratase A	CP000029.1:932351-935056	-0.42504	3.07E-07	1.82E-06	-
SE2285	SERP2285	phosphonate ABC transporter, ATP-binding protein	CP000029.1:2325528-2326301	3.617857	3.13E-07	1.85E-06	UP
SE0438	SERP0438	cell division inhibitor-like protein	CP000029.1:442259-443158	-0.42589	3.27E-07	1.93E-06	-
SE2073	SERP2073	thiazole biosynthesis protein ThiG	CP000029.1:2098310-2099077	-0.74816	3.36E-07	1.98E-06	-
SE2069	SERP2069	Uncharacterized protein	CP000029.1:2094468-2095742	1.693522	3.44E-07	2.02E-06	UP
SE2297	geh-1	Esterase	CP000029.1:2337843-2339909	1.69074	3.71E-07	2.17E-06	UP
SE0162	ctsR	Transcriptional regulator CtsR	CP000029.1:166946-167407	-0.46201	3.76E-07	2.19E-06	-
SE1087	accC	acetyl-CoA carboxylase, biotin carboxylase	CP000029.1:1131620-1132978	1.748538	3.84E-07	2.23E-06	UP
SE2143	SERP2143	Uncharacterized protein	CP000029.1:2170608-2171465	-0.44121	3.83E-07	2.23E-06	-
SE0955	SERP0955	Uncharacterized protein	CP000029.1:971082-972893	1.807168	3.89E-07	2.25E-06	UP
SE0903	katA	Catalase	CP000029.1:914553-916067	-0.41114	4.29E-07	2.47E-06	-
SE1324	putA	Uncharacterized protein	CP000029.1:1385177-1386178	-0.48368	4.41E-07	2.53E-06	-
SE0739	SERP0739	-	CP000029.1:736711-736845	-0.41419	4.59E-07	2.63E-06	-
SE1978	SERP1978	nitroreductase family protein	CP000029.1:1995611-1996240	2.350791	4.61E-07	2.64E-06	UP
SE0163	SERP0163	Protein-arginine kinase activator protein	CP000029.1:167413-167991	-0.44269	4.68E-07	2.67E-06	-
SE2158	SERP2158	Uncharacterized protein	CP000029.1:2186581-2188032	-0.42686	5.09E-07	2.9E-06	-
SE0121	SERP0121	-	CP000029.1:113683-114012	-0.41379	5.44E-07	3.09E-06	-
SE0304	SERP0304	Uncharacterized protein	CP000029.1:306171-307400	1.705114	5.5E-07	3.11E-06	UP
SE0563	SERP0563	putative lipopolysaccharide modification acyltransferase	CP000029.1:553646-555469	1.762261	5.76E-07	3.25E-06	UP
SE1433	dinP	DNA polymerase IV	CP000029.1:1494240-1495310	-0.51173	5.82E-07	3.27E-06	-
SE2388	geh-2	Esterase	CP000029.1:2438258-2440189	-0.5029	6.1E-07	3.42E-06	-
SE1902	SERP1902	Uncharacterized protein	CP000029.1:1923989-1925365	1.688874	7.09E-07	3.96E-06	UP

SE2022	SERP2022	2-dehydropantoate 2-reductase	CP000029.1:2041194-2042126	-0.78117	7.1E-07	3.96E-06	-
SE2398	aap	Cell surface protein, putative	CP000029.1:2454490-2461683	-0.38882	7.38E-07	4.11E-06	-
SE2118	SERP2118	transcriptional regulator, LysR family	CP000029.1:2143077-2143961	-0.41642	8.02E-07	4.46E-06	-
SE0747	mraY	phospho-N-acetylmuramoyl-pentapeptide-transferase	CP000029.1:744417-745382	1.69932	8.4E-07	4.66E-06	UP
SE2187	SERP2187	membrane protein, putative	CP000029.1:2220276-2221175	1.742981	8.44E-07	4.66E-06	UP
SE1279	thiI	thiamine biosynthesis protein ThiI	CP000029.1:1323145-1324368	-0.40399	9.19E-07	5.06E-06	-
SE1233	hemD	uroporphyrinogen-III synthase	CP000029.1:1268567-1269247	1.845196	9.62E-07	5.29E-06	UP
SE1143	rpsU	30S ribosomal protein S21	CP000029.1:1179860-1180036	-0.41325	9.66E-07	5.3E-06	-
SE0564	clpB	Chaperone protein ClpB	CP000029.1:555678-558287	-0.3895	9.8E-07	5.34E-06	-
SE0878	SERP0878	-	CP000029.1:892406-892645	2.209023	9.78E-07	5.34E-06	UP
SE0553	spsB	Signal peptidase I	CP000029.1:538668-539243	1.998447	1.06E-06	5.76E-06	UP
SE0977	brnQ-1	Putative branched-chain amino acid carrier protein SA(	CP000029.1:993591-994934	1.692027	1.08E-06	5.87E-06	UP
SE0732	SERP0732	succinate dehydrogenase, iron-sulfur protein	CP000029.1:732572-733414	-0.37284	1.1E-06	5.97E-06	-
SE0737	SERP0737	-	CP000029.1:736335-736469	-0.3725	1.18E-06	6.37E-06	-
SE1770	SERP1770	AGAP007889-PA	CP000029.1:1814503-1815690	-0.43397	1.21E-06	6.51E-06	-
SE1866	SERP1866	Putative acyl-CoA dehydrogenase YdbM	CP000029.1:1892115-1893260	1.939565	1.21E-06	6.51E-06	UP
SE0609	prfC	Peptide chain release factor 3	CP000029.1:601848-603410	1.728428	1.23E-06	6.57E-06	UP
SE0848	SERP0848	ACT domain protein	CP000029.1:859170-859997	-0.4822	1.23E-06	6.6E-06	-
SE1010	SERP1010	RNase H	CP000029.1:1022964-1023359	-1.06307	1.26E-06	6.7E-06	DOWN
SE0136	spoVG	Putative septation protein SpoVG 1	CP000029.1:125842-126150	-0.36795	1.29E-06	6.83E-06	-
SE1071	gnd	6-phosphogluconate dehydrogenase, decarboxylating	CP000029.1:1116266-1117672	1.643519	1.3E-06	6.87E-06	UP
SE0164	SERP0164	Protein-arginine kinase	CP000029.1:167981-168988	-0.39248	1.33E-06	7.04E-06	-
SE0827	uppS	undecaprenyl diphosphate synthase	CP000029.1:830954-831724	-0.85458	1.4E-06	7.4E-06	-
SE0841	pnp	Polyribonucleotide nucleotidyltransferase	CP000029.1:848204-850309	1.633426	1.52E-06	7.99E-06	UP
SE0062	SERP0062	NAD(P)H-flavin oxidoreductase	CP000029.1:49802-50557	-0.37393	1.53E-06	8.01E-06	-
SE1980	SERP1980	nitrate transporter	CP000029.1:1997556-1998719	1.652554	1.61E-06	8.43E-06	UP
SE0786	SERP0786	Uncharacterized protein	CP000029.1:785323-787323	1.743116	1.67E-06	8.73E-06	UP
SE0858	SERP0858	-	CP000029.1:870209-870826	-0.52977	1.77E-06	9.22E-06	-
SE0568	fabF	3-oxoacyl-(acyl-carrier-protein) synthase II	CP000029.1:561455-562699	1.642046	1.82E-06	9.43E-06	UP
SE0758	ileS	Isoleucine--tRNA ligase	CP000029.1:755578-758328	1.626638	1.82E-06	9.43E-06	UP
SE1732	fbaA	fructose-bisphosphate aldolase, class II	CP000029.1:1771077-1771937	1.647527	1.84E-06	9.53E-06	UP
SE0641	SERP0641	Protein FmtA	CP000029.1:635463-636665	2.237911	1.87E-06	9.65E-06	UP
SE0142	SERP0142	Uncharacterized protein	CP000029.1:134374-135915	1.66495	1.89E-06	9.75E-06	UP
SE1754	SERP1754	Putative protein of unknown function	CP000029.1:1791022-1791678	-0.35269	1.95E-06	1E-05	-
SE0327	SERP0327	conserved hypothetical protein	CP000029.1:331297-331890	-0.45972	1.96E-06	1.01E-05	-
SE0430	SERP0430	bacterial transferase hexapeptide domain protein	CP000029.1:434781-435308	1.70184	2.19E-06	1.12E-05	UP
SE0094	SERP0094	Uncharacterized protein	CP000029.1:79505-80413	3.096627	2.28E-06	1.16E-05	UP
SE1289	SERP1289	-	CP000029.1:1333448-1334572	1.804094	2.43E-06	1.24E-05	UP
SE1752	manA-1	Mannose-6-phosphate isomerase	CP000029.1:1789442-1790380	-0.52524	2.46E-06	1.25E-05	-
SE0796	fabD	malonyl CoA-acyl carrier protein transacylase	CP000029.1:796423-797352	1.698573	2.56E-06	1.3E-05	UP
SE0787	SERP0787	Putative ribosome biogenesis GTPase RsgA 1	CP000029.1:787632-788507	-0.66898	2.6E-06	1.31E-05	-
SE0862	SERP0862	-	CP000029.1:873451-873945	1.765566	2.6E-06	1.31E-05	UP
SE0722	pheT	Phenylalanine--tRNA ligase beta subunit	CP000029.1:718648-721050	1.618263	2.69E-06	1.35E-05	UP
SE1988	SERP1988	Uroporphyrin-III C-methyltransferase, putative	CP000029.1:2007820-2008755	-0.346	2.84E-06	1.42E-05	-
SE1935	gltS	sodium/glutamate symporter	CP000029.1:1951916-1953124	-0.34496	3.03E-06	1.52E-05	-
SE0868	glpD	glycerol-3-phosphate dehydrogenase, aerobic	CP000029.1:882292-883965	1.607598	3.14E-06	1.57E-05	UP
SE0670	ptsI	phosphoenolpyruvate-protein phosphotransferase	CP000029.1:665150-666868	1.592697	3.34E-06	1.66E-05	UP
SE2102	SERP2102	glucose uptake protein	CP000029.1:2126368-2127249	1.636924	3.59E-06	1.79E-05	UP
SE1310	SERP1310	peptidase, M20/M25/M40 family	CP000029.1:1359201-1360610	1.663169	3.66E-06	1.81E-05	UP
SE2209	SERP2209	putative NAD(P)-dependent oxidoreductase YbjT	CP000029.1:2244112-2245656	-0.3259	4.08E-06	2.02E-05	-
SE1896	SERP1896	CAAX amino terminal protease family protein	CP000029.1:1918294-1919040	1.871177	4.13E-06	2.03E-05	UP
SE2054	SERP2054	Glycosyltransferase Gtf1	CP000029.1:2077256-2078746	1.780906	4.2E-06	2.07E-05	UP
SE0602	SERP0602	membrane protein, putative	CP000029.1:594267-595352	1.660627	4.28E-06	2.1E-05	UP
SE1121	SERP1121	Uncharacterized protein	CP000029.1:1159698-1160546	1.640253	4.37E-06	2.14E-05	UP
SE0158	SERP0158	Pyridoxal 5'-phosphate synthase subunit PdxS	CP000029.1:162243-163130	-0.34658	4.41E-06	2.15E-05	-
SE2168	mqo-2	malate:quinone-oxidoreductase	CP000029.1:2196899-2198398	-0.31295	4.48E-06	2.18E-05	-
SE0847	SERP0847	oxidoreductase, short-chain dehydrogenase/reductase fa	CP000029.1:858313-859017	2.07469	4.61E-06	2.24E-05	UP
SE1187	SERP1187	Uncharacterized protein	CP000029.1:1223269-1224264	-0.33606	4.74E-06	2.3E-05	-
SE1431	SERP1431	ferritin	CP000029.1:1492966-1493466	-0.31125	4.86E-06	2.35E-05	-
SE1398	SERP1398	transcriptional regulator, Fur family	CP000029.1:1462924-1463376	-0.31443	4.96E-06	2.39E-05	-
SE0093	SERP0093	-	CP000029.1:78663-79202	-0.41179	5.31E-06	2.55E-05	-
SE1799	rplM	50S ribosomal protein L13	CP000029.1:1844203-1844640	1.636466	5.3E-06	2.55E-05	UP
SE0293	sirR	DtxR family transcriptional regulator	CP000029.1:295659-296303	2.076222	5.47E-06	2.63E-05	UP
SE2323	SERP2323	-	CP000029.1:2359862-2360017	-1.10635	5.61E-06	2.69E-05	DOWN
SE0902	SERP0902	Uncharacterized protein	CP000029.1:912874-914322	1.666457	5.81E-06	2.78E-05	UP
SE1908	SERP1908	-	CP000029.1:1927761-1928291	-0.37217	5.82E-06	2.78E-05	-
SE0726	SERP0726	PHP domain protein	CP000029.1:723150-724859	1.865858	5.86E-06	2.79E-05	UP
SE1957	SERP1957	L-lactate permease	CP000029.1:1973716-1975317	1.571819	5.92E-06	2.81E-05	UP
SE0901	SERP0901	-	CP000029.1:912254-912568	-0.50379	5.99E-06	2.84E-05	-
SE1124	SERP1124	DEAD-box ATP-dependent RNA helicase CshB	CP000029.1:1162480-1163826	1.602766	6.04E-06	2.85E-05	UP
SE0060	ahpC	alkyl hydroperoxide reductase, subunit C	CP000029.1:47868-48437	-0.29867	6.18E-06	2.91E-05	-
SE0826	frr	Ribosome-recycling factor	CP000029.1:830179-830733	1.582408	6.23E-06	2.93E-05	UP
SE0896	SERP0896	Aspartokinase 3	CP000029.1:906216-907592	-0.67539	6.33E-06	2.97E-05	-
SE0705	ctaA	cytochrome aa3 controlling protein	CP000029.1:702756-703664	-0.38126	6.4E-06	2.99E-05	-
SE0419	SERP0419	ribosomal subunit interface protein	CP000029.1:419849-420418	-0.29833	6.47E-06	3.02E-05	-
SE0570	oppB	Uncharacterized protein	CP000029.1:563383-564309	-0.71994	6.49E-06	3.03E-05	-

SE0934	dmpI	2-hydroxymuconate tautomerase	CP000029.1:950674-950859	-0.40806	6.51E-06	3.03E-05	-
SE0137	glmU	UDP-N-acetylglucosamine pyrophosphorylase	CP000029.1:126760-128115	1.64621	6.92E-06	3.21E-05	UP
SE1025	SERP1025	DnaQ family exonuclease/DinG family helicase, putativ	CP000029.1:1069448-1072156	1.591825	7.08E-06	3.28E-05	UP
SE1352	metK	S-adenosylmethionine synthase	CP000029.1:1408320-1409519	1.61538	7.12E-06	3.29E-05	UP
SE1777	SERP1777	ferric citrate ABC transporter periplasmic binding prote	CP000029.1:1821012-1822007	1.633173	7.4E-06	3.41E-05	UP
SE1139	SERP1139	-	CP000029.1:1176601-1177284	1.578115	7.52E-06	3.46E-05	UP
SE0299	SERP0299	Putative glycosyltransferase TagX	CP000029.1:301577-302641	2.001559	7.54E-06	3.46E-05	UP
SE1005	SERP1005	Uncharacterized protein	CP000029.1:1019468-1020517	1.637743	7.56E-06	3.46E-05	UP
SE0807	rplS	50S ribosomal protein L19	CP000029.1:808032-808382	1.558665	9.02E-06	4.12E-05	UP
SE0844	SERP0844	transcriptional regulator, GntR family	CP000029.1:855012-855725	2.025389	9.11E-06	4.16E-05	UP
SE1782	SERP1782	conserved hypothetical protein	CP000029.1:1828323-1828823	-0.28157	9.48E-06	4.31E-05	-
SE0332	SERP0332	-	CP000029.1:335770-336192	-0.57152	9.63E-06	4.37E-05	-
SE2295	icaB	polysaccharide deacetylase family protein	CP000029.1:2335724-2336593	-1.31286	1.03E-05	4.69E-05	DOWN
SE0673	cydB	cytochrome d ubiquinol oxidase, subunit II, putative	CP000029.1:668931-669947	-0.60501	1.06E-05	4.78E-05	-
SE1004	SERP1004	ABC transporter, ATP-binding protein	CP000029.1:1018790-1019452	1.932486	1.08E-05	4.86E-05	UP
SE0509	SERP0509	putative membrane protein	CP000029.1:496124-496951	1.748504	1.11E-05	4.98E-05	UP
SE0064	SERP0064	-	CP000029.1:52376-53278	-0.31195	1.11E-05	5E-05	-
SE0081	SERP0081	-	CP000029.1:68810-69073	-0.70617	1.12E-05	5E-05	-
SE1833	SERP1833	-	CP000029.1:1862552-1862665	3.501002	1.15E-05	5.13E-05	UP
SE1891	SERP1891	Gp58	CP000029.1:1913337-1914113	1.891732	1.15E-05	5.14E-05	UP
SE1285	SERP1285	glycerophosphoryl diester phosphodiesterase family pro	CP000029.1:1329229-1329975	1.729947	1.16E-05	5.17E-05	UP
SE0388	SERP0388	Uncharacterized protein	CP000029.1:389003-389542	1.896008	1.16E-05	5.18E-05	UP
SE2111	SERP2111	Uncharacterized protein	CP000029.1:2134903-2135229	-0.37029	1.17E-05	5.2E-05	-
SE1235	SERP1235	Uncharacterized protein	CP000029.1:1270261-1271073	1.676097	1.19E-05	5.26E-05	UP
SERP_Ser	SERP_Sern	-	CP000029.1:1061154-1061554	-0.27807	1.24E-05	5.49E-05	-
SE0545	rocD	Ornithine aminotransferase	CP000029.1:529033-530223	-0.27114	1.29E-05	5.7E-05	-
SE1718	upp	Uracil phosphoribosyltransferase	CP000029.1:1757086-1757715	-0.27021	1.32E-05	5.79E-05	-
SE2107	capB	-	CP000029.1:2130814-2131971	2.045622	1.35E-05	5.9E-05	UP
SE0314	SERP0314	efflux ABC transporter, ATP-binding protein	CP000029.1:317337-318098	1.645408	1.41E-05	6.19E-05	UP
SE0830	proS	Proline--tRNA ligase	CP000029.1:834049-835752	1.555714	1.48E-05	6.48E-05	UP
SE0935	SERP0935	impB/mucB/samB family protein	CP000029.1:951003-952265	-0.53452	1.49E-05	6.49E-05	-
SE1252	coaE	Dephospho-CoA kinase	CP000029.1:1289032-1289643	-0.30947	1.5E-05	6.51E-05	-
SE1133	SERP1133	DNA repair protein RecO	CP000029.1:1172452-1173213	1.552465	1.55E-05	6.74E-05	UP
SE0190	SERP0190	N-acyl-L-amino acid amidohydrolase	CP000029.1:198598-199767	-0.6848	1.58E-05	6.84E-05	-
SE1287	SERP1287	soluble hydrogenase, 42 kDa subunit	CP000029.1:1330620-1331783	-0.35947	1.71E-05	7.38E-05	-
SE1260	SERP1260	Uncharacterized protein	CP000029.1:1300307-1301665	1.524222	1.72E-05	7.41E-05	UP
SE1742	SERP1742	-	CP000029.1:1781739-1782089	-0.27097	1.74E-05	7.48E-05	-
SE1412	SERP1412	penicillin-binding domain protein	CP000029.1:1476585-1477394	2.155926	1.8E-05	7.73E-05	UP
SE0229	SERP0229	-	CP000029.1:237426-237785	1.855907	1.85E-05	7.95E-05	UP
SE1375	SERP1375	-	CP000029.1:1431269-1431829	1.609117	1.86E-05	7.97E-05	UP
SE0626	SERP0626	Uncharacterized protein	CP000029.1:617221-617778	-0.35165	1.93E-05	8.25E-05	-
SE0744	mraW	Ribosomal RNA small subunit methyltransferase H	CP000029.1:740571-741506	-0.25457	1.96E-05	8.35E-05	-
SE0693	SERP0693	conserved hypothetical protein	CP000029.1:689242-689853	-0.25767	2.04E-05	8.69E-05	-
SE1941	SERP1941	Esterase/lipase	CP000029.1:1956426-1957325	-0.36672	2.14E-05	9.11E-05	-
SE2048	SERP2048	acetylornitine deacetylase, putative	CP000029.1:2070559-2071815	-0.27265	2.16E-05	9.17E-05	-
SE0400	SERP0400	vibriobactin and enterobactin ABC transporter, permeas	CP000029.1:401936-402910	1.921647	2.17E-05	9.19E-05	UP
SE1140	SERP1140	UPF0365 protein LMOf2365_0404	CP000029.1:1177300-1178289	1.520231	2.21E-05	9.32E-05	UP
SE1193	hisS	Histidine--tRNA ligase	CP000029.1:1229867-1231141	1.805568	2.28E-05	9.59E-05	UP
SE2094	sdhA	L-serine dehydratase, iron-sulfur-dependent, alpha subu	CP000029.1:2118833-2119732	-0.24208	2.49E-05	0.000105	-
SE1082	ispA	Lin1400 protein	CP000029.1:1128347-1129228	1.566282	2.58E-05	0.000108	UP
SE0226	thiD-1	phosphomethylpyrimidine kinase	CP000029.1:234682-235515	-0.24777	2.65E-05	0.000111	-
SE0462	SERP0462	-	CP000029.1:465190-465366	-0.65274	2.72E-05	0.000114	-
SE1046	ansA	L-asparaginase	CP000029.1:1091905-1092873	1.938333	2.76E-05	0.000115	UP
SE1309	dat	D-alanine aminotransferase	CP000029.1:1358349-1359197	1.528208	2.97E-05	0.000124	UP
SE0092	SERP0092	sodium-dependent transporter, putative	CP000029.1:77156-78484	1.570781	2.99E-05	0.000124	UP
SE2534	yycF	Transcriptional regulatory protein YycF	CP000029.1:2591085-2591786	-0.33486	3.05E-05	0.000127	-
SE1798	rpsI	30S ribosomal protein S9	CP000029.1:1843791-1844183	1.527039	3.08E-05	0.000128	UP
SE1492	agrC	Accessory gene regulator protein C	CP000029.1:1559116-1560405	-0.22904	3.16E-05	0.00013	-
SE2043	SERP2043	peptidase, M42 family	CP000029.1:2064057-2065133	-0.24894	3.15E-05	0.00013	-
SE0846	SERP0846	Uncharacterized protein	CP000029.1:857027-858316	1.623061	3.22E-05	0.000132	UP
SE1972	SERP1972	Transcriptional regulator, putative	CP000029.1:1990253-1992358	1.99047	3.3E-05	0.000136	UP
SE1264	accD	acetyl-CoA carboxylase, carboxyl transferase, beta subu	CP000029.1:1305917-1306774	1.776783	3.39E-05	0.000139	UP
SE0752	SERP0752	conserved hypothetical protein TIGR00726	CP000029.1:751273-752064	-0.32981	3.5E-05	0.000143	-
SE1335	SERP1335	-	CP000029.1:1394621-1395064	1.585026	3.51E-05	0.000143	UP
SE1658	SERP1658	ABC transporter, ATP-binding protein	CP000029.1:1695953-1697887	1.801859	3.62E-05	0.000148	UP
SE1292	SERP1292	serine protease	CP000029.1:1337253-1338491	-0.24208	3.63E-05	0.000148	-
SE0068	pbuX	xanthine permease	CP000029.1:55779-57047	1.539134	3.8E-05	0.000154	UP
SE0611	SERP0611	serine protease	CP000029.1:604736-606493	1.69027	3.8E-05	0.000154	UP
SE1358	menE	AMP-binding protein	CP000029.1:1414258-1415682	1.63554	3.8E-05	0.000154	UP
SE1422	vraR	DNA-binding response regulator	CP000029.1:1484739-1485368	1.598568	3.88E-05	0.000157	UP
SE2537	dnaB	Replicative DNA helicase	CP000029.1:2594401-2595801	1.499283	3.91E-05	0.000157	UP
SE1962	SERP1962	Quinone oxidoreductase, YhdH/YhfP family	CP000029.1:1981471-1982463	-0.24216	3.93E-05	0.000158	-
SE1372	SERP1372	ABC transporter, ATP-binding protein EcsA	CP000029.1:1429360-1430100	1.687959	3.98E-05	0.00016	UP
SE1049	recQ-2	ATP-dependent DNA helicase RecQ, putative	CP000029.1:1095743-1097122	-0.24991	3.99E-05	0.00016	-
SE0357	SERP0357	transcriptional regulator, DeoR family	CP000029.1:355256-356014	1.970626	4.11E-05	0.000164	UP
SE1493	agrA	Putative response regulator of the competence regulon,	CP000029.1:1560467-1561138	-0.21434	4.13E-05	0.000165	-

SE0566	SERP0566	-	CP000029.1:560026-560220	-0.25935	4.21E-05	0.000168	-
SE0772	SERP0772	-	CP000029.1:772066-772272	1.573958	4.29E-05	0.000171	UP
SE1411	SERP1411	Regulatory protein RecX	CP000029.1:1475504-1476307	-0.39971	4.39E-05	0.000174	-
SE1172	SERP1172	conserved hypothetical protein TIGR00370	CP000029.1:1207028-1207759	-0.81873	4.63E-05	0.000184	-
SE0420	secA	preprotein translocase, SecA subunit	CP000029.1:421084-423618	1.491705	4.71E-05	0.000186	UP
SE0168	gltX	Glutamate--tRNA ligase	CP000029.1:174830-176284	1.486146	4.72E-05	0.000186	UP
SE2189	SERP2189	Uroporphyrin-III C-methyltransferase	CP000029.1:2221883-2222662	1.669483	4.75E-05	0.000187	UP
SE1427	SERP1427	conserved hypothetical protein	CP000029.1:1488590-1489576	1.759654	4.9E-05	0.000193	UP
SE1956	SERP1956	WbcM protein	CP000029.1:1972366-1973511	-0.31104	5.08E-05	0.000199	-
SE0879	SERP0879	-	CP000029.1:892710-894056	1.920575	5.24E-05	0.000205	UP
SE1478	SERP1478	transcriptional regulator, GntR family	CP000029.1:1541708-1542085	-0.23924	5.23E-05	0.000205	-
SE0484	SERP0484	conserved hypothetical protein	CP000029.1:478868-479221	-0.37926	5.35E-05	0.000209	-
SE1800	truA	tRNA pseudouridine synthase A	CP000029.1:1844901-1845704	1.591516	5.78E-05	0.000225	UP
SE1832	rpsJ	30S ribosomal protein S10	CP000029.1:1862112-1862420	1.483372	5.77E-05	0.000225	UP
SE0852	recA	Protein RecA	CP000029.1:862787-863836	-0.20727	5.79E-05	0.000225	-
SE0374	pabA	Para-aminobenzoate synthase, glutamine amidotransfera	CP000029.1:371043-371630	-0.80379	6.13E-05	0.000237	-
SE0383	SERP0383	ABC transporter, ATP-binding protein	CP000029.1:381266-383149	-0.65387	6.21E-05	0.00024	-
SE2371	SERP2371	Peptide ABC transporter, permease protein, putative	CP000029.1:2418763-2419698	-0.86162	6.22E-05	0.00024	-
SE0024	SERP0024	Signal peptidase I S	CP000029.1:20149-20727	3.009166	6.27E-05	0.000242	UP
SE0269	SERP0269	Uncharacterized protein	CP000029.1:275792-276589	1.619481	6.33E-05	0.000243	UP
SE1856	moaC	Cyclic pyranopterin monophosphate synthase accessory	CP000029.1:1884016-1884504	-0.23414	6.66E-05	0.000256	-
SE0849	SERP0849	Transmembrane cell shape protein RodZ	CP000029.1:860016-860408	-0.32298	6.73E-05	0.000258	-
SE1920	SERP1920	-	CP000029.1:1940855-1942003	1.487194	6.78E-05	0.000259	UP
SE1952	SERP1952	ABC transporter, permease protein, putative	CP000029.1:1967206-1968258	-0.74699	6.78E-05	0.000259	-
SE0200	SERP0200	Putative monooxygenase YxeK	CP000029.1:209974-211278	-0.20208	6.81E-05	0.000259	-
SE0298	SERP0298	Putative CDP-glycerol:glycerophosphate glycerophosph	CP000029.1:300486-301577	1.596874	6.87E-05	0.000261	UP
SE1308	SERP1308	aminoglycoside phosphotransferase family protein	CP000029.1:1357171-1357962	1.654941	6.92E-05	0.000263	UP
SE2162	SERP2162	-	CP000029.1:2191498-2192088	-0.3594	7.13E-05	0.00027	-
SE0035	SERP0035	homocysteine S-methyltransferase domain protein/meth	CP000029.1:30698-32536	-0.41667	7.46E-05	0.000282	-
SE1228	valS	Valine--tRNA ligase	CP000029.1:1261239-1263869	1.461426	7.49E-05	0.000283	UP
SE1452	pheA	Chorismate mutase/prephenate dehydratase	CP000029.1:1517368-1518171	-0.35769	7.61E-05	0.000287	-
SE2235	SERP2235	-	CP000029.1:2266163-2266258	-2.0614	7.68E-05	0.000289	DOWN
SE1927	SERP1927	Ribulokinase	CP000029.1:1946198-1947808	-0.22932	7.86E-05	0.000295	-
SE0128	metS	Methionine--tRNA ligase	CP000029.1:118526-120496	1.458333	7.98E-05	0.000299	UP
SE2002	SERP2002	-	CP000029.1:2020849-2020995	Inf (+)	8.08E-05	0.000302	UP
SE2282	SERP2282	-	CP000029.1:2322337-2323638	1.672863	8.26E-05	0.000309	UP
SE0522	SERP0522	nifU domain protein	CP000029.1:507623-507865	-0.24681	8.76E-05	0.000326	-
SE0952	SERP0952	Putative oligopeptide transport system permease protein	CP000029.1:968472-969326	1.638582	8.76E-05	0.000326	UP
SE1976	gltT	proton/sodium-glutamate symporter	CP000029.1:1993677-1994957	1.450331	8.79E-05	0.000327	UP
SE0090	SERP0090	Uncharacterized protein	CP000029.1:76026-76754	2.093152	8.86E-05	0.000329	UP
SE1668	ilvC	ketol-acid reductoisomerase	CP000029.1:1706761-1707765	-0.25755	9.49E-05	0.000351	-
SE2074	SERP2074	Molybdopterin biosynthesis MoeB protein	CP000029.1:2099079-2100077	-0.39807	9.6E-05	0.000355	-
SE1931	SERP1931	Putative 3-methyladenine DNA glycosylase	CP000029.1:1949150-1949767	-0.51067	0.000102	0.000376	-
SE0668	SERP0668	-	CP000029.1:664186-664725	1.532663	0.000104	0.000383	UP
SE2045	SERP2045	Oxidoreductase, short chain dehydrogenase/reductase fa	CP000029.1:2065501-2066319	-0.17937	0.000106	0.000389	-
SE1924	rpiA	ribose 5-phosphate isomerase	CP000029.1:1942989-1943681	-1.1888	0.000112	0.000411	DOWN
SE1795	lacA	ribose 5-phosphate isomerase B	CP000029.1:1840939-1841367	-1.52686	0.000112	0.000412	DOWN
SE2120	SERP2120	Putative secreted antigen GbpB/SagA putative peptidog	CP000029.1:2144622-2145053	-0.99088	0.000117	0.000428	-
SE2099	xylB	Xylulose kinase	CP000029.1:2123184-2124674	1.503142	0.000122	0.000444	UP
SE1120	SERP1120	transcriptional regulator, Fur family	CP000029.1:1159276-1159695	1.505404	0.000125	0.000456	UP
SE0385	SERP0385	glycine betaine/L-proline ABC transporter, ATP-binding	CP000029.1:385141-386115	-0.16647	0.000131	0.000477	-
SE0842	SERP0842	Ribonuclease J 2	CP000029.1:850644-852350	1.43237	0.000131	0.000477	UP
SE2269	SERP2269	Uncharacterized protein	CP000029.1:2302067-2302756	-0.18655	0.000134	0.000487	-
SE2096	SERP2096	membrane protein PfoR	CP000029.1:2120428-2121471	-0.15382	0.000136	0.000492	-
SE1397	sspA	-	CP000029.1:1453570-1454418	-0.84408	0.000141	0.000509	-
SE1989	nirD	nitrite reductase [NAD(P)H], small subunit	CP000029.1:2008746-2009060	-0.3333	0.000144	0.00052	-
SE0421	prfB	Peptide chain release factor 2	CP000029.1:423814-424930	1.551998	0.000145	0.000521	UP
SE0891	SERP0891	SPBc2 prophage-derived endonuclease YokF	CP000029.1:903067-903603	3.218639	0.000151	0.00054	UP
SE2059	gntR	gluconate operon transcriptional repressor	CP000029.1:2084906-2085586	1.860992	0.00015	0.00054	UP
SE2309	SERP2309	transcriptional regulator, AsnC family	CP000029.1:2348006-2348416	-0.39666	0.000151	0.00054	-
SE0647	SERP0647	chitinase A	CP000029.1:642450-642767	-0.16785	0.000154	0.000552	-
SE1350	SERP1350	oxidoreductase, aldo/keto reductase family	CP000029.1:1406293-1407129	-0.15233	0.000155	0.000552	-
SE1022	nth	endonuclease III	CP000029.1:1066607-1067266	1.675851	0.000159	0.000569	UP
SE0208	SERP0208	Glycosyltransferase Gtf1	CP000029.1:219501-221003	-0.27827	0.00016	0.000569	-
SE0239	mvaD	Diphosphomevalonate decarboxylase	CP000029.1:246043-247026	1.48493	0.000161	0.000572	UP
SE0323	SERP0323	Uncharacterized protein	CP000029.1:328084-328560	2.124566	0.000162	0.000575	UP
SE0155	folK	2-amino-4-hydroxy-6-hydroxymethyldihydropteridine p	CP000029.1:146637-147116	1.697157	0.00017	0.000601	UP
SE1432	SERP1432	DNA polymerase III, epsilon subunit, putative	CP000029.1:1493655-1494209	-0.18184	0.000171	0.000603	-
SE2172	SERP2172	acetyl-CoA synthetase, putative	CP000029.1:2202403-2203998	1.469183	0.000171	0.000603	UP
SE0514	SERP0514	conserved hypothetical protein	CP000029.1:500380-500814	-0.15681	0.000174	0.000613	-
SE0689	potD	spermidine preferential ABC transporter periplasmic bin	CP000029.1:684419-685492	-0.15212	0.000183	0.000643	-
SE0458	SERP0458	potassium uptake protein, TrkH family	CP000029.1:461861-463168	1.693597	0.000186	0.000654	UP
SE1435	SERP1435	23S rRNA (uracil-C(5))-methyltransferase RlmCD	CP000029.1:1496306-1497676	1.582278	0.000187	0.000656	UP
SE1043	SERP1043	GTPase Der	CP000029.1:1087991-1089301	1.460428	0.00019	0.000664	UP
SE2086	frp	NAD(P)H-flavin oxidoreductase	CP000029.1:2113358-2114020	-0.49786	0.000191	0.000668	-
SE0016	SERP0016	ABC transporter, permease protein	CP000029.1:11574-12770	1.636187	0.000193	0.000675	UP

SE2165	SERP2165	NADH dehydrogenase, NDH-2B	CP000029.1:2192892-2194253	-0.13532	0.000198	0.00069	-
SE0778	coaBC	phosphopantothenoylcysteine decarboxylase/phosphopa	CP000029.1:776624-777823	-0.30951	0.0002	0.000695	-
SE1286	SERP1286	Organic hydroperoxide resistance protein	CP000029.1:1330053-1330493	-1.22792	0.000206	0.000715	DOWN
SE2180	SERP2180	anaerobic C4-dicarboxylate membrane transporter	CP000029.1:2213467-2214765	1.423118	0.000211	0.000731	UP
SE0825	pyrH	uridylate kinase	CP000029.1:829440-830162	1.416172	0.000213	0.000736	UP
SE2251	SERP2251	arginine repressor	CP000029.1:2279187-2279648	-0.92675	0.000214	0.00074	-
SE1199	recJ	single-stranded-DNA-specific exonuclease RecJ	CP000029.1:1236096-1238369	1.493694	0.000215	0.00074	UP
SE1429	SERP1429	conserved hypothetical protein	CP000029.1:1490704-1491429	2.539602	0.000217	0.000746	UP
SE2188	SERP2188	siroheme synthase, precorrin 2 oxidase and ferrochelata	CP000029.1:2221197-2221808	1.785427	0.000224	0.000769	UP
SE1892	SERP1892	-	CP000029.1:1914135-1914410	1.637129	0.000227	0.000778	UP
SE2339	SERP2339	major facilitator family transporter	CP000029.1:2383754-2384995	2.136189	0.000229	0.000784	UP
SE0508	SERP0508	conserved hypothetical protein	CP000029.1:495263-496111	1.801999	0.000231	0.000791	UP
SE2160	SERP2160	-	CP000029.1:2189763-2190194	-0.20644	0.000232	0.000793	-
SE1366	hemG	protoporphyrinogen oxidase	CP000029.1:1422508-1423956	-0.1952	0.00026	0.000885	-
SE1470	SERP1470	conserved hypothetical protein	CP000029.1:1535812-1536648	1.562946	0.00026	0.000885	UP
SE1190	SERP1190	ATPase, AAA family	CP000029.1:1225301-1226572	-0.12358	0.000261	0.000888	-
SE1977	SERP1977	-	CP000029.1:1995122-1995487	-0.5671	0.000265	0.0009	-
SE0763	SERP0763	Pseudouridine synthase	CP000029.1:760946-761863	1.4806	0.00027	0.00091	UP
SE1234	hemC	porphobilinogen deaminase	CP000029.1:1269281-1270207	1.452952	0.00027	0.00091	UP
SE1947	tcaB	drug resistance transporter, Bcr/CflA family	CP000029.1:1961721-1962932	1.444707	0.00027	0.00091	UP
SE2547	SERP2547	conserved hypothetical protein	CP000029.1:2605887-2606702	-0.33301	0.00027	0.00091	-
SE1313	SERP1313	Polysaccharide biosynthesis protein, putative	CP000029.1:1362053-1363714	1.42845	0.000284	0.000957	UP
SE0230	SERP0230	conserved hypothetical protein	CP000029.1:237807-238178	1.660242	0.000291	0.00098	UP
SE2066	SERP2066	-	CP000029.1:2091456-2091869	-0.12373	0.000296	0.000994	-
SE0774	SERP0774	conserved hypothetical protein	CP000029.1:772813-773220	1.518603	0.000299	0.001001	UP
SE0414	SERP0414	glycosyl transferase, group 4 family protein	CP000029.1:414923-416002	1.432757	0.000301	0.001007	UP
SE0473	SERP0473	-	CP000029.1:473071-473208	-0.99092	0.000303	0.001013	-
SE2194	gpxA-2	Glutathione peroxidase	CP000029.1:2227609-2228085	-0.17363	0.000309	0.001032	-
SE1181	SERP1181	conserved hypothetical protein	CP000029.1:1214218-1214478	-0.12942	0.000311	0.001037	-
SE0335	SERP0335	Cytokinin riboside 5'-monophosphate phosphoribohydro	CP000029.1:337593-338159	-0.15433	0.000316	0.00105	-
SE2225	SERP2225	HTH-type transcriptional regulator NmtR	CP000029.1:2259780-2260124	-0.10812	0.000333	0.001106	-
SE1413	SERP1413	Uncharacterized protein SAOUHSC_02013	CP000029.1:1477715-1478233	-0.10392	0.000335	0.001112	-
SE0456	SERP0456	-	CP000029.1:460587-460757	3.709591	0.00034	0.001124	UP
SE1783	SERP1783	-	CP000029.1:1828897-1829136	-0.10934	0.000342	0.00113	-
SE2206	SERP2206	DNA-binding response regulator	CP000029.1:2241347-2242012	-0.14572	0.000344	0.001134	-
SE1081	argR	arginine repressor	CP000029.1:1127580-1128032	-0.19391	0.000345	0.001136	-
SE0441	gapR	gapA transcriptional regulator CggR	CP000029.1:445408-446421	-0.10198	0.000359	0.001181	-
SE0497	sufD	FeS assembly protein SufD	CP000029.1:486407-487714	1.373538	0.000368	0.001207	UP
SE1177	SERP1177	peptidase, U32 family	CP000029.1:1211475-1212398	1.561322	0.000376	0.001232	UP
SE2346	gldA	mitochondrial glycerol dehydrogenase Gld1	CP000029.1:2391590-2392693	-0.09827	0.000377	0.001235	-
SE1307	trmB	tRNA (guanine-N(7)-)-methyltransferase	CP000029.1:1356510-1357157	2.083068	0.000379	0.001238	UP
SE1042	gpsA	Glycerol-3-phosphate dehydrogenase [NAD(P)+]	CP000029.1:1086972-1087970	1.389415	0.000409	0.001334	UP
SE1757	SERP1757	phosphatase family protein	CP000029.1:1793365-1794096	-0.27958	0.000409	0.001334	-
SE1883	SERP1883	COG3296 family membrane protein	CP000029.1:1908567-1909061	1.37948	0.000424	0.001379	UP
SE2132	SERP2132	copper-ion-binding protein	CP000029.1:2158331-2158537	-0.40593	0.000433	0.001407	-
SE1101	SERP1101	Probable glycine dehydrogenase (decarboxylating) subu	CP000029.1:1144334-1145680	1.367076	0.000437	0.001417	UP
SE1097	SERP1097	lipoate-protein ligase A, putative	CP000029.1:1140565-1141395	-0.22568	0.000444	0.001439	-
SE1311	SERP1311	-	CP000029.1:1360918-1361346	-0.16515	0.000445	0.00144	-
SE1045	cmk	cytidylate kinase	CP000029.1:1091179-1091826	-0.14341	0.00047	0.001519	-
SE0893	SERP0893	-	CP000029.1:904340-905212	-0.20315	0.000477	0.001536	-
SE0727	SERP0727	MutS2 family protein	CP000029.1:724869-727217	1.466486	0.000483	0.001551	UP
SE2101	SERP2101	D-ribose pyranase	CP000029.1:2125942-2126346	1.508406	0.000482	0.001551	UP
SE0585	SERP0585	conserved hypothetical protein	CP000029.1:578981-579328	-0.63625	0.000484	0.001552	-
SE1229	SERP1229	3-methyl-adenine DNA glycosylase I, constitutive	CP000029.1:1264212-1264772	-0.16924	0.000488	0.001565	-
SE1066	SERP1066	Ribonuclease Z	CP000029.1:1110268-1111188	-0.16403	0.000496	0.001587	-
SE1725	prfA	Peptide chain release factor 1	CP000029.1:1762086-1763162	1.628503	0.000505	0.001614	UP
SE0974	SERP0974	Acylphosphatase	CP000029.1:991427-991696	-0.23019	0.00054	0.001722	-
SE2126	feoB	ferrous iron transport protein B	CP000029.1:2149737-2151731	-0.22815	0.000547	0.00174	-
SE1371	SERP1371	ABC transporter, permease protein EscB	CP000029.1:1428144-1429367	1.361639	0.000553	0.001758	UP
SE1909	SERP1909	Escherichia coli K-12	CP000029.1:1928396-1929985	1.351592	0.000556	0.001765	UP
SE0067	xpt	Xanthine phosphoribosyltransferase	CP000029.1:55201-55779	1.408504	0.000569	0.001803	UP
SE0270	SERP0270	-	CP000029.1:276794-277303	-0.07758	0.000574	0.001818	-
SE0261	SERP0261	-	CP000029.1:268675-269130	-0.95246	0.000577	0.001823	-
SE2538	rplI	50S ribosomal protein L9	CP000029.1:2595972-2596418	1.455087	0.000586	0.001848	UP
SE0695	SERP0695	-	CP000029.1:691405-691596	1.532659	0.000588	0.001851	UP
SE1092	argC	N-acetyl-gamma-glutamyl-phosphate reductase	CP000029.1:1136791-1137816	-0.73812	0.000588	0.001851	-
SE0714	SERP0714	methyltransferase, putative	CP000029.1:709691-710233	-0.2599	0.000591	0.001855	-
SE0613	SERP0613	5'-nucleotidase domain/Ser/Thr protein phosphatase dor	CP000029.1:608008-609513	-0.08203	0.000602	0.001887	-
SE0240	SERP0240	mevalonate kinase, putative	CP000029.1:247039-248115	1.362802	0.000604	0.001891	UP
SE1154	SERP1154	DNA polymerase III, delta subunit	CP000029.1:1191827-1192801	1.908597	0.000611	0.001907	UP
SE1205	ruvB	Holliday junction DNA helicase RuvB	CP000029.1:1244207-1245211	1.381068	0.00061	0.001907	UP
SE0494	SERP0494	conserved hypothetical protein	CP000029.1:484112-484303	-0.13833	0.000636	0.001982	-
SE0165	clpC	ATP-dependent Clp protease ATP-binding subunit Clp(	CP000029.1:169002-171455	-0.0721	0.00064	0.001993	-
SE0334	SERP0334	Uncharacterized protein	CP000029.1:336746-337294	1.735738	0.000648	0.002015	UP
SE2127	SERP2127	FeoA family protein	CP000029.1:2151737-2151964	-3.31262	0.000667	0.002071	DOWN
SE0912	tkt	transketolase	CP000029.1:920892-922880	1.33366	0.000685	0.002122	UP

SE1421	SERP1421	YihY family protein	CP000029.1:1483504-1484682	1.353354	0.00069	0.002137	UP
SE1288	serA	D-3-phosphoglycerate dehydrogenase	CP000029.1:1331773-1333368	-0.09517	0.000716	0.002213	-
SE0884	SERP0884	-	CP000029.1:897344-897541	1.916011	0.00072	0.002223	UP
SE0297	SERP0297	Transport permease protein	CP000029.1:299351-300163	1.374883	0.000753	0.002321	UP
SE1192	aspS	aspartyl-tRNA synthetase	CP000029.1:1228098-1229864	1.375689	0.000758	0.002334	UP
SE0889	SERP0889	DNA-binding response regulator	CP000029.1:901943-902545	1.657385	0.000771	0.002371	UP
SE0917	sbcD	exonuclease SbcD, putative	CP000029.1:925453-926577	-0.189	0.000785	0.002408	-
SE1403	SERP1403	Putative multidrug export ATP-binding/permease protei	CP000029.1:1467814-1469550	1.440258	0.000797	0.002441	UP
SE1376	SERP1376	protein export protein prsA	CP000029.1:1432032-1433009	1.490225	0.000807	0.002468	UP
SE0161	nupC	Uncharacterized protein	CP000029.1:165575-166789	1.359548	0.000815	0.002486	UP
SE1164	SERP1164	Uncharacterized protein	CP000029.1:1199677-1200777	1.421731	0.000815	0.002486	UP
SE0527	SERP0527	pyridine nucleotide-disulphide oxidoreductase	CP000029.1:510733-511941	1.324803	0.000834	0.002541	UP
SERP_tRN	SERP_tRNA	-	CP000029.1:98388-2592415	1.325358	0.000849	0.002581	UP
SE2372	SERP2372	Peptide ABC transporter, peptide-binding protein, putat	CP000029.1:2419711-2421309	-1.25383	0.000858	0.002605	DOWN
SE0182	SERP0182	Uncharacterized protein	CP000029.1:185105-185713	1.46759	0.000865	0.002623	UP
SE0666	SERP0666	-	CP000029.1:661356-662648	1.341559	0.000874	0.002645	UP
SE0946	femA	-	CP000029.1:961515-962768	1.386573	0.000876	0.002645	UP
SE1717	SERP1717	UDP-N-acetylglucosamine 2-epimerase	CP000029.1:1755916-1757061	-0.0537	0.000875	0.002645	-
SE0144	SERP0144	S4 domain protein	CP000029.1:137099-137362	2.594051	0.000878	0.002648	UP
SE1678	rsbW	Serine-protein kinase RsbW	CP000029.1:1723550-1724029	1.412613	0.000955	0.002877	UP
SE1765	SERP1765	Iron-sulfur cluster carrier protein	CP000029.1:1808640-1809707	1.418663	0.000957	0.002877	UP
SE1112	glk	glucokinase	CP000029.1:1152162-1153148	1.325445	0.001021	0.003066	UP
SE2283	SERP2283	Phosphonates ABC transporter, permease protein CC03	CP000029.1:2323914-2324729	1.464431	0.001029	0.003087	UP
SE1756	SERP1756	Cation efflux family protein, putative	CP000029.1:1792151-1793104	2.277593	0.001041	0.003118	UP
SE0557	SERP0557	conserved hypothetical protein	CP000029.1:547948-548349	1.359316	0.001084	0.003241	UP
SE2079	SERP2079	Putative membrane protein	CP000029.1:2105295-2106344	-0.26154	0.001086	0.003241	-
SE0188	fusA	Elongation factor G	CP000029.1:194988-197069	1.302518	0.001121	0.003343	UP
SE0363	SERP0363	Uncharacterized protein	CP000029.1:363278-364384	1.312228	0.001123	0.003344	UP
SE0904	rpmG-2	50S ribosomal protein L33 1	CP000029.1:916157-916306	-0.04724	0.001136	0.003378	-
SE2345	SERP2345	dihydroxyacetone kinase family protein	CP000029.1:2390612-2391574	-0.0355	0.001145	0.003401	-
SE1834	SERP1834	-	CP000029.1:1862776-1863174	-0.03614	0.00116	0.00344	-
SE2196	SERP2196	Transcriptional regulator, MarR family	CP000029.1:2229458-2229898	-0.2377	0.001168	0.003457	-
SE0191	SERP0191	acetyltransferase, GNAT family	CP000029.1:200341-200850	-0.40595	0.001179	0.003483	-
SE1167	SERP1167	putative membrane protein	CP000029.1:1202210-1203454	-0.03883	0.00118	0.003483	-
SE1103	aroK	Shikimate kinase	CP000029.1:1147009-1147506	1.303088	0.001194	0.003519	UP
SE1944	SERP1944	drug resistance transporter, EmrB/QacA family	CP000029.1:1958356-1960332	1.340133	0.001204	0.003544	UP
SE1780	SERP1780	transporter, putative	CP000029.1:1825021-1826202	-0.09431	0.001233	0.003625	-
SE1852	moaD	molybdopterin converting factor, subunit 1	CP000029.1:1881539-1881772	-0.33011	0.001245	0.003657	-
SE1191	SERP1191	Uncharacterized protein	CP000029.1:1226766-1227542	1.38857	0.00125	0.003666	UP
SE0524	SERP0524	pyridine nucleotide-disulphide oxidoreductase	CP000029.1:508478-509542	-0.06771	0.001266	0.003706	-
SE0805	rimM	Ribosome maturation factor RimM	CP000029.1:806687-807190	-3.16056	0.001268	0.003708	DOWN
SE0204	SERP0204	tRNA-specific adenosine deaminase	CP000029.1:213774-214280	-0.32297	0.001292	0.003774	-
SE1039	SERP1039	Menaquinone biosynthesis methyltransferase, putative	CP000029.1:1084763-1085488	1.317881	0.001323	0.003858	UP
SE0339	bacA	bacitracin resistance protein	CP000029.1:340246-341118	1.385083	0.001361	0.003958	UP
SE0883	SERP0883	Uncharacterized protein	CP000029.1:896005-897030	-0.05547	0.001361	0.003958	-
SE1152	SERP1152	Elongation factor 4	CP000029.1:1189342-1191165	1.469776	0.001368	0.003972	UP
SE1762	glmM	Phosphoglucosamine mutase	CP000029.1:1799474-1800829	1.322687	0.001382	0.004009	UP
SE1388	SERP1388	-	CP000029.1:1445896-1446300	1.41627	0.001391	0.004028	UP
SE1367	hemH	Ferrochelatase	CP000029.1:1424049-1424972	-0.18256	0.001402	0.004054	-
SE2138	SERP2138	-	CP000029.1:2164774-2165481	1.321042	0.001492	0.00431	UP
SE0918	sbcC	exonuclease, putative	CP000029.1:926581-929610	1.355819	0.001523	0.004391	UP
SE1148	dnaK	chaperone protein dnaK	CP000029.1:1184476-1186305	-0.01737	0.001571	0.004519	-
SE1180	SERP1180	conserved hypothetical protein TIGR00250	CP000029.1:1213788-1214216	-0.05367	0.00157	0.004519	-
SE0032	SERP0032	acetyl-CoA acetyltransferase	CP000029.1:26288-27472	1.377186	0.001732	0.004967	UP
SE1423	vraS	sensor histidine kinase	CP000029.1:1485358-1486404	1.377186	0.001732	0.004967	UP
SE1083	xseB	Exodeoxyribonuclease 7 small subunit	CP000029.1:1129206-1129436	1.968465	0.001789	0.005124	UP
SE0819	hslV	ATP-dependent protease hslV	CP000029.1:824310-824852	1.385244	0.001806	0.005165	UP
SE1801	SERP1801	cobalt transport protein	CP000029.1:1845708-1846514	1.603101	0.001855	0.0053	UP
SE1209	rpmA	50S ribosomal protein L27	CP000029.1:1247745-1248029	-0.02515	0.00186	0.005305	-
SE1390	SERP1390	-	CP000029.1:1446659-1447258	-0.00778	0.001884	0.005361	-
SE2244	SERP2244	conserved hypothetical protein	CP000029.1:2271458-2272624	-0.12672	0.001884	0.005361	-
SE1407	SERP1407	yfhP protein	CP000029.1:1471665-1472639	1.2895	0.001905	0.005412	UP
SE1063	SERP1063	lolS protein	CP000029.1:1107353-1108261	1.297505	0.001939	0.005501	UP
SE0036	SERP0036	cystathionine beta-lyase	CP000029.1:32490-33665	-0.44023	0.001952	0.005531	-
SE0515	SERP0515	Acid sugar phosphatase	CP000029.1:500814-501593	-0.01187	0.001958	0.005539	-
SE2296	icaC	-	CP000029.1:2336580-2337647	-0.5473	0.001974	0.00558	-
SE1041	hup	DNA-binding protein HU, putative	CP000029.1:1086529-1086801	-0.00752	0.001985	0.005602	-
SE1248	SERP1248	DNA replication protein DnaB	CP000029.1:1284802-1286172	-0.05789	0.001993	0.005618	-
SE1396	SERP1396	conserved hypothetical protein	CP000029.1:1452286-1453344	1.479053	0.001997	0.005622	UP
SE2542	SERP2542	conserved hypothetical protein	CP000029.1:2600843-2601172	-0.67539	0.002064	0.005802	-
SE0784	SERP0784	Probable dual-specificity RNA methyltransferase RlmN	CP000029.1:783482-784576	1.325865	0.002121	0.005953	UP
SE1893	SERP1893	transcription antiterminator LytR	CP000029.1:1915044-1915994	1.699971	0.002146	0.006017	UP
SE0924	SERP0924	Glycerol-3-phosphate acyltransferase	CP000029.1:936237-936845	-0.08401	0.002173	0.006084	-
SE1763	SERP1763	conserved hypothetical protein	CP000029.1:1800854-1801789	1.370387	0.002236	0.006251	UP
SE0612	SERP0612	sodium transporter family protein	CP000029.1:606511-607869	1.314907	0.002257	0.006303	UP
SE2289	SERP2289	Major facilitator family transporter	CP000029.1:2330888-2332063	-0.01663	0.002352	0.00656	-

SE1085	nusB	N utilization substance protein B	CP000029.1:1130781-1131170	-0.15409	0.002396	0.006672	-
SE0971	SERP0971	-	CP000029.1:990377-990589	Inf (+)	0.002421	0.006734	UP
SE1065	proC	pyrroline-5-carboxylate reductase	CP000029.1:1109403-1110218	-0.08979	0.002427	0.006741	-
SE1040	SERP1040	-	CP000029.1:1085481-1086059	-0.04103	0.00244	0.00677	-
SE0811	SERP0811	Ribosome biogenesis GTPase A	CP000029.1:814088-814951	-0.12906	0.00247	0.006844	-
SE1708	atpC	ATP synthase epsilon chain	CP000029.1:1748909-1749313	0.008906	0.00248	0.00686	-
SE0425	SERP0425	-	CP000029.1:427742-427834	-2.45006	0.002504	0.006915	DOWN
SE1915	SERP1915	-	CP000029.1:1935577-1936164	-0.0353	0.002506	0.006915	-
SE0537	mnhB	monovalent cation/proton antiporter, MnhA/PhaA famil	CP000029.1:520098-520526	1.342319	0.002564	0.007067	UP
SE1831	rplC	ribosomal protein L3	CP000029.1:1861422-1862084	1.255809	0.002575	0.007086	UP
SE1291	SERP1291	1-acyl-sn-glycerol-3-phosphate acyltransferases domain	CP000029.1:1336350-1336973	1.368889	0.00258	0.007092	UP
SE0291	sitB	Iron (Chelated) ABC transporter, permease protein, puta	CP000029.1:293956-294792	1.254491	0.002606	0.007155	UP
SE0567	fabH	3-oxoacyl-(acyl-carrier-protein) synthase III	CP000029.1:560502-561443	1.422498	0.00267	0.007321	UP
SE1178	SERP1178	O-methyltransferase family protein	CP000029.1:1212401-1213036	1.409279	0.002712	0.007415	UP
SE1955	mqo-1	malate:quinone-oxidoreductase	CP000029.1:1970556-1972034	1.264284	0.002711	0.007415	UP
SE0820	hslU	ATP-dependent hsl protease, ATP-binding subunit hslU	CP000029.1:824921-826324	1.264226	0.002718	0.007423	UP
SE0469	SERP0469	-	CP000029.1:470423-471169	-0.02938	0.002729	0.007443	-
SE0071	SERP0071	Abi-like family protein	CP000029.1:60500-61405	1.290965	0.0028	0.007627	UP
SE0326	SERP0326	-	CP000029.1:330790-331008	-1.31278	0.002853	0.00775	DOWN
SE2303	hisH	Imidazole glycerol phosphate synthase subunit HisH	CP000029.1:2343293-2343871	2.867161	0.002853	0.00775	UP
SE1779	SERP1779	Dictyostelium discoideum	CP000029.1:1823274-1825031	0.006831	0.0029	0.007867	-
SE1840	SERP1840	-	CP000029.1:1868981-1869889	1.289952	0.002903	0.007867	UP
SE1195	dtd	D-aminoacyl-tRNA deacylase	CP000029.1:1232385-1232837	1.365793	0.002909	0.007873	UP
SE2404	SERP2404	iron compound ABC transporter, iron compound-bindin	CP000029.1:2464409-2465383	-0.11954	0.002922	0.007898	-
SE1253	fpg	Formamidopyrimidine-DNA glycosylase	CP000029.1:1289659-1290531	1.317421	0.002964	0.008	UP
SE0580	pepF	Oligoendopeptidase F	CP000029.1:574567-576375	0.019757	0.002969	0.008003	-
SE0359	fruA	PTS system fructose-specific EIIABC component	CP000029.1:356937-358889	1.274478	0.002991	0.008055	UP
SE2264	SERP2264	-	CP000029.1:2298118-2300148	0.013204	0.003029	0.008145	-
SE1210	SERP1210	conserved hypothetical protein	CP000029.1:1248041-1248361	1.262713	0.003045	0.008177	UP
SE0397	nrdE	Ribonucleoside-diphosphate reductase	CP000029.1:398490-400595	0.022323	0.003058	0.008203	-
SE0916	SERP0916	ccdC protein	CP000029.1:924878-925345	1.53266	0.003062	0.008203	UP
SE0845	SERP0845	conserved hypothetical protein	CP000029.1:855756-857027	1.835093	0.003121	0.00835	UP
SE0835	SERP0835	ribosomal protein L7A family	CP000029.1:842504-842821	1.331027	0.003176	0.008487	UP
SE1365	SERP1365	RB binding protein 9, serine hydrolase	CP000029.1:1421818-1422372	-0.84884	0.003182	0.008493	-
SE0179	rplA	50S ribosomal protein L1	CP000029.1:183037-183732	1.243554	0.003205	0.00852	UP
SE0681	pdhB	pyruvate dehydrogenase complex E1 component, beta s	CP000029.1:676624-677601	1.239124	0.003203	0.00852	UP
SE0704	pyc	pyruvate carboxylase	CP000029.1:699164-702607	1.24008	0.003202	0.00852	UP
SE0536	mnhC	monovalent cation/proton antiporter, MnhC/PhaC famil	CP000029.1:519751-520098	1.442682	0.00324	0.008603	UP
SE2057	gntP	Gluconate permease, putative	CP000029.1:2081933-2083291	1.241593	0.003284	0.00871	UP
SE0821	codY	GTP-sensing transcriptional pleiotropic repressor CodY	CP000029.1:826348-827118	1.273679	0.003356	0.008889	UP
SE0950	SERP0950	oligopeptide ABC transporter, ATP-binding protein	CP000029.1:967035-967730	1.646537	0.00337	0.008909	UP
SE2042	SERP2042	-	CP000029.1:2063406-2063873	-0.20364	0.003372	0.008909	-
SE2110	SERP2110	membrane protein, putative	CP000029.1:2133872-2134720	2.331087	0.003395	0.008958	UP
SE0730	sdhC	succinate dehydrogenase, cytochrome b558 subunit	CP000029.1:730038-730652	0.031648	0.003438	0.00906	-
SE0771	pyrE	orotate phosphoribosyltransferase	CP000029.1:771429-772040	1.242214	0.003643	0.009588	UP
SE0616	SERP0616	Lipoate--protein ligase 1	CP000029.1:610614-611600	0.011632	0.003661	0.009619	-
SE1059	SERP1059	conserved hypothetical protein	CP000029.1:1104667-1105191	-0.61544	0.003663	0.009619	-
SE1064	SERP1064	oxidoreductase, short-chain dehydrogenase/reductase fa	CP000029.1:1108484-1109242	-0.15595	0.00368	0.00965	-
SE0301	SERP0301	-	CP000029.1:303316-303426	1.939828	0.003725	0.009757	UP
SE0192	SERP0192	ornithine cyclodeaminase/mu-crystallin family protein	CP000029.1:201102-202052	-0.37151	0.003775	0.009872	-
SE0214	SERP0214	conserved hypothetical protein	CP000029.1:225111-225473	-0.82102	0.003778	0.009872	-
SE0428	hprK	HPr kinase/phosphorylase	CP000029.1:432998-433930	-0.02096	0.00384	0.010008	-
SE1211	rplU	50S ribosomal protein L21	CP000029.1:1248366-1248674	1.247759	0.00384	0.010008	UP
SE1138	SERP1138	PhoH family protein	CP000029.1:1175368-1176315	0.024866	0.003857	0.01004	-
SE0384	recQ-1	ATP-dependent DNA helicase RecQ	CP000029.1:383163-384941	1.430559	0.003877	0.01008	UP
SE1134	era	GTP-binding protein Era	CP000029.1:1173239-1174138	1.2459	0.004008	0.010408	UP
SE0206	SERP0206	azoreductase	CP000029.1:215331-215897	1.292102	0.004027	0.010443	UP
SE2010	SERP2010	-	CP000029.1:2026367-2027839	-0.61239	0.004053	0.010498	-
SE0861	SERP0861	conserved hypothetical protein	CP000029.1:873062-873427	1.501496	0.004058	0.010498	UP
SE2064	SERP2064	-	CP000029.1:2089454-2090371	2.067974	0.004325	0.011174	UP
SE1015	SERP1015	conserved hypothetical protein	CP000029.1:1059841-1060977	1.485926	0.004337	0.01118	UP
SE1677	rpoF	RNA polymerase sigma factor	CP000029.1:1722805-1723575	1.227898	0.004336	0.01118	UP
SE1914	SERP1914	Na+/H+ antiporter family protein	CP000029.1:1933970-1935271	1.261922	0.004469	0.011506	UP
SE2154	budA	alpha-acetolactate decarboxylase	CP000029.1:2180841-2181545	0.046533	0.004483	0.011527	-
SE1089	argD	Acetylornithine aminotransferase	CP000029.1:1133703-1134830	-0.35045	0.004512	0.011588	-
SE0831	SERP0831	DNA polymerase III, alpha subunit, Gram-positive type	CP000029.1:836002-840312	1.226103	0.004663	0.011961	UP
SE0665	SERP0665	-	CP000029.1:661070-661204	-0.01717	0.004753	0.012176	-
SE1016	SERP1016	conserved hypothetical protein	CP000029.1:1061620-1061958	1.32422	0.004777	0.012225	UP
SE0748	murD	UDP-N-acetylmuramoylalanine--D-glutamate ligase	CP000029.1:745384-746733	1.222235	0.004786	0.012231	UP
SE0476	SERP0476	Uncharacterized protein	CP000029.1:474014-474601	1.915977	0.004809	0.012276	UP
SE0418	SERP0418	comF operon protein 3	CP000029.1:419114-419788	-0.1912	0.004869	0.012414	-
SE2142	SERP2142	Uncharacterized protein	CP000029.1:2169022-2170401	1.212298	0.004877	0.012418	UP
SE0433	SERP0433	conserved hypothetical protein	CP000029.1:437908-438795	1.415726	0.004962	0.012619	UP
SE1014	SERP1014	Drosophila melanogaster	CP000029.1:1059035-1059673	1.394747	0.005	0.012702	UP
SE1660	SERP1660	MutS domain V protein	CP000029.1:1698229-1699845	-0.0072	0.005085	0.012902	-
SE1374	SERP1374	-	CP000029.1:1430732-1431103	0.048198	0.005159	0.013075	-

SE1198	apt	adenine phosphoribosyltransferase	CP000029.1:1235559-1236077	1.317051	0.005204	0.013167	UP
SE2028	SERP2028	Amino acid ABC transporter, permease protein, putative	CP000029.1:2048369-2049064	1.211662	0.005208	0.013167	UP
SE0331	SERP0331	-	CP000029.1:334642-335331	-0.66075	0.005264	0.013292	-
SE0120	tmk	Thymidylate kinase	CP000029.1:113038-113649	0.015712	0.005273	0.013299	-
SE0387	hisC	histidinol-phosphate aminotransferase	CP000029.1:387825-388880	1.223639	0.005319	0.013399	UP
SE2075	SERP2075	Alkaline phosphatase	CP000029.1:2100185-2100799	-0.03821	0.005486	0.013804	-
SE0049	SERP0049	conserved hypothetical protein	CP000029.1:42464-42718	0.06128	0.005521	0.013876	-
SE0870	miaA	tRNA dimethylallyltransferase	CP000029.1:885127-886125	0.058195	0.005539	0.013905	-
SE1807	rpsM	30S ribosomal protein S13	CP000029.1:1850319-1850684	0.062704	0.005552	0.01392	-
SE0407	SERP0407	-	CP000029.1:407931-408821	-0.06631	0.005633	0.014106	-
SE0782	fmt	Methionyl-tRNA formyltransferase	CP000029.1:781243-782175	1.376465	0.005713	0.01429	UP
SERP_Se1	rrsF	Uncharacterized protein	CP000029.1:1806632-1808185	-1.99067	0.005721	0.014291	DOWN
SE0448	secG	preprotein translocase, SecG subunit	CP000029.1:453415-453648	1.325207	0.005744	0.014332	UP
SE1159	SERP1159	Ribosomal silencing factor RsfS	CP000029.1:1197062-1197415	-0.25768	0.005757	0.014349	-
SE1079	lpdA	dihydrolipoamide dehydrogenase	CP000029.1:1124207-1125628	1.281029	0.005792	0.014419	UP
SE0925	parE	DNA topoisomerase 4 subunit B	CP000029.1:937049-939049	1.29686	0.005869	0.014594	UP
SE2095	sdhB	L-serine dehydratase, iron-sulfur-dependent, beta subun	CP000029.1:2119745-2120428	0.038244	0.005962	0.014806	-
SE0039	SERP0039	Uncharacterized protein	CP000029.1:36497-37372	1.75913	0.006	0.014883	UP
SE0199	SERP0199	haloacid dehalogenase/epoxide hydrolase family protein	CP000029.1:209189-209884	-0.21871	0.006041	0.014969	-
SE0444	tpiA	Triosephosphate isomerase	CP000029.1:448998-449759	0.070779	0.006062	0.015002	-
SE1709	atpD	ATP synthase F1, beta subunit	CP000029.1:1749333-1750745	0.070659	0.006091	0.015056	-
SE2129	SERP2129	oxidoreductase, short chain dehydrogenase/reductase fa	CP000029.1:2154300-2155169	0.067629	0.006258	0.01545	-
SE0720	SERP0720	Uncharacterized protein	CP000029.1:716482-717222	-0.03707	0.006424	0.015842	-
SE0639	SERP0639	-	CP000029.1:632900-633370	1.231098	0.006474	0.015946	UP
SE0224	SERP0224	-	CP000029.1:233685-233864	0.074517	0.006504	0.016002	-
SE2065	SERP2065	Uncharacterized protein	CP000029.1:2090491-2091192	-0.02923	0.006528	0.016043	-
SE2027	lrgB	Antiholin-like protein LrgB	CP000029.1:2047486-2048187	1.191426	0.006579	0.016149	UP
SE1499	SERP1499	putative sulfurtransferase YeeD	CP000029.1:1566662-1566886	2.218639	0.006607	0.016161	UP
SE1721	SERP1721	low molecular weight protein-tyrosine-phosphatase	CP000029.1:1759641-1760060	2.218639	0.006607	0.016161	UP
SE2414	SERP2414	-	CP000029.1:2476275-2476634	-0.88396	0.006607	0.016161	-
SE0082	SERP0082	-	CP000029.1:69325-69438	-1.27097	0.00663	0.016199	DOWN
SE0554	rexB	ATP-dependent nuclease, subunit B	CP000029.1:539364-542843	1.214343	0.006664	0.01626	UP
SE0965	dapA	dihydrodipicolinate synthase	CP000029.1:983825-984709	0.071693	0.00668	0.01626	-
SE1898	SERP1898	conserved hypothetical protein	CP000029.1:1919607-1920659	0.076581	0.006679	0.01626	-
SE2036	SERP2036	major facilitator family transporter	CP000029.1:2057494-2058672	1.262485	0.006686	0.01626	UP
SE2044	SERP2044	-	CP000029.1:2065150-2065338	0.058943	0.006858	0.01666	-
SE0415	SERP0415	conserved hypothetical protein TIGR00257	CP000029.1:416063-416704	-0.07153	0.006918	0.016787	-
SE0823	rpsB	30S ribosomal protein S2	CP000029.1:827481-828269	1.190105	0.006946	0.016834	UP
SE0498	sufS	cysteine desulfurase SufS	CP000029.1:487782-489023	1.189279	0.007005	0.016939	UP
SE1176	SERP1176	peptidase, U32 family	CP000029.1:1210189-1211457	1.247257	0.007005	0.016939	UP
SE0312	SERP0312	DNA-binding response regulator	CP000029.1:315484-316158	0.024331	0.007173	0.017303	-
SE1163	aroE	Shikimate dehydrogenase (NADP(+))	CP000029.1:1198852-1199661	1.215324	0.007173	0.017303	UP
SE2304	hisB	imidazoleglycerol-phosphate dehydratase	CP000029.1:2343868-2344446	2.331017	0.00718	0.017303	UP
SE1877	SERP1877	-	CP000029.1:1900760-1901083	0.030511	0.007223	0.017387	-
SE1023	SERP1023	DNA replication protein DnaD	CP000029.1:1067256-1067942	1.263443	0.007252	0.017435	UP
SE1100	SERP1100	Probable glycine dehydrogenase (decarboxylating) subu	CP000029.1:1142833-1144341	1.196177	0.007423	0.017828	UP
SE1942	SERP1942	-	CP000029.1:1957543-1958175	-0.33668	0.007441	0.017851	-
SE0485	gcvH	glycine cleavage system H protein	CP000029.1:479395-479775	0.009099	0.007588	0.018182	-
SE1768	SERP1768	multidrug transporter, putative	CP000029.1:1812225-1813562	1.2969	0.007719	0.018475	UP
SE2023	SERP2023	drug resistance transporter, EmrB/QacA family protein	CP000029.1:2042550-2043950	1.297342	0.007904	0.018897	UP
SE0614	SERP0614	-	CP000029.1:609616-610218	1.254214	0.007933	0.018943	UP
SE0800	SERP0800	Chromosome partition protein Smc	CP000029.1:799454-803023	1.203122	0.008114	0.019354	UP
SE2344	SERP2344	Escherichia coli K-12	CP000029.1:2389982-2390557	0.09067	0.00833	0.019848	-
SE1125	SERP1125	GTP cyclohydrolase 1 type 2 homolog	CP000029.1:1163861-1164961	1.218371	0.008431	0.020064	UP
SE0146	SERP0146	S1 RNA binding domain protein	CP000029.1:137881-138282	-0.03646	0.008496	0.020133	-
SE0461	SERP0461	-	CP000029.1:463958-464374	1.295811	0.008491	0.020133	UP
SE1171	SERP1171	urea amidolyase-related protein	CP000029.1:1206034-1207038	-0.03252	0.008479	0.020133	-
SE1182	alaS	Alanine--tRNA ligase	CP000029.1:1214542-1217172	0.082945	0.008498	0.020133	-
SE1093	efp	translation elongation factor P	CP000029.1:1137966-1138523	1.180899	0.008624	0.02041	UP
SE0008	SERP0008	-	CP000029.1:6522-6683	Inf (+)	0.008941	0.021137	UP
SE1385	SERP1385	sensor histidine kinase	CP000029.1:1441849-1442982	1.594064	0.009021	0.021302	UP
SE0472	SERP0472	-	CP000029.1:472490-472678	-0.14452	0.009059	0.021367	-
SE0512	SERP0512	conserved hypothetical protein	CP000029.1:499546-499935	0.084694	0.009269	0.021838	-
SE0471	SERP0471	-	CP000029.1:471746-472300	-0.12974	0.009425	0.022182	-
SE1839	gdh	glucose 1-dehydrogenase	CP000029.1:1867995-1868786	0.099888	0.009482	0.022291	-
SE1227	folC	FolC bifunctional protein	CP000029.1:1259963-1261228	1.182152	0.009584	0.022505	UP
SE0022	SERP0022	-	CP000029.1:19043-19423	3.9155	0.009611	0.022519	UP
SE0911	SERP0911	UPF0291 protein SP_1473	CP000029.1:920424-920660	4.008625	0.009611	0.022519	UP
SE1123	nfo	Probable endonuclease 4	CP000029.1:1161580-1162470	1.19489	0.009624	0.022524	UP
SE1875	ureD	Urease accessory protein UreH	CP000029.1:1899070-1899906	0.097351	0.009758	0.022812	-
SE1206	ruvA	Holliday junction DNA helicase RuvA	CP000029.1:1245225-1245827	1.203587	0.009887	0.023088	UP
SE0169	cysE	serine O-acetyltransferase	CP000029.1:176657-177298	0.05649	0.009932	0.023168	-
SE0608	SERP0608	-	CP000029.1:601599-601844	2.289189	0.010068	0.023459	UP
SE0814	sucD	Succinyl-CoA ligase [ADP-forming] subunit alpha	CP000029.1:817022-817930	1.162182	0.010147	0.023618	UP
SE1715	atpB	ATP synthase F0, A subunit	CP000029.1:1754659-1755387	0.105974	0.010234	0.023793	-
SE0679	SERP0679	lipoprotein, putative	CP000029.1:674711-675337	-1.21335	0.010399	0.024124	DOWN

SE1058	scpA	Segregation and condensation protein A	CP000029.1:1103838-1104569	0.018845	0.010395	0.024124	-
SE0280	SERP0280	-	CP000029.1:283013-283219	-0.33379	0.010447	0.024182	-
SE1165	SERP1165	Uncharacterized protein	CP000029.1:1200778-1201305	1.632032	0.010447	0.024182	UP
SE1683	alr	alanine racemase	CP000029.1:1726419-1727567	1.189174	0.010675	0.024683	UP
SE2242	ipdC	Branched-chain alpha-ketoacid decarboxylase	CP000029.1:2269462-2271111	1.177103	0.010688	0.024686	UP
SE1061	SERP1061	transcriptional regulator, Fur family	CP000029.1:1106174-1106623	1.259764	0.010771	0.024822	UP
SE1453	SERP1453	-	CP000029.1:1518624-1519721	1.20577	0.01076	0.024822	UP
SE2320	SERP2320	Glycine betaine transporter OpuD	CP000029.1:2357317-2358909	1.163772	0.011035	0.025402	UP
SE0309	SERP0309	Arylesterase/monoxygenase	CP000029.1:312317-313366	1.247083	0.01118	0.02571	UP
SE1830	rplD	50S ribosomal protein L4	CP000029.1:1860770-1861393	1.157107	0.011221	0.025774	UP
SE1441	camS	lipoprotein, putative	CP000029.1:1504373-1505581	1.211592	0.011424	0.026212	UP
SE0213	SERP0213	conserved hypothetical protein	CP000029.1:224430-225095	0.024585	0.011649	0.0267	-
SE2280	SERP2280	preprotein translocase, SecY subunit	CP000029.1:2314963-2316162	1.264755	0.011682	0.026748	UP
SE2159	SERP2159	Uncharacterized protein	CP000029.1:2188159-2189496	-0.01423	0.012285	0.028096	-
SE1477	SERP1477	ABC transporter, ATP-binding protein	CP000029.1:1540794-1541708	0.115796	0.012358	0.028233	-
SE1149	grpE	GrpE protein	CP000029.1:1186361-1186993	0.099484	0.012379	0.028252	-
SE0078	pfoR	membrane protein PfoR	CP000029.1:65272-66300	0.07828	0.012479	0.028449	-
SE1202	yajC	preprotein translocase, YajC subunit	CP000029.1:1241744-1242004	1.234707	0.012553	0.028587	UP
SE1249	SERP1249	transcriptional regulator, NrdR family	CP000029.1:1286173-1286643	-0.76848	0.012664	0.028808	-
SE1997	SERP1997	Formate/nitrite transporter, putative	CP000029.1:2016065-2016886	1.312871	0.012849	0.029197	UP
SE0662	SERP0662	-	CP000029.1:657785-658360	-0.15037	0.012891	0.029261	-
SE2071	SERP2071	glycine oxidase	CP000029.1:2097006-2098124	-0.44548	0.013018	0.029518	-
SE2412	mqo-4	malate:quinone-oxidoreductase	CP000029.1:2473294-2474778	0.124862	0.013082	0.02963	-
SE1303	SERP1303	conserved hypothetical protein	CP000029.1:1353554-1354408	1.304003	0.013325	0.030148	UP
SE0434	SERP0434	conserved hypothetical protein	CP000029.1:438804-439790	1.167437	0.013834	0.031267	UP
SE0303	abcA	Uncharacterized protein	CP000029.1:304158-305885	0.1253	0.01393	0.031391	-
SE1317	SERP1317	rhodanese-like domain protein	CP000029.1:1377104-1377415	1.97251	0.013932	0.031391	UP
SE1420	SERP1420	-	CP000029.1:1482813-1483097	0.098866	0.013933	0.031391	-
SE0080	SERP0080	cobalamin synthesis protein, putative	CP000029.1:67369-68571	1.816444	0.014	0.031507	UP
SE0405	murB	UDP-N-acetylenolpyruvoylglucosamine reductase	CP000029.1:406238-407158	0.084388	0.014031	0.031545	-
SE2222	cadC	transcriptional regulator, ArsR family	CP000029.1:2258429-2258776	2.594091	0.014306	0.032128	UP
SE2405	SERP2405	sensor histidine kinase	CP000029.1:2465380-2466918	1.297928	0.014469	0.03246	UP
SE0143	SERP0143	Uncharacterized protein	CP000029.1:135912-137102	1.16757	0.014559	0.032625	UP
SE0089	SERP0089	PAP2 domain protein	CP000029.1:75248-75919	1.366645	0.014666	0.032832	UP
SE0442	gapA-1	Glyceraldehyde-3-phosphate dehydrogenase 1	CP000029.1:446473-447483	0.134148	0.014775	0.03304	-
SE1926	galM	Aldose 1-epimerase	CP000029.1:1944600-1945610	-0.00828	0.014806	0.033075	-
SE0574	oppA	Oligopeptide ABC transporter, substrate-binding protei	CP000029.1:567431-569074	0.09272	0.014922	0.033264	-
SE0767	pyrC	dihydroorotase	CP000029.1:765157-766434	0.134459	0.014921	0.033264	-
SE1901	SERP1901	Uncharacterized HTH-type transcriptional regulator Yb	CP000029.1:1922994-1923866	0.135932	0.01534	0.034159	-
SE0149	hpt	Hypoxanthine phosphoribosyltransferase	CP000029.1:139858-140397	1.574689	0.015487	0.034449	UP
SE0382	SERP0382	Putative zinc transporter	CP000029.1:380323-381138	1.214301	0.015511	0.034467	UP
SE1216	SERP1216	-	CP000029.1:1251437-1251616	-0.79831	0.015604	0.034638	-
SE2060	glpT	Glycerol-3-phosphate transporter	CP000029.1:2085955-2087313	0.111166	0.015734	0.034889	-
SE0318	SERP0318	cell wall hydrolase, putative	CP000029.1:322334-323134	0.133874	0.015895	0.03521	-
SE0768	carA	Carbamoyl-phosphate synthase small chain	CP000029.1:766435-767535	0.141407	0.01636	0.036202	-
SE2552	dnaN	DNA polymerase III, beta subunit	CP000029.1:2613533-2614666	0.139786	0.016429	0.036316	-
SE0586	relA-1	GTP pyrophosphokinase, putative	CP000029.1:579345-579980	0.076579	0.01653	0.036488	-
SE0953	SERP0953	Putative oligopeptide transport system permease protein	CP000029.1:969316-970317	1.227672	0.016557	0.036488	UP
SE2306	hisG	ATP phosphoribosyltransferase	CP000029.1:2345799-2346413	3.09668	0.016576	0.036488	UP
SE2307	SERP2307	-	CP000029.1:2346438-2347250	3.09668	0.016576	0.036488	UP
SE1290	SERP1290	Escherichia coli K-12	CP000029.1:1334668-1336140	1.137934	0.016653	0.03662	UP
SE0183	rpoB	DNA-directed RNA polymerase subunit beta	CP000029.1:185929-189480	1.124561	0.01676	0.036816	UP
SE0762	lspA	Lipoprotein signal peptidase	CP000029.1:760458-760943	1.672077	0.016843	0.036959	UP
SE0362	SERP0362	oxidoreductase, aldo/keto reductase family	CP000029.1:362236-363075	0.139607	0.017048	0.037361	-
SE1686	SERP1686	membrane protein, putative	CP000029.1:1728531-1730036	1.162941	0.017061	0.037361	UP
SE2343	SERP2343	Escherichia coli K-12	CP000029.1:2389604-2389978	0.140223	0.017118	0.037446	-
SE1970	SERP1970	-	CP000029.1:1989454-1989549	Inf (+)	0.017298	0.037801	UP
SE0050	SERP0050	-	CP000029.1:42835-42930	0.08653	0.017328	0.037827	-
SE2212	SERP2212	-	CP000029.1:2248885-2250222	0.041377	0.017493	0.038149	-
SE0577	SERP0577	ISChy2, transposase	CP000029.1:571097-572110	-0.50548	0.017533	0.038197	-
SE0729	uvrC	excinuclease ABC, C subunit	CP000029.1:727888-729672	1.175265	0.017983	0.039137	UP
SE0751	ftsZ	Cell division protein FtsZ	CP000029.1:749673-750857	1.119712	0.018122	0.039398	UP
SE0989	arlR	CovR	CP000029.1:1007068-1007727	-0.01259	0.018419	0.040003	-
SE0283	SERP0283	membrane protein, putative	CP000029.1:286754-287098	0.141816	0.018967	0.041133	-
SE1951	SERP1951	ABC transporter, ATP-binding protein	CP000029.1:1966538-1967206	-0.33882	0.018998	0.041133	-
SE2149	SERP2149	-	CP000029.1:2176013-2177023	1.13477	0.018997	0.041133	UP
SE1013	SERP1013	conserved hypothetical protein	CP000029.1:1055221-1058658	1.149823	0.019181	0.041488	UP
SE1051	fer	ferredoxin	CP000029.1:1098180-1098428	-0.02972	0.019317	0.041695	-
SE1263	accA	acetyl-CoA carboxylase, carboxyl transferase, alpha sub	CP000029.1:1304973-1305917	1.318435	0.019317	0.041695	UP
SE0466	SERP0466	cold shock protein CspC	CP000029.1:468971-469171	0.142252	0.019387	0.041761	-
SE1440	putP	Sodium/proline symporter	CP000029.1:1502510-1504045	1.118649	0.019373	0.041761	UP
SE1910	SERP1910	HTH-type transcriptional regulator GlvR	CP000029.1:1930183-1930944	0.049547	0.019453	0.04186	-
SE0235	SERP0235	conserved hypothetical protein	CP000029.1:241677-242426	0.107636	0.019661	0.042222	-
SE1111	SERP1111	conserved hypothetical protein TIGR00106	CP000029.1:1151836-1152162	1.163934	0.019661	0.042222	UP
SE0700	SERP0700	conserved hypothetical protein	CP000029.1:696235-696717	0.044207	0.019682	0.042224	-
SE1119	sodA	superoxide dismutase, Mn	CP000029.1:1158272-1158871	0.155323	0.019808	0.042451	-

SE0642	SERP0642	Escherichia coli K-12	CP000029.1:636822-637730	0.126112	0.020077	0.042984	-
SE0783	sun	sun protein	CP000029.1:782172-783479	1.151853	0.020203	0.04321	UP
SE2394	SERP2394	8-amino-7-oxononanoate synthase	CP000029.1:2450579-2451712	-0.0464	0.020353	0.043487	-
SE0866	glpF	Uncharacterized protein	CP000029.1:879638-880462	1.111023	0.020786	0.044367	UP
SE1094	SERP1094	proline dipeptidase	CP000029.1:1138550-1139611	1.130808	0.021056	0.044897	UP
SE0667	SERP0667	conserved hypothetical protein	CP000029.1:662954-664126	1.194792	0.021454	0.045655	UP
SE1889	SERP1889	Uncharacterized protein	CP000029.1:1911830-1912954	1.365236	0.021448	0.045655	UP
SE0859	SERP0859	conserved hypothetical protein	CP000029.1:870883-871173	-0.06567	0.021521	0.045751	-
SE0398	nrdF-1	Ribonucleotide-disphosphate reductase beta chain, puta	CP000029.1:400714-401682	0.13302	0.021582	0.045835	-
SE2245	SERP2245	S-formylglutathione hydrolase FrmB	CP000029.1:2272878-2273636	-0.15267	0.021804	0.046259	-
SE0937	trpE	anthranilate synthase component I	CP000029.1:954084-955490	-0.72789	0.021926	0.046472	-
SE0969	SERP0969	alanine racemase	CP000029.1:987489-988562	1.129835	0.022056	0.0467	UP
SE1056	rluB	Pseudouridine synthase	CP000029.1:1102573-1103310	1.464763	0.022218	0.046997	UP
SE1122	SERP1122	cation ABC transporter, ATP-binding protein, putative	CP000029.1:1160580-1161362	1.166637	0.022451	0.047393	UP
SE2278	SERP2278	Accessory Sec system protein Asp2	CP000029.1:2311840-2313408	1.13248	0.022446	0.047393	UP
SE0813	sucC	succinyl-CoA synthase, beta subunit	CP000029.1:815834-817000	1.103588	0.022488	0.047424	UP
SE1384	SERP1384	DNA-binding response regulator, LuxR family	CP000029.1:1441233-1441856	1.535153	0.022755	0.04794	UP
SE1258	gltA	citrate synthase CitZ	CP000029.1:1297506-1298627	0.167066	0.023113	0.048646	-
SE1781	SERP1781	Dictyostelium discoideum	CP000029.1:1826305-1828266	1.246596	0.023163	0.048703	UP
SE1386	SERP1386	Ribosomal large subunit pseudouridine synthase, RluD	CP000029.1:1443141-1443965	0.157922	0.023216	0.048765	-
SE0171	SERP0171	conserved hypothetical protein	CP000029.1:178675-179073	1.263419	0.023262	0.048812	UP
SE1271	pepQ	proline dipeptidase	CP000029.1:1315670-1316725	0.158764	0.023641	0.049558	-
SE0581	SERP0581	UPF0413 protein YjbH	CP000029.1:576839-577636	0.15459	0.024119	0.05051	-
SE1232	hemB	Delta-aminolevulinic acid dehydratase	CP000029.1:1267603-1268577	1.110137	0.024161	0.050548	-
SE0013	SERP0013	-	CP000029.1:8913-9542	0.050438	0.024269	0.05068	-
SE0678	def	Peptide deformylase	CP000029.1:673977-674528	0.170635	0.024272	0.05068	-
SE1868	SERP1868	Urea transporter, putative	CP000029.1:1893690-1894592	0.019747	0.024489	0.051083	-
SE0026	SERP0026	-	CP000029.1:23557-23814	-1.99057	0.024635	0.051209	-
SE1748	SERP1748	general stress protein	CP000029.1:1786326-1786772	0.169045	0.024647	0.051209	-
SE2050	SERP2050	-	CP000029.1:2072959-2073105	-1.99057	0.024635	0.051209	-
SE2385	SERP2385	ferrous iron transport protein B, putative	CP000029.1:2434880-2436262	-1.99057	0.024635	0.051209	-
SE0555	rexA	ATP-dependent helicase/nuclease subunit A	CP000029.1:542830-546486	0.169611	0.024869	0.05162	-
SE1656	SERP1656	-	CP000029.1:1694543-1694824	2.593898	0.025043	0.05193	-
SE0552	spsA	Signal peptidase I	CP000029.1:538128-538649	1.896595	0.02513	0.05206	-
SE1026	birA	birA bifunctional protein	CP000029.1:1072181-1073152	1.194656	0.025188	0.05213	-
SE2021	SERP2021	Membrane protein insertase YidC 2	CP000029.1:2040235-2041071	1.179901	0.025257	0.052221	-
SE1276	SERP1276	conserved hypothetical protein	CP000029.1:1320528-1321475	0.16167	0.025531	0.052736	-
SE1685	SERP1685	conserved hypothetical protein	CP000029.1:1728035-1728544	1.410191	0.026408	0.054494	-
SE0855	SERP0855	Uncharacterized protein ymdB	CP000029.1:866089-866883	1.215549	0.026579	0.054793	-
SE2145	SERP2145	-	CP000029.1:2172847-2173116	3.709012	0.02678	0.055155	-
SE1185	trmU	tRNA-specific 2-thiouridylase MnmA	CP000029.1:1220766-1221884	1.135939	0.027139	0.055839	-
SE0507	SERP0507	CBS/transporter domain protein	CP000029.1:493871-494896	0.173968	0.027482	0.056489	-
SE0227	SERP0227	choloylglycine hydrolase family protein	CP000029.1:235619-236599	-0.90834	0.027679	0.056839	-
SE1254	polA	DNA polymerase	CP000029.1:1290547-1293177	1.09996	0.02856	0.058591	-
SE1696	SERP1696	HD domain protein	CP000029.1:1739357-1740010	1.502144	0.029157	0.059758	-
SE2038	SERP2038	putative iron ABC exporter ATP-binding subunit FetA	CP000029.1:2059580-2060242	0.066651	0.029452	0.060303	-
SE0255	SERP0255	conserved hypothetical protein TIGR00268	CP000029.1:262763-263590	1.097625	0.029695	0.060624	-
SE1391	SERP1391	Putative tRNA (cytidine(34)-2'-O)-methyltransferase	CP000029.1:1447416-1447886	1.282862	0.029689	0.060624	-
SE1704	ywpF	-	CP000029.1:1745923-1746363	-0.02444	0.029684	0.060624	-
SE0336	SERP0336	yaiI/yqxD family protein	CP000029.1:338170-338619	0.157597	0.030567	0.062346	-
SE0510	SERP0510	5'-nucleotidase family protein	CP000029.1:497037-498356	0.160039	0.030887	0.062937	-
SE0922	SERP0922	4-hydroxybenzoyl-CoA thioesterase, putative	CP000029.1:935179-935646	0.1466	0.031322	0.063762	-
SE1357	SERP1357	o-succinylbenzoate synthase	CP000029.1:1413267-1414268	1.132654	0.031564	0.06413	-
SE1778	SERP1778	alanine racemase	CP000029.1:1822209-1823270	0.118222	0.03156	0.06413	-
SE0818	xerC	tyrosine recombinase XerC	CP000029.1:823416-824306	1.112789	0.031616	0.064175	-
SE0429	lgt	prolipoprotein diacylglyceryl transferase	CP000029.1:433936-434775	1.131373	0.03202	0.064933	-
SE0809	SERP0809	-	CP000029.1:811159-813759	-0.09941	0.032319	0.065475	-
SE0723	rnhC	Ribonuclease HIII	CP000029.1:721102-722028	0.110185	0.032372	0.06552	-
SE1448	SERP1448	UPF0316 protein LMOf2365_1801	CP000029.1:1513101-1513727	1.12577	0.032778	0.066279	-
SE1726	tdk	Thymidine kinase	CP000029.1:1763162-1763761	1.494529	0.033115	0.066896	-
SE0882	SERP0882	-	CP000029.1:895252-895935	0.165382	0.033154	0.066909	-
SE1360	SERP1360	-	CP000029.1:1416933-1417598	Inf (+)	0.033617	0.067714	-
SE2256	SERP2256	-	CP000029.1:2285300-2285470	Inf (+)	0.033617	0.067714	-
SE1011	ebh	Surface antigen SasC	CP000029.1:1023531-1054142	0.186309	0.033758	0.067935	-
SE1151	SERP1151	Oxygen-independent coproporphyrinogen-III oxidase-lil	CP000029.1:1188099-1189223	1.27406	0.034116	0.068589	-
SE0045	ssb	single-stranded DNA-binding protein	CP000029.1:40716-41225	1.068838	0.034754	0.069807	-
SE2030	SERP2030	Amino acid ABC transporter, permease protein, putativ	CP000029.1:2050025-2050660	1.070214	0.03485	0.069931	-
SE2116	SERP2116	Holin-like protein CidB	CP000029.1:2141687-2142376	0.201922	0.035173	0.070513	-
SE1884	SERP1884	Putative secreted antigen GbpB/SagA putative peptidog	CP000029.1:1909383-1909856	1.403371	0.035284	0.070669	-
SE1312	SERP1312	Pseudouridine synthase	CP000029.1:1361364-1362056	1.137792	0.035569	0.071172	-
SE1899	SERP1899	glucokinase regulator-related protein	CP000029.1:1920656-1921543	0.20233	0.035728	0.071423	-
SE2115	SERP2115	Pyruvate oxidase	CP000029.1:2139898-2141637	0.203778	0.035771	0.07144	-
SE2202	SERP2202	-	CP000029.1:2236877-2237467	0.192758	0.035977	0.071783	-
SE1900	SERP1900	PTS system EIIBC component SAOUHSC_00158	CP000029.1:1921556-1922980	0.20468	0.036394	0.072547	-
SE1879	SERP1879	Amino-terminal domain similar to transcription regulato	CP000029.1:1901886-1903859	1.278028	0.036475	0.072641	-
SE2109	SERP2109	Aspergillus niger contig An02c0010, genomic contig	CP000029.1:2132809-2133789	1.071174	0.036888	0.073394	-

SE0021	SERP0021	ABC transporter, ATP-binding protein	CP000029.1:17991-18749	-0.61239	0.037098	0.073624	-
SE0046	rpsR	30S ribosomal protein S18	CP000029.1:41270-41512	1.284731	0.03704	0.073624	-
SE0755	ylmG	ylmG protein	CP000029.1:753377-753667	0.195927	0.037108	0.073624	-
SE0496	sufC	FeS assembly ATPase SufC	CP000029.1:485551-486312	1.124102	0.037254	0.073704	-
SE0764	pyrR	Bifunctional protein PyrR	CP000029.1:762165-762692	0.103616	0.037222	0.073704	-
SE0766	pyrB	Aspartate carbamoyltransferase	CP000029.1:764258-765139	1.070032	0.037236	0.073704	-
SE1449	nadE	NH(3)-dependent NAD(+) synthetase	CP000029.1:1513801-1514628	1.07545	0.038249	0.075602	-
SE1060	xerD	integrase/recombinase XerD	CP000029.1:1105244-1106131	1.092664	0.038398	0.075755	-
SE2210	SERP2210	CDP-glycerol:poly(glycerophosphate) glycerophosphotr	CP000029.1:2245847-2248180	1.175107	0.038366	0.075755	-
SE0040	SERP0040	conserved hypothetical protein	CP000029.1:37393-37596	-0.99097	0.038474	0.075835	-
SE0358	fruK	1-phosphofructokinase	CP000029.1:356011-356931	1.246798	0.038698	0.076204	-
SE2084	aldA-2	Putative aldehyde dehydrogenase AldA	CP000029.1:2111487-2112980	0.208985	0.038852	0.076437	-
SE2029	SERP2029	Amino acid transporter, putative	CP000029.1:2049064-2050008	1.077513	0.039132	0.076915	-
SE0129	SERP0129	Uncharacterized protein	CP000029.1:120524-121294	1.073229	0.039859	0.078199	-
SE0205	SERP0205	Uncharacterized protein	CP000029.1:214448-215326	0.112932	0.039837	0.078199	-
SE2411	SERP2411	D-arabinitol 2-dehydrogenase	CP000029.1:2471753-2472520	1.08659	0.041278	0.080909	-
SE0443	pgk	phosphoglycerate kinase	CP000029.1:447677-448867	0.21561	0.041326	0.080926	-
SE1321	SERP1321	Uncharacterized protein	CP000029.1:1382418-1382981	1.14745	0.041803	0.081785	-
SE2360	SERP2360	L-carnitine dehydrogenase	CP000029.1:2405308-2406273	-0.5759	0.042539	0.083148	-
SE0692	SERP0692	NRAMP metal ion transporter 6	CP000029.1:687766-689127	1.050205	0.042751	0.083346	-
SE0837	rbfA	ribosome-binding factor A	CP000029.1:845291-845641	0.050515	0.042759	0.083346	-
SE1196	relA-2	GTP pyrophosphokinase	CP000029.1:1232851-1235040	1.06859	0.042738	0.083346	-
SE1488	SERP1488	hydrolase, carbon-nitrogen family	CP000029.1:1556812-1557606	0.057998	0.043687	0.085076	-
SE0198	SERP0198	Putative CDP-glycerol:glycerophosphate glycerophosph	CP000029.1:207126-208820	1.52008	0.043748	0.085116	-
SE1906	SERP1906	-	CP000029.1:1926619-1926807	1.916079	0.043957	0.085446	-
SE0560	SERP0560	pyridine nucleotide-disulfide oxidoreductase, class I	CP000029.1:550615-551931	0.203707	0.044247	0.085929	-
SE0154	folB	dihydroneopterin aldolase	CP000029.1:146271-146636	3.593512	0.044306	0.085965	-
SE1971	SERP1971	-	CP000029.1:1989550-1989660	1.774583	0.044449	0.086164	-
SE0295	tagA	glycosyl transferase, WecB/TagA/CpsF family	CP000029.1:297389-298147	1.101022	0.044789	0.086685	-
SE1265	SERP1265	malate dehydrogenase, putative	CP000029.1:1306873-1308102	1.139502	0.0448	0.086685	-
SE0792	SERP0792	phosphatase, putative	CP000029.1:790811-792469	1.071835	0.045092	0.087114	-
SE1150	hrcA	Heat-inducible transcription repressor HrcA	CP000029.1:1187022-1187999	0.221073	0.045146	0.087114	-
SE2098	SERP2098	-	CP000029.1:2122124-2122909	1.516031	0.045119	0.087114	-
SE0942	trpB	Tryptophan synthase beta chain	CP000029.1:958460-959668	-0.79823	0.045281	0.087135	-
SE1080	recN	DNA repair protein RecN	CP000029.1:1125888-1127564	1.054287	0.045275	0.087135	-
SE1337	SERP1337	-	CP000029.1:1396194-1396502	-0.79823	0.045281	0.087135	-
SE1368	hemE	Uroporphyrinogen decarboxylase	CP000029.1:1425049-1426083	0.009106	0.04552	0.087516	-
SE0256	SERP0256	conserved hypothetical protein	CP000029.1:263610-264884	1.058216	0.046285	0.088905	-
SE0131	ksgA	Ribosomal RNA small subunit methyltransferase A	CP000029.1:122047-122937	1.210726	0.046598	0.089426	-
SE1874	ureG	Urease accessory protein UreG	CP000029.1:1898456-1899070	0.199939	0.046859	0.089844	-
SE0371	SERP0371	7-carboxy-7-deazaguanine synthase	CP000029.1:368937-369575	1.173954	0.047091	0.090207	-
SE0379	SERP0379	sulfatase	CP000029.1:375930-377870	1.042423	0.047569	0.09104	-
SE2205	SERP2205	sensor histidine kinase	CP000029.1:2240445-2241350	0.180286	0.047716	0.09124	-
SE1812	rplO	50S ribosomal protein L15	CP000029.1:1853215-1853655	0.227325	0.04797	0.091643	-
SE1204	queA	S-adenosylmethionine:tRNA ribosyltransferase-isomera	CP000029.1:1243180-1244205	1.055615	0.048028	0.09167	-
SE0287	SERP0287	Na+/H+ antiporter component	CP000029.1:289343-289807	1.084462	0.048581	0.092474	-
SE0770	pyrF	Orotidine 5'-phosphate decarboxylase	CP000029.1:770736-771428	1.043197	0.048497	0.092474	-
SE2175	SERP2175	N-acyl-L-amino acid amidohydrolase	CP000029.1:2205477-2206652	1.081649	0.048551	0.092474	-
SE0014	SERP0014	efflux transporter, RND family, MFP subunit	CP000029.1:9780-10898	2.009129	0.048953	0.092918	-
SE0986	sucA	2-oxoglutarate dehydrogenase E1 component	CP000029.1:1002485-1005289	1.036684	0.049034	0.092918	-
SE1021	SERP1021	-	CP000029.1:1066270-1066602	2.009129	0.048953	0.092918	-
SE1969	SERP1969	-	CP000029.1:1988714-1989091	-0.75632	0.048953	0.092918	-
SE1974	SERP1974	-	CP000029.1:1993044-1993394	1.103988	0.049011	0.092918	-
SE1354	SERP1354	-	CP000029.1:1411712-1412497	1.190704	0.049392	0.093512	-
SE0928	SERP0928	transcription antiterminator GlcT	CP000029.1:943312-944160	0.085332	0.049477	0.09359	-
SE2305	hisD	Histidinol dehydrogenase	CP000029.1:2344562-2345806	1.709495	0.050103	0.094689	-
SE1126	SERP1126	tRNA (adenine(22)-N(1))-methyltransferase	CP000029.1:1164951-1165640	1.346977	0.05024	0.094863	-
SE1451	SERP1451	Nitric oxide synthase oxygenase	CP000029.1:1516282-1517349	-0.10638	0.050395	0.09507	-
SE0319	SERP0319	-	CP000029.1:323545-324204	0.116009	0.050692	0.095546	-
SE0218	SERP0218	HAD hydrolase, family IA, variant 1	CP000029.1:227914-228561	-0.40596	0.050868	0.095792	-
SE0189	tuf	Elongation factor Tu	CP000029.1:197288-198472	0.234639	0.051292	0.096504	-
SE0286	SERP0286	monovalent cation/proton antiporter subunit, putative	CP000029.1:289066-289368	1.111242	0.051441	0.096699	-
SE1843	SERP1843	transporter, AcrB/AcrD/AcrF family	CP000029.1:1870861-1874016	1.033105	0.051716	0.097129	-
SE1278	SERP1278	membrane protein, putative	CP000029.1:1322341-1323105	0.213273	0.051822	0.097241	-
SE0234	SERP0234	conserved hypothetical protein	CP000029.1:240410-241174	0.082469	0.052243	0.097944	-
SE0735	SERP0735	phosphoesterase, putative	CP000029.1:734975-735484	1.133222	0.052393	0.098137	-
SE0104	SERP0104	-	CP000029.1:89012-89131	-0.23602	0.052712	0.098647	-
SE0186	rpsL	30S ribosomal protein S12	CP000029.1:193914-194327	1.049328	0.052784	0.098696	-
SE0709	SERP0709	ComK regulator	CP000029.1:706878-707312	1.168758	0.05347	0.09989	-
SE0207	SERP0207	Cna B-type domain-containing protein	CP000029.1:216458-219136	1.339761	0.054399	0.101333	-
SE0628	SERP0628	-	CP000029.1:619362-619457	-0.01137	0.054361	0.101333	-
SE0632	menB	1,4-dihydroxy-2-naphthoyl-CoA synthase	CP000029.1:623346-624164	1.031309	0.05434	0.101333	-
SE2087	SERP2087	D-lactate dehydrogenase, putative	CP000029.1:2114267-2115259	1.561686	0.054434	0.101333	-
SE1257	icd	Isocitrate dehydrogenase [NADP]	CP000029.1:1296195-1297463	0.24133	0.055471	0.103172	-
SE0341	SERP0341	ABC transporter, CydDC cysteine exporter (CydDC-E)	CP000029.1:342960-344654	1.062342	0.05576	0.103618	-
SE1379	SERP1379	conserved hypothetical protein	CP000029.1:1434373-1437312	1.028446	0.05585	0.103693	-

SE1735	rpoE	DNA-directed RNA polymerase, delta subunit	CP000029.1:1774849-1775385	1.056503	0.056115	0.104094	-
SE0895	SERP0895	-	CP000029.1:905539-906138	0.224352	0.056299	0.104344	-
SE0605	SERP0605	-	CP000029.1:597258-598451	1.03207	0.056489	0.104604	-
SE0330	SERP0330	membrane protein, putative	CP000029.1:334098-334586	0.230472	0.056807	0.1051	-
SE1687	SERP1687	conserved hypothetical protein	CP000029.1:1730029-1730508	-0.12842	0.057001	0.105367	-
SE0435	SERP0435	Putative sporulation transcription regulator WhiA	CP000029.1:439919-440863	1.058442	0.057888	0.106913	-
SE1184	SERP1184	TPR domain protein	CP000029.1:1219935-1220603	0.104952	0.057981	0.106991	-
SE0001	rpmH	ribosomal protein L34	CP000029.1:540-677	-0.01398	0.058476	0.107716	-
SE0322	SERP0322	Probable transcriptional regulatory protein SAOUHSC_	CP000029.1:327368-328084	1.293543	0.058476	0.107716	-
SE0468	SERP0468	-	CP000029.1:470040-470324	1.845559	0.058666	0.107973	-
SE1498	SERP1498	Ammonium transporter	CP000029.1:1564992-1566242	1.269618	0.05941	0.109245	-
SE2255	SERP2255	-	CP000029.1:2284405-2285319	0.056931	0.059512	0.109339	-
SE0216	SERP0216	3-hexulose-6-phosphate synthase	CP000029.1:226611-227243	0.246104	0.059607	0.109418	-
SE0426	uvrB	excinuclease ABC, B subunit	CP000029.1:427816-429801	1.070066	0.061229	0.112297	-
SE0105	SERP0105	-	CP000029.1:89245-90030	1.090023	0.061643	0.112926	-
SE0394	SERP0394	conserved hypothetical protein	CP000029.1:395984-396484	1.141293	0.061679	0.112926	-
SE1330	SERP1330	Gp58	CP000029.1:1392054-1392905	1.035835	0.061757	0.112972	-
SE0422	SERP0422	Putative secreted antigen GbpB/SagA putative peptidog	CP000029.1:425270-426070	1.594051	0.062208	0.113699	-
SE1267	SERP1267	DHH subfamily 1 protein	CP000029.1:1311627-1312565	1.055146	0.062476	0.11409	-
SE2262	SERP2262	Phage infection protein, putative	CP000029.1:2292637-2295501	1.027285	0.062729	0.114452	-
SE0100	SERP0100	Putative uncharacterized protein	CP000029.1:84899-85873	1.096562	0.063727	0.116173	-
SE1114	SERP1114	Rhomboid family protein	CP000029.1:1153332-1154792	1.029586	0.063796	0.116197	-
SE0320	SERP0320	Uncharacterized protein	CP000029.1:324467-326641	1.284748	0.063988	0.116446	-
SE2551	SERP2551	conserved hypothetical protein	CP000029.1:2612866-2613102	-0.299	0.064366	0.117034	-
SE0742	SERP0742	conserved hypothetical protein	CP000029.1:738368-739981	1.05092	0.064754	0.117537	-
SE2056	galU	UTP-glucose-1-phosphate uridylyltransferase	CP000029.1:2080818-2081684	1.025369	0.064717	0.117537	-
SE1806	rpsK	30S ribosomal protein S11	CP000029.1:1849907-1850296	0.254433	0.064821	0.117558	-
SE2397	SERP2397	-	CP000029.1:2454081-2454233	-0.15598	0.065102	0.117966	-
SE0449	est	Carboxylesterase, putative	CP000029.1:453949-454689	0.183673	0.065212	0.118021	-
SE1693	SERP1693	-	CP000029.1:1737008-1737217	0.172357	0.065244	0.118021	-
SE1814	rpsE	30S ribosomal protein S5	CP000029.1:1853871-1854371	0.25184	0.065512	0.118403	-
SE0056	SERP0056	-	CP000029.1:45300-45425	Inf (-)	0.065632	0.11846	-
SE1841	SERP1841	-	CP000029.1:1870031-1870201	1.532644	0.065655	0.11846	-
SE2020	SERP2020	-	CP000029.1:2040104-2040196	2.709591	0.066001	0.118982	-
SE2383	SERP2383	Uncharacterized protein	CP000029.1:2432528-2433523	-0.0189	0.066197	0.119234	-
SE1091	argJ	Arginine biosynthesis bifunctional protein ArgJ	CP000029.1:1135580-1136773	-0.01558	0.066878	0.120358	-
SE0797	fabG	3-oxoacyl-[acyl-carrier-protein] reductase FabG	CP000029.1:797345-798079	1.026835	0.067229	0.120886	-
SE2369	SERP2369	oligopeptide ABC transporter, ATP-binding protein	CP000029.1:2417086-2417901	-0.3832	0.067893	0.121873	-
SE2410	SERP2410	-	CP000029.1:2471339-2471446	1.74609	0.067893	0.121873	-
SE2274	SERP2274	Glycosyltransferase-stabilizing protein Gtf2	CP000029.1:2306039-2307379	0.221035	0.068277	0.122458	-
SE0627	menA	1,4-dihydroxy-2-naphthoate octaprenyltransferase, puta	CP000029.1:618425-619363	1.019469	0.068424	0.122617	-
SE0716	SERP0716	UPF0348 protein LMOf2365_2080	CP000029.1:711124-712257	1.139686	0.068529	0.1227	-
SE1469	SERP1469	-	CP000029.1:1534718-1535758	1.037735	0.069863	0.124983	-
SE2362	SERP2362	Putative transcriptional regulator	CP000029.1:2407305-2407844	-0.15136	0.07001	0.12514	-
SE0451	smpB	SsrA-binding protein	CP000029.1:457127-457597	0.240053	0.070294	0.125543	-
SE1135	cdd	Cytidine deaminase	CP000029.1:1174138-1174542	1.099617	0.071202	0.127056	-
SE1283	SERP1283	-	CP000029.1:1328209-1328325	-0.23187	0.071376	0.127258	-
SE1110	SERP1110	metallo-beta-lactamase family protein	CP000029.1:1151213-1151836	1.02194	0.071478	0.127334	-
SE1392	SERP1392	iron-sulfur cluster-binding protein, putative	CP000029.1:1447883-1449013	1.158335	0.071612	0.127465	-
SE0413	SERP0413	GGDEF domain protein	CP000029.1:413507-414583	1.034335	0.071765	0.127629	-
SE0017	SERP0017	-	CP000029.1:12806-12898	3.467954	0.072712	0.129204	-
SE0546	gluD	NAD-specific glutamate dehydrogenase	CP000029.1:530332-531576	0.267697	0.074461	0.132202	-
SE2089	srtA	LPXTG-site transpeptidase family protein	CP000029.1:2116600-2117211	0.12458	0.074573	0.132289	-
SE0455	SERP0455	-	CP000029.1:459310-459852	-2.57553	0.075793	0.134227	-
SE0943	trpA	Tryptophan synthase alpha chain	CP000029.1:959661-960392	-1.57553	0.075793	0.134227	-
SE0048	SERP0048	-	CP000029.1:41982-42242	-0.76853	0.076083	0.134516	-
SE0265	SERP0265	-	CP000029.1:272481-272732	-0.76853	0.076083	0.134516	-
SE0781	def-2	peptide deformylase	CP000029.1:780762-781250	0.081256	0.077467	0.136847	-
SE2422	SERP2422	-	CP000029.1:2481410-2482195	1.523649	0.078518	0.138588	-
SE0684	SERP0684	conserved hypothetical protein	CP000029.1:680746-681024	2.079445	0.079834	0.140676	-
SE2108	SERP2108	-	CP000029.1:2132420-2132737	2.009043	0.079834	0.140676	-
SE1425	SERP1425	-	CP000029.1:1487117-1487512	-0.05961	0.080694	0.142073	-
SE0436	clpP	ATP-dependent Clp protease proteolytic subunit	CP000029.1:441191-441775	0.261685	0.081001	0.142493	-
SE1950	SERP1950	Uncharacterized protein	CP000029.1:1965301-1966257	-0.02064	0.081098	0.142546	-
SE0114	dnaX	DNA polymerase III, gamma and tau subunits, putative	CP000029.1:102244-103950	1.033686	0.082019	0.143992	-
SE0516	SERP0516	D-isomer specific 2-hydroxyacid dehydrogenase, NAD	CP000029.1:501635-502606	0.275178	0.082079	0.143992	-
SE1701	SERP1701	transcriptional activator TenA	CP000029.1:1743316-1744005	0.093492	0.082125	0.143992	-
SE1727	rpmE	ribosomal protein L31	CP000029.1:1764048-1764305	1.000537	0.082539	0.144598	-
SE2317	pcp	Pyrrolidone-carboxylate peptidase	CP000029.1:2355905-2356543	0.079488	0.082894	0.145099	-
SE0306	SERP0306	iron compound ABC transporter, ATP-binding protein	CP000029.1:309129-309926	0.193222	0.083713	0.146048	-
SE0538	mnhA	Na(+)/H(+) antiporter subunit A	CP000029.1:520519-522924	0.991229	0.083552	0.146048	-
SE1047	SERP1047	Uncharacterized protein	CP000029.1:1093085-1094071	1.000799	0.083597	0.146048	-
SE2279	SERP2279	Gap1	CP000029.1:2313389-2314945	1.045779	0.083694	0.146048	-
SE0851	SERP0851	competence/damage-inducible protein CinA	CP000029.1:861468-862616	0.998697	0.085411	0.148829	-
SE0980	SERP0980	-	CP000029.1:997992-998195	1.015985	0.085448	0.148829	-
SE1992	nirR	cbiX domain protein	CP000029.1:2011906-2012625	-0.21335	0.085767	0.149261	-

SE0788	rpe	Ribulose-phosphate 3-epimerase	CP000029.1:788508-789152	0.099863	0.086536	0.150476	-
SE0058	SERP0058	-	CP000029.1:46212-46340	-0.13773	0.088013	0.152792	-
SE0975	SERP0975	-	CP000029.1:991736-992362	0.281513	0.087967	0.152792	-
SE0209	arsC-1	arsenate reductase	CP000029.1:221374-221772	2.231371	0.088489	0.153493	-
SE1268	SERP1268	thioesterase family protein	CP000029.1:1312599-1313897	0.262365	0.089846	0.155719	-
SE0817	SERP0817	gid protein	CP000029.1:821884-823191	1.004978	0.089976	0.155723	-
SE1490	agrB	Putative AgrB-like protein	CP000029.1:1558381-1558965	0.284045	0.089996	0.155723	-
SE0488	SERP0488	thioredoxin, putative	CP000029.1:480414-480710	-0.06888	0.090081	0.155741	-
SE0583	SERP0583	conserved hypothetical protein	CP000029.1:578106-578696	1.35489	0.090346	0.155945	-
SE1886	sugE-1	sugE protein	CP000029.1:1910009-1910335	-0.02407	0.090287	0.155945	-
SE2067	SERP2067	Uncharacterized membrane protein YvaC	CP000029.1:2091947-2093863	0.233491	0.09196	0.158601	-
SE2539	SERP2539	DHH subfamily 1 protein	CP000029.1:2596415-2598382	0.981146	0.092367	0.159173	-
SE0591	SERP0591	-	CP000029.1:585628-586251	1.652997	0.092535	0.159202	-
SE2041	SERP2041	Glutamate synthase alpha subunit, putative	CP000029.1:2061776-2063353	1.003462	0.092514	0.159202	-
SE2085	SERP2085	-	CP000029.1:2112980-2113111	-0.20504	0.093098	0.16004	-
SE0634	SERP0634	-	CP000029.1:625836-626843	1.275886	0.093809	0.161132	-
SE2368	SERP2368	peptide/opine/nickel uptake family ABC transporter, AT	CP000029.1:2416344-2417093	-0.57575	0.094398	0.162012	-
SE1769	SERP1769	hemolysin III	CP000029.1:1813801-1814484	0.224486	0.09554	0.163744	-
SE1865	SERP1865	Uncharacterized protein	CP000029.1:1890855-1891796	1.319438	0.095563	0.163744	-
SE2123	ogt	Methylated-DNA--protein-cysteine methyltransferase, p	CP000029.1:2148070-2148588	1.086338	0.095929	0.164238	-
SE1873	ureF	Urease accessory protein UreF	CP000029.1:1897754-1898443	0.285046	0.096171	0.16452	-
SE0102	SERP0102	-	CP000029.1:87880-88158	0.054175	0.098487	0.168346	-
SE0238	mvk	phosphomevalonate kinase, putative	CP000029.1:245118-246038	1.007249	0.098586	0.168378	-
SE2017	SERP2017	Amino acid ABC transporter-like protein, putative	CP000029.1:2034917-2036293	0.977964	0.099141	0.169189	-
SE1331	SERP1331	-	CP000029.1:1393283-1393498	-0.02264	0.100044	0.170591	-
SE0542	SERP0542	FMN oxidoreductase, putative	CP000029.1:525155-526282	0.981182	0.10046	0.171164	-
SE0857	SERP0857	pyruvate ferredoxin oxidoreductase, beta subunit, putati	CP000029.1:868757-869623	0.994391	0.101386	0.172602	-
SE0499	SERP0499	Uncharacterized protein	CP000029.1:489013-489477	0.990256	0.102557	0.174319	-
SE1073	SERP1073	-	CP000029.1:1119107-1119202	2.594091	0.102725	0.174319	-
SE1322	rot	-	CP000029.1:1383215-1383625	0.284791	0.102635	0.174319	-
SE1355	SERP1355	antimutator 8-oxo-(dGTP/GTP)ase	CP000029.1:1412484-1412960	2.594091	0.102725	0.174319	-
SE0122	SERP0122	DNA polymerase III, delta prime subunit, putative	CP000029.1:114318-115244	0.288521	0.10442	0.177053	-
SE0959	SERP0959	Phosphate transport system permease protein	CP000029.1:975510-976436	1.135642	0.105334	0.178458	-
SE0145	SERP0145	cell division protein DivIC	CP000029.1:137386-137787	1.276568	0.106102	0.179617	-
SE0372	SERP0372	6-pyruvoyl tetrahydrobiopterin synthase, putative	CP000029.1:369651-370070	1.413505	0.107922	0.182551	-
SE1700	thiD-2	phosphomethylpyrimidine kinase	CP000029.1:1742502-1743323	0.24714	0.109743	0.185482	-
SE1439	gatC	glutamyl-tRNA(Gln) amidotransferase, C subunit	CP000029.1:1501830-1502132	1.251954	0.109917	0.185629	-
SE1810	adk	Adenylate kinase	CP000029.1:1851258-1851905	0.3061	0.110046	0.185697	-
SE2419	SERP2419	-	CP000029.1:2479343-2479528	1.935028	0.111707	0.18835	-
SE2037	SERP2037	Membrane protein, putative	CP000029.1:2058811-2059587	0.966626	0.11296	0.190311	-
SE0865	glpP	glycerol uptake operon antiterminator regulatory proteir	CP000029.1:878730-879263	0.28511	0.113522	0.191104	-
SE0996	SERP0996	carboxyl-terminal protease	CP000029.1:1012526-1014001	0.982985	0.115239	0.193841	-
SE1053	SERP1053	conserved hypothetical protein	CP000029.1:1098833-1099372	0.286918	0.115338	0.193853	-
SE0044	rpsF	30S ribosomal protein S6	CP000029.1:40397-40693	0.983044	0.11578	0.193977	-
SE1194	SERP1194	Probable cell wall amidase LytH	CP000029.1:1231513-1232388	1.076208	0.1157	0.193977	-
SE1815	rplR	50S ribosomal protein L18	CP000029.1:1854391-1854753	0.308157	0.115594	0.193977	-
SE2122	SERP2122	Caenorhabditis elegans	CP000029.1:2146652-2147818	0.959959	0.115659	0.193977	-
SE0596	SERP0596	-	CP000029.1:590900-591028	1.267812	0.11674	0.19543	-
SE0173	SERP0173	conserved hypothetical protein	CP000029.1:179827-180354	0.987457	0.116897	0.195538	-
SE0276	SERP0276	Uncharacterized protein	CP000029.1:281490-282383	1.087104	0.117614	0.196431	-
SE1247	dnaI	Uncharacterized protein	CP000029.1:1283882-1284802	0.304585	0.117616	0.196431	-
SE2302	hisA	1-(5-phosphoribosyl)-5-[(5-phosphoribosylamino)methy	CP000029.1:2342596-2343300	3.330598	0.118117	0.197111	-
SE0961	SERP0961	conserved hypothetical protein	CP000029.1:978238-979134	1.141993	0.118237	0.197155	-
SE0481	aroD	Catabolic 3-dehydroquinate dehydratase	CP000029.1:476880-477596	0.192537	0.120308	0.200449	-
SE0589	mgtE	Magnesium transporter MgtE	CP000029.1:581679-583064	0.978337	0.121016	0.201471	-
SE0201	SERP0201	3-oxoacyl-[acyl-carrier-protein] reductase FabG	CP000029.1:211327-212079	0.209788	0.12123	0.201668	-
SE0005	gidB	glucose-inhibited division protein B	CP000029.1:4658-5377	1.000052	0.121462	0.201894	-
SE0153	folP	dihydropteroate synthase	CP000029.1:145475-146293	1.005723	0.121742	0.202041	-
SE0178	rplK	50S ribosomal protein L11	CP000029.1:182402-182824	0.952086	0.121681	0.202041	-
SE2347	bioB	biotin synthase	CP000029.1:2392998-2393963	-0.03674	0.122847	0.203716	-
SE1009	SERP1009	putative membrane protein	CP000029.1:1021935-1022639	1.091038	0.124279	0.205846	-
SE1137	SERP1137	conserved hypothetical protein TIGR00043	CP000029.1:1174900-1175367	0.310814	0.124327	0.205846	-
SE1144	SERP1144	Uncharacterized protein	CP000029.1:1180162-1181508	0.969204	0.125066	0.206907	-
SE1136	dgkA	diacylglycerol kinase	CP000029.1:1174542-1174898	0.969903	0.125691	0.207779	-
SE0477	SERP0477	Acetyltransferase	CP000029.1:474872-475372	0.958018	0.126169	0.208406	-
SE0775	fbe	fibronectin/fibrinogen-binding protein, putative	CP000029.1:773283-774980	1.084059	0.126598	0.208951	-
SE1842	SERP1842	-	CP000029.1:1870493-1870810	1.179002	0.12729	0.209928	-
SE2259	SERP2259	transcription antiterminator, BglG family	CP000029.1:2287464-2289335	0.228543	0.127458	0.210042	-
SE1706	murAA	UDP-N-acetylglucosamine 1-carboxyvinyltransferase	CP000029.1:1747152-1748417	0.959447	0.127659	0.21021	-
SE0491	SERP0491	ABC transporter, substrate-binding protein, putative	CP000029.1:482697-483509	0.303299	0.127947	0.21052	-
SE0816	topA	DNA topoisomerase I	CP000029.1:819790-821859	1.002182	0.128581	0.211398	-
SE2328	SERP2328	Sodium, sulfate symporter, putative	CP000029.1:2365343-2366761	0.950515	0.129016	0.211948	-
SE0660	SERP0660	Energy-coupling factor transporter transmembrane prote	CP000029.1:655569-656408	0.232869	0.12936	0.212192	-
SE0669	ptsH	Phosphocarrier protein HPr	CP000029.1:664881-665147	1.071642	0.129366	0.212192	-
SE2081	SERP2081	Uncharacterized protein	CP000029.1:2108088-2109035	0.151711	0.129868	0.212851	-
SE2026	lrgA	holin, putative	CP000029.1:2047024-2047482	0.943842	0.12998	0.212869	-

SE1454	SERP1454	isochorismatase family protein	CP000029.1:1519913-1520467	0.251299	0.130108	0.212913	-
SE1811	secY	preprotein translocase, SecY subunit	CP000029.1:1851923-1853215	0.323823	0.130231	0.212949	-
SE1495	cscA	Invertase	CP000029.1:1562172-1563644	0.971325	0.130602	0.213391	-
SE0874	SERP0874	aluminum resistance protein	CP000029.1:888383-889621	0.976491	0.130885	0.213688	-
SE0106	SERP0106	-	CP000029.1:90027-91139	1.039345	0.131047	0.213787	-
SE0279	SERP0279	-	CP000029.1:282773-282997	2.815947	0.131529	0.214242	-
SE0558	SERP0558	ISChy2, transposase	CP000029.1:548857-549870	-2.31262	0.131529	0.214242	-
SE2300	hisIE	Imidazole glycerol phosphate synthase subunit hisF, put	CP000029.1:2341209-2341844	1.381118	0.1317	0.214355	-
SE1090	argB	Acetylglutamate kinase	CP000029.1:1134833-1135570	-0.12215	0.13189	0.214498	-
SE1682	SERP1682	Antitoxin MazE	CP000029.1:1726162-1726332	-0.83899	0.13207	0.214627	-
SE0976	SERP0976	tellurite resistance protein	CP000029.1:992385-993515	0.944058	0.132775	0.215607	-
SE1417	SERP1417	aminopeptidase AmpS	CP000029.1:1480742-1481983	1.033151	0.132893	0.215631	-
SE0940	trpC	Indole-3-glycerol phosphate synthase	CP000029.1:957055-957843	-0.57586	0.133184	0.215937	-
SE1851	mobA	molybdopterin-guanine dinucleotide biosynthesis protei	CP000029.1:1880928-1881542	1.001195	0.13367	0.21656	-
SE2270	SERP2270	putative transport protein, major facilitator superfamily	CP000029.1:2302918-2304090	1.025282	0.133901	0.216768	-
SE0528	SERP0528	Probable cytosol aminopeptidase	CP000029.1:512262-513743	1.058018	0.134327	0.21716	-
SE0802	SERP0802	conserved hypothetical protein	CP000029.1:804248-804580	0.119283	0.134349	0.21716	-
SE0633	SERP0633	aminotransferase, classes I and II	CP000029.1:624509-625693	0.310952	0.136063	0.219762	-
SE1306	SERP1306	-	CP000029.1:1356033-1356347	0.241756	0.136589	0.220443	-
SE1946	SERP1946	transcriptional regulator, TetR family	CP000029.1:1961133-1961684	1.05092	0.136882	0.220747	-
SE0676	SERP0676	Ribonuclease J 1	CP000029.1:671644-673326	0.939825	0.137934	0.222274	-
SE0944	SERP0944	ThiJ/PfpI family protein	CP000029.1:960583-961242	0.327146	0.138866	0.223605	-
SE1327	ribE	riboflavin synthase, alpha subunit	CP000029.1:1388104-1388736	0.971794	0.139112	0.22383	-
SE0308	SERP0308	Putative membrane protein	CP000029.1:310971-312038	0.94033	0.13986	0.224863	-
SE0793	recG	ATP-dependent DNA helicase RecG	CP000029.1:792673-794721	0.946218	0.140309	0.225413	-
SE2100	rbsK	ribokinase	CP000029.1:2124965-2125888	0.967058	0.140648	0.225785	-
SE0745	ftsL	cell division protein FtsL	CP000029.1:741519-741920	1.170577	0.141135	0.226353	-
SE1820	rplX	50S ribosomal protein L24	CP000029.1:1856548-1856865	0.31348	0.141216	0.226353	-
SE2358	SERP2358	glycine betaine/L-proline ABC transporter, ATP-bindin	CP000029.1:2403501-2404688	1.456595	0.141576	0.226758	-
SE1353	pckA	Phosphoenolpyruvate carboxykinase [ATP]	CP000029.1:1409889-1411481	0.938666	0.142039	0.227327	-
SE0532	mnhG	monovalent cation/proton antiporter, MnhG/PhaG subu	CP000029.1:517160-517516	1.014724	0.14539	0.232515	-
SE1299	SERP1299	conserved hypothetical protein	CP000029.1:1347505-1347996	0.335686	0.145696	0.232827	-
SE2179	SERP2179	Choline transporter, putative	CP000029.1:2211424-2213028	0.954972	0.146164	0.233399	-
SE0824	tsf	Elongation factor Ts	CP000029.1:828421-829299	0.938709	0.147013	0.234576	-
SE1743	deoB	Phosphopentomutase	CP000029.1:1782086-1783276	0.333833	0.147389	0.234999	-
SE1442	ligA	DNA ligase	CP000029.1:1505601-1507598	0.332192	0.147583	0.235131	-
SE1655	SERP1655	inner membrane protein YeeE	CP000029.1:1694146-1694457	1.284698	0.14816	0.235872	-
SE1968	SERP1968	PTS system sucrose-specific IIBC component, putative	CP000029.1:1987115-1988560	0.302205	0.149288	0.237488	-
SE1074	SERP1074	conserved hypothetical protein	CP000029.1:1119254-1120231	0.953667	0.149926	0.238144	-
SE1156	SERP1156	ComE operon protein 2	CP000029.1:1195066-1195527	0.112179	0.149843	0.238144	-
SE1179	SERP1179	UPF0473 protein LMOf2365_1520	CP000029.1:1213465-1213773	0.300924	0.150525	0.238917	-
SE1443	pcrA	ATP-dependent DNA helicase PcrA	CP000029.1:1507602-1509791	0.336483	0.151381	0.240094	-
SE1300	murC	UDP-N-acetylmuramate--alanine ligase	CP000029.1:1348071-1349384	0.930749	0.151646	0.240334	-
SE1006	SERP1006	conserved hypothetical protein	CP000029.1:1020729-1021166	0.965157	0.151807	0.240408	-
SE1745	deoC	Deoxyribose-phosphate aldolase	CP000029.1:1784623-1785285	0.960813	0.152586	0.241281	-
SE2121	SERP2121	hydroxymethylglutaryl-CoA reductase, degradative	CP000029.1:2145130-2146407	0.285546	0.152504	0.241281	-
SE0091	SERP0091	-	CP000029.1:76769-77011	1.247871	0.155538	0.245765	-
SE1207	SERP1207	ACT domain protein pheB	CP000029.1:1245840-1246295	0.934884	0.155815	0.245901	-
SE1302	SERP1302	tRNA binding domain protein, putative	CP000029.1:1352935-1353531	0.980611	0.155858	0.245901	-
SE1494	SERP1494	AT3G59480	CP000029.1:1561206-1562165	0.945483	0.156145	0.24617	-
SE0671	SERP0671	glutaredoxin family protein	CP000029.1:667143-667379	1.057042	0.15783	0.24861	-
SE1333	SERP1333	-	CP000029.1:1393706-1394185	2.468533	0.157928	0.24861	-
SE0274	sarA	Transcriptional regulator SarA	CP000029.1:279424-279798	0.924086	0.158956	0.250042	-
SE1885	SERP1885	-	CP000029.1:1909922-1910014	0.285303	0.1592	0.250239	-
SE0029	SERP0029	DNA-binding protein	CP000029.1:24753-24956	1.816526	0.160156	0.250807	-
SE0233	SERP0233	putative membrane protein	CP000029.1:239945-240397	1.054616	0.159911	0.250807	-
SE1106	SERP1106	comG operon protein 4	CP000029.1:1148381-1148830	1.916079	0.160156	0.250807	-
SE1845	SERP1845	-	CP000029.1:1875743-1876504	-0.50552	0.160156	0.250807	-
SE1846	SERP1846	Uncharacterized protein	CP000029.1:1876667-1877107	-0.50552	0.160156	0.250807	-
SE1409	SERP1409	Teichoic acids export ATP-binding protein TagH	CP000029.1:1473664-1475181	1.002973	0.1621	0.253665	-
SE0197	SERP0197	sorbitol dehydrogenase	CP000029.1:206078-207109	1.350143	0.163084	0.255015	-
SE0871	SERP0871	host factor-I protein	CP000029.1:886079-886318	0.341881	0.165818	0.259098	-
SE0460	SERP0460	conserved hypothetical protein	CP000029.1:463394-463738	-0.15134	0.166093	0.259336	-
SE1032	SERP1032	-	CP000029.1:1077338-1077901	1.057675	0.167692	0.261639	-
SE0683	pdhD	pyruvate dehydrogenase complex E3 component, dihyd	CP000029.1:679038-680444	0.915302	0.168596	0.262855	-
SE2332	SERP2332	conserved hypothetical protein	CP000029.1:2369134-2369637	1.189659	0.169749	0.264458	-
SE1882	SERP1882	opine dehydrogenase, putative	CP000029.1:1907115-1908185	0.921428	0.170506	0.265441	-
SE1829	rplW	50S ribosomal protein L23	CP000029.1:1860495-1860770	0.928592	0.171554	0.266877	-
SE0785	SERP0785	protein phosphatase 2C, family protein	CP000029.1:784583-785326	0.965157	0.172886	0.26875	-
SE1018	SERP1018	-	CP000029.1:1062550-1062882	1.050911	0.173226	0.268908	-
SE2299	SERP2299	si:rp71-1f1.5	CP000029.1:2340315-2341100	1.294482	0.173243	0.268908	-
SE0386	SERP0386	Amino acid ABC transporter, permease protein, putative	CP000029.1:386108-387622	0.914301	0.173489	0.269092	-
SE1450	SERP1450	nicotinate phosphoribosyltransferase, putative	CP000029.1:1514621-1516063	0.338745	0.17555	0.27209	-
SE1939	SERP1939	Uncharacterized protein	CP000029.1:1955422-1955898	0.259796	0.17619	0.272881	-
SE0447	SERP0447	-	CP000029.1:452888-453346	0.927278	0.176765	0.273572	-
SE0540	SERP0540	Cyclophilin-type peptidyl-prolyl cis-trans isomerase	CP000029.1:523564-524157	0.94877	0.17711	0.273704	-

SE0838	truB	tRNA pseudouridine synthase B	CP000029.1:845779-846696	1.396143	0.177052	0.273704	-
SE1320	SERP1320	Uncharacterized protein	CP000029.1:1381468-1382421	0.16111	0.178377	0.275183	-
SE1817	rpsH	30S ribosomal protein S8	CP000029.1:1855346-1855744	0.355351	0.178458	0.275183	-
SE1990	nirB	nitrite reductase [NAD(P)H], large subunit	CP000029.1:2009063-2011468	0.350988	0.178456	0.275183	-
SE0085	SERP0085	conserved hypothetical protein	CP000029.1:71658-74225	0.959514	0.178816	0.275533	-
SE2356	SERP2356	Amino acid transporter, putative	CP000029.1:2401891-2402850	1.250107	0.180393	0.27776	-
SE0333	SERP0333	-	CP000029.1:336324-336620	0.275492	0.181654	0.279295	-
SE0756	ylmH	S4 domain protein	CP000029.1:753923-754585	0.297394	0.181649	0.279295	-
SE0059	ahpF	alkyl hydroperoxide reductase, F subunit	CP000029.1:46330-47853	0.360871	0.182494	0.280382	-
SE0590	SERP0590	Uncharacterized protein	CP000029.1:583074-584918	0.910873	0.184909	0.283885	-
SE0999	msrB	peptide methionine sulfoxide reductase	CP000029.1:1015083-1015511	0.360058	0.185154	0.284055	-
SE0070	guaA	GMP synthase [glutamine-hydrolyzing]	CP000029.1:58718-60259	0.908321	0.187222	0.287019	-
SE0687	potB	spermidine/putrescine ABC transporter, permease prote	CP000029.1:682801-683604	0.089273	0.188261	0.288232	-
SE1430	SERP1430	Mur ligase family protein	CP000029.1:1491431-1492744	0.211593	0.188286	0.288232	-
SE1183	SERP1183	helicase, putative	CP000029.1:1217501-1219933	0.929872	0.188772	0.288767	-
SE1699	thiM	hydroxyethylthiazole kinase	CP000029.1:1741721-1742509	0.312165	0.191448	0.292648	-
SE0547	SERP0547	glycerophosphoryl diester phosphodiesterase, putative	CP000029.1:531768-532817	1.313992	0.193122	0.294566	-
SE0686	potA	spermidine/putrescine ABC transporter, ATP-binding p	CP000029.1:681714-682808	1.378276	0.193029	0.294566	-
SE2544	hlb	sphingomyelinase C	CP000029.1:2602224-2603228	0.009089	0.193122	0.294566	-
SE0272	SERP0272	-	CP000029.1:277548-278267	0.914376	0.19357	0.295037	-
SE0023	SERP0023	-	CP000029.1:19519-19659	-0.669	0.194536	0.296082	-
SE0941	trpF	N-(5'-phosphoribosyl)anthranilate isomerase	CP000029.1:957833-958456	-0.669	0.194536	0.296082	-
SE1722	SERP1722	Threonylcarbamoyl-AMP synthase	CP000029.1:1760057-1761100	1.168281	0.195011	0.296591	-
SE1389	SERP1389	-	CP000029.1:1446456-1446635	1.594051	0.195373	0.296928	-
SE1029	SERP1029	conserved hypothetical protein	CP000029.1:1075495-1075812	1.539602	0.195974	0.297626	-
SE1405	SERP1405	conserved hypothetical protein	CP000029.1:1469792-1470334	1.047903	0.196403	0.298064	-
SE0556	SERP0556	fumarylacetoacetate hydrolase family protein	CP000029.1:546685-547590	0.368446	0.198727	0.301374	-
SE0936	tyrA	Prephenate dehydrogenase	CP000029.1:952337-953425	0.149281	0.199734	0.302684	-
SE0232	SERP0232	Uncharacterized protein	CP000029.1:238294-239784	0.365633	0.200305	0.303332	-
SE0242	SERP0242	putative oxidoreductase with FAD/NAD(P)-binding do	CP000029.1:249056-250387	0.963615	0.200959	0.304104	-
SE0789	SERP0789	thiamine pyrophosphokinase	CP000029.1:789157-789792	0.987727	0.202968	0.306924	-
SE2336	SERP2336	Lipase	CP000029.1:2373203-2375389	0.161097	0.203353	0.307286	-
SE1208	SERP1208	GTPase Obg	CP000029.1:1246307-1247599	0.904198	0.203948	0.307964	-
SE1954	SERP1954	sensor histidine kinase	CP000029.1:1969057-1970412	0.93671	0.204492	0.308565	-
SE1849	sarV	-	CP000029.1:1879141-1879491	1.070491	0.208332	0.314134	-
SE1030	SERP1030	neutral zinc metallopeptidase, putative	CP000029.1:1075993-1076688	0.376217	0.209152	0.314921	-
SE1740	SERP1740	amidohydrolase family protein	CP000029.1:1779706-1780890	0.941635	0.209102	0.314921	-
SE1979	sarZ	HTH-type transcriptional regulator SarZ	CP000029.1:1996404-1996850	0.321236	0.209853	0.315752	-
SE0550	pgi	phosphoglucose isomerase	CP000029.1:535900-537231	0.893666	0.210274	0.31616	-
SE1854	mobB	Molybdopterin-guanine dinucleotide biosynthesis protei	CP000029.1:1882222-1882698	1.257	0.210671	0.316532	-
SE0873	SERP0873	GTPase HflX	CP000029.1:887117-888367	0.333047	0.210867	0.316601	-
SE1663	SERP1663	Uncharacterized protein	CP000029.1:1701381-1702043	0.345789	0.211431	0.317222	-
SE0914	SERP0914	UPF0154 protein LMOf2365_1324	CP000029.1:923087-923314	1.774636	0.211789	0.317533	-
SE0794	SERP0794	Transcription factor FapR	CP000029.1:794867-795442	0.280766	0.212398	0.318078	-
SE0833	SERP0833	Transcription termination/antitermination protein NusA	CP000029.1:840979-842202	0.894968	0.212454	0.318078	-
SE0134	purR	pur operon repressor PurR	CP000029.1:124556-125380	0.374865	0.214804	0.321368	-
SE0548	argH	Argininosuccinate lyase	CP000029.1:532967-534358	0.307536	0.216873	0.324235	-
SE1698	thiE	Thiamine-phosphate synthase	CP000029.1:1741077-1741718	0.336673	0.217464	0.324887	-
SE1213	mreC	Cell shape-determining protein MreC	CP000029.1:1249317-1250156	0.364681	0.220363	0.328987	-
SE1774	SERP1774	Putative arginine uptake porter ArgW	CP000029.1:1817337-1818710	0.358532	0.220759	0.329344	-
SE1426	map	methionine aminopeptidase	CP000029.1:1487661-1488416	0.894966	0.221906	0.330822	-
SE0285	SERP0285	Na+/H+ ion antiporter family protein	CP000029.1:288587-289069	0.894832	0.222166	0.330975	-
SE0031	SERP0031	-	CP000029.1:25560-25652	-0.99057	0.225712	0.333667	-
SE0157	SERP0157	transcriptional regulator, GntR family	CP000029.1:160782-162146	-1.99057	0.225712	0.333667	-
SE0302	SERP0302	-	CP000029.1:303515-303664	2.593512	0.225712	0.333667	-
SE0711	SERP0711	-	CP000029.1:708783-708902	-1.99057	0.225712	0.333667	-
SE0894	SERP0894	-	CP000029.1:905415-905540	-1.99057	0.225712	0.333667	-
SE0993	murG	UDP-N-acetylglucosamine--N-acetylmuramyl-(pentapep	CP000029.1:1010596-1011669	0.892866	0.224335	0.333667	-
SE1102	gcvT	glycine cleavage system T protein	CP000029.1:1145700-1146791	0.890138	0.225655	0.333667	-
SE1214	SERP1214	-	CP000029.1:1250563-1250703	2.593512	0.225712	0.333667	-
SE1751	SERP1751	-	CP000029.1:1789160-1789387	2.593512	0.225712	0.333667	-
SE2338	SERP2338	Pyoverdine sidechain peptide synthetase III, L-Thr-L-Se	CP000029.1:2376393-2383556	0.381873	0.224907	0.333667	-
SE2386	SERP2386	cobalamin synthesis protein/P47K family protein	CP000029.1:2436232-2437113	-1.99057	0.225712	0.333667	-
SE0486	SERP0486	-	CP000029.1:479929-480021	0.149966	0.226242	0.334218	-
SE2007	gpm	2,3-bisphosphoglycerate-dependent phosphoglycerate m	CP000029.1:2023860-2024546	0.384392	0.226417	0.334242	-
SE1818	rpsN-2	30S ribosomal protein S14 type Z	CP000029.1:1855775-1855960	0.366827	0.226738	0.334483	-
SE1393	SERP1393	-	CP000029.1:1449223-1449378	-0.35346	0.227948	0.336032	-
SE0404	SERP0404	Uncharacterized protein	CP000029.1:405902-406216	1.083098	0.228556	0.336693	-
SE2024	lytS	sensor histidine kinase LytS	CP000029.1:2044375-2046150	0.991389	0.230553	0.339163	-
SE2124	SERP2124	-	CP000029.1:2148893-2149357	0.350927	0.230404	0.339163	-
SE0110	SERP0110	transcriptional regulator, LysR family	CP000029.1:98558-99379	0.009118	0.230971	0.339304	-
SE2088	SERP2088	Uncharacterized protein	CP000029.1:2115446-2116255	1.289189	0.230971	0.339304	-
SE0401	SERP0401	vibriobactin and enterobactin ABC transporter, permeas	CP000029.1:402900-403853	0.998762	0.231591	0.339978	-
SE0890	SERP0890	-	CP000029.1:902714-902902	1.035575	0.231892	0.340184	-
SE1657	SERP1657	conserved hypothetical protein	CP000029.1:1694970-1695605	-0.18355	0.232679	0.341101	-
SEA0008	SEA0008	ISSod8 transposase, TnpA_ISSod8_1	CP000028.1:6361-7035	-0.25388	0.235318	0.344731	-

SE1246	thrS	Threonine--tRNA ligase	CP000029.1:1281592-1283529	0.377411	0.237008	0.346966	-
SE1731	murAB	UDP-N-acetylglucosamine 1-carboxyvinyltransferase 2	CP000029.1:1769457-1770716	0.878308	0.238118	0.348348	-
SE1304	SERP1304	Uncharacterized protein	CP000029.1:1354546-1354857	0.942465	0.238291	0.348361	-
SE1117	pbp3	penicillin-binding protein	CP000029.1:1155781-1157871	0.382445	0.238621	0.348361	-
SE2252	sepA	neutral protease	CP000029.1:2280162-2281685	0.323974	0.238521	0.348361	-
SE0220	SERP0220	acetyl-CoA acetyltransferase	CP000029.1:230043-231191	2.331177	0.2392	0.348722	-
SE0464	SERP0464	-	CP000029.1:466549-466674	2.331177	0.2392	0.348722	-
SE0262	argS	arginyl-tRNA synthetase	CP000029.1:269127-270788	0.377168	0.240424	0.350265	-
SE1694	SERP1694	Copper-sensing transcriptional repressor RicR	CP000029.1:1737224-1737517	1.282102	0.240748	0.350495	-
SE1821	rplN	50S ribosomal protein L14	CP000029.1:1856907-1857275	0.392467	0.242874	0.353214	-
SE1961	SERP1961	Uncharacterized protein	CP000029.1:1980691-1981230	1.231525	0.242949	0.353214	-
SE1819	rplE	50S ribosomal protein L5	CP000029.1:1855983-1856522	0.394167	0.243888	0.354334	-
SE1169	SERP1169	acetyl-CoA carboxylase, biotin carboxylase	CP000029.1:1204221-1205582	0.388648	0.246989	0.358593	-
SE0340	SERP0340	Uncharacterized protein	CP000029.1:341329-342963	0.903358	0.248395	0.360386	-
SE0268	SERP0268	hydrolase, haloacid dehalogenase-like family	CP000029.1:275086-275814	0.337877	0.249018	0.361042	-
SE2063	SERP2063	Serine protease family S33, putative	CP000029.1:2088273-2089148	1.424102	0.250097	0.362357	-
SE0313	SERP0313	sensor histidine kinase	CP000029.1:316151-317191	0.894278	0.250368	0.362501	-
SE0084	nuoF	NADH dehydrogenase, subunit 5	CP000029.1:70144-71640	0.344965	0.25138	0.36363	-
SE0160	SERP0160	ISChy2, transposase	CP000029.1:164286-165299	Inf (+)	0.253731	0.36363	-
SE0271	SERP0271	-	CP000029.1:277340-277462	Inf (-)	0.253731	0.36363	-
SE0517	SERP0517	-	CP000029.1:503011-503157	Inf (+)	0.253731	0.36363	-
SE0600	SERP0600	ISCps10, transposase	CP000029.1:592507-592878	Inf (+)	0.253731	0.36363	-
SE0623	SERP0623	-	CP000029.1:616295-616402	Inf (-)	0.253731	0.36363	-
SE0810	SERP0810	-	CP000029.1:813903-814067	Inf (+)	0.253731	0.36363	-
SE1230	SERP1230	Putative membrane protein AbrB	CP000029.1:1264939-1266006	0.90065	0.252077	0.36363	-
SE1659	SERP1659	-	CP000029.1:1698060-1698167	Inf (+)	0.253731	0.36363	-
SE2130	SERP2130	-	CP000029.1:2155285-2155578	0.058038	0.252277	0.36363	-
SE2139	SERP2139	-	CP000029.1:2166003-2166107	Inf (+)	0.253731	0.36363	-
SE2266	SERP2266	-	CP000029.1:2300851-2301039	Inf (+)	0.253731	0.36363	-
SE2384	SERP2384	Baeyer-Villiger flavin-containing monooxygenase	CP000029.1:2433780-2434886	Inf (+)	0.253731	0.36363	-
SE2465	SERP2465	-	CP000029.1:2518785-2519564	Inf (-)	0.253731	0.36363	-
SERP1217	radC	UPF0758 protein LMOf2365_1569	CP000029.1:1251680-1251826	Inf (+)	0.253731	0.36363	-
SE0465	SERP0465	Uncharacterized protein	CP000029.1:466664-467605	0.393021	0.25401	0.363782	-
SE0899	thrB	homoserine kinase	CP000029.1:910133-911053	0.874575	0.255087	0.364855	-
SE2532	yycH	YycH protein	CP000029.1:2587910-2589247	0.871443	0.255104	0.364855	-
SE0203	SERP0203	deoxynucleoside kinase family protein	CP000029.1:213090-213707	1.129395	0.257543	0.367596	-
SE1869	ureA	urease, gamma subunit	CP000029.1:1894860-1895162	1.523649	0.257411	0.367596	-
SE2106	capC	-	CP000029.1:2130360-2130812	1.523649	0.257411	0.367596	-
SE0410	pepT	peptidase T	CP000029.1:410782-412020	0.396703	0.259539	0.370194	-
SE1816	rplF	50S ribosomal protein L6	CP000029.1:1854785-1855321	0.402453	0.261024	0.372061	-
SE1455	ppaC	inorganic pyrophosphatase, manganese-dependent	CP000029.1:1520522-1521451	0.403977	0.264064	0.37614	-
SE2223	SERP2223	-	CP000029.1:2259315-2259416	0.065666	0.265924	0.378533	-
SE0241	SERP0241	conserved hypothetical protein	CP000029.1:248326-248667	1.387548	0.266732	0.379428	-
SE0535	mnhD	Na(+)/H(+) antiporter subunit D	CP000029.1:518268-519764	0.861104	0.270211	0.384119	-
SE1707	SERP1707	conserved hypothetical protein	CP000029.1:1748527-1748760	0.932476	0.27073	0.384597	-
SE1796	lacR	Lactose phosphotransferase system repressor	CP000029.1:1841734-1842489	0.306777	0.270928	0.384619	-
SE0437	SERP0437	-	CP000029.1:442037-442228	0.310275	0.271331	0.38485	-
SE0733	murI	Glutamate racemase	CP000029.1:733577-734380	0.393681	0.271455	0.38485	-
SE1733	SERP1733	conserved hypothetical protein	CP000029.1:1772157-1772678	0.343513	0.272339	0.385845	-
SE0266	SERP0266	Iron compound ABC transporter, substrate-binding prot	CP000029.1:272919-273806	0.94844	0.273364	0.386777	-
SE1714	atpE	ATP synthase F0, C subunit	CP000029.1:1754404-1754616	0.882141	0.273246	0.386777	-
SE0107	SERP0107	HTH-type transcriptional regulator GltC	CP000029.1:91276-92157	0.879104	0.273736	0.387044	-
SE2014	bcr	drug resistance transporter, Bcr/CflA family	CP000029.1:2031613-2032812	0.387087	0.275782	0.389676	-
SE0215	nagB	Glucosamine-6-phosphate deaminase	CP000029.1:225758-226489	0.251974	0.277056	0.391214	-
SE0172	SERP0172	Uncharacterized protein	CP000029.1:179081-179830	0.868776	0.278235	0.392354	-
SE1226	SERP1226	Type 4 prepilin-like proteins leader peptide-processing	CP000029.1:1259069-1259779	1.401489	0.278164	0.392354	-
SE0006	SERP0006	stage 0 sporulation protein J, putative	CP000029.1:5421-6260	0.905859	0.280302	0.395005	-
SE1764	SERP1764	Cyclic di-AMP synthase CdaA	CP000029.1:1801790-1802599	0.380431	0.280644	0.395223	-
SE0920	SERP0920	Glycine betaine transporter, putative	CP000029.1:930290-931930	0.413115	0.283347	0.398764	-
SE0682	pdhC	pyruvate dehydrogenase complex E2 component, dihyd	CP000029.1:677732-679033	0.852485	0.284644	0.400323	-
SE0187	rpsG	30S ribosomal protein S7	CP000029.1:194398-194868	0.853223	0.285523	0.40129	-
SE0193	SERP0193	-	CP000029.1:202140-202349	-0.28033	0.286221	0.401737	-
SE0534	mnhE	Na+/H+ ion antiporter family protein	CP000029.1:517787-518266	0.868588	0.286045	0.401737	-
SE0688	potC	spermidine/putrescine ABC transporter, permease prote	CP000029.1:683610-684422	0.90552	0.289	0.405368	-
SE0970	lysA	Diaminopimelate decarboxylase	CP000029.1:988563-989828	0.854467	0.290422	0.407093	-
SE0834	SERP0834	conserved hypothetical protein	CP000029.1:842220-842504	1.090042	0.290669	0.407169	-
SE1457	SERP1457	conserved hypothetical protein	CP000029.1:1523242-1524270	0.859125	0.291189	0.407627	-
SE1953	SERP1953	DNA-binding response regulator	CP000029.1:1968390-1969064	0.338403	0.291942	0.40841	-
SE2367	SERP2367	major facilitator family transporter	CP000029.1:2415142-2416332	0.235876	0.294788	0.412118	-
SE0424	SERP0424	-	CP000029.1:427026-427259	0.279751	0.297168	0.41517	-
SE1661	SERP1661	tRNA N6-adenosine threonylcarbamoyltransferase	CP000029.1:1699928-1700950	0.872492	0.297966	0.41601	-
SE0853	SERP0853	Ribonuclease Y	CP000029.1:864062-865621	0.847971	0.298358	0.416282	-
SE0253	SERP0253	conserved hypothetical protein	CP000029.1:260788-261975	0.41059	0.299041	0.416604	-
SE0799	rnc	Ribonuclease 3	CP000029.1:798590-799327	0.174192	0.299181	0.416604	-
SE1905	SERP1905	-	CP000029.1:1926117-1926302	0.174192	0.299181	0.416604	-
SE1174	greA	transcription elongation factor GreA	CP000029.1:1209059-1209535	0.874857	0.29996	0.417413	-

SE0776	gmk	Guanylate kinase	CP000029.1:775250-775873	1.059693	0.30627	0.425913	-
SE1964	SERP1964	conserved hypothetical protein	CP000029.1:1982990-1983406	0.389303	0.306699	0.426229	-
SE0020	SERP0020	efflux ABC transporter, permease protein	CP000029.1:16121-18001	0.252361	0.307313	0.426613	-
SE1881	nhaC	Na+/H+ antiporter NhaC	CP000029.1:1905653-1907050	0.843391	0.307379	0.426613	-
SE0002	rnpA	ribonuclease P protein component	CP000029.1:836-1183	0.212625	0.30792	0.426968	-
SE0694	SERP0694	Inositol monophosphatase family protein, putative	CP000029.1:690030-690851	0.412956	0.30804	0.426968	-
SE1231	hemL-1	Glutamate-1-semialdehyde 2,1-aminomutase 1	CP000029.1:1266296-1267579	0.844274	0.308725	0.427636	-
SE0074	SERP0074	-	CP000029.1:63328-63945	0.41798	0.31028	0.429509	-
SE1940	SERP1940	-	CP000029.1:1955901-1956290	0.318774	0.312065	0.431697	-
SE1297	SERP1297	chorismate mutase/phospho-2-dehydro-3-deoxyheptona	CP000029.1:1344678-1345769	0.388948	0.314228	0.434404	-
SE1399	SERP1399	Uncharacterized protein	CP000029.1:1463472-1464422	0.334067	0.314944	0.435109	-
SE1741	luxS	S-ribosylhomocysteine lyase	CP000029.1:1781118-1781588	0.401016	0.31591	0.436059	-
SE1853	moaE	Molybdopterin converting factor moa, putative	CP000029.1:1881769-1882221	-0.08399	0.316045	0.436059	-
SE1141	SERP1141	-	CP000029.1:1178304-1179014	0.408337	0.317071	0.437189	-
SE0343	SERP0343	-	CP000029.1:345338-345439	1.594091	0.319467	0.439346	-
SE0718	rpmF	50S ribosomal protein L32	CP000029.1:713016-713189	0.272124	0.31946	0.439346	-
SE0939	trpD	Anthranilate phosphoribosyltransferase	CP000029.1:956056-957051	1.594091	0.319467	0.439346	-
SE2224	SERP2224	-	CP000029.1:2259614-2259763	1.594091	0.319467	0.439346	-
SE1724	SERP1724	modification methylase, HemK family	CP000029.1:1761263-1762099	0.961778	0.320382	0.440316	-
SE2167	SERP2167	-	CP000029.1:2195581-2196504	0.210731	0.32126	0.441236	-
SE0329	SERP0329	putative arabinose exporter SetC	CP000029.1:332883-334091	0.426887	0.324654	0.445607	-
SE1280	SERP1280	aminotransferase, class V	CP000029.1:1324368-1325507	1.009101	0.325395	0.446045	-
SE1419	SERP1419	low molecular weight phosphotyrosine protein phospha	CP000029.1:1482341-1482805	0.400677	0.32529	0.446045	-
SE0957	SERP0957	phosphate ABC transporter, ATP-binding protein	CP000029.1:973640-974515	0.975911	0.325981	0.446558	-
SE2355	SERP2355	Amino acid ABC transporter, permease protein, putativ	CP000029.1:2401241-2401888	0.911126	0.326593	0.447107	-
SE0112	SERP0112	-	CP000029.1:100691-100816	0.893176	0.327661	0.448278	-
SE0860	miaB	tRNA-2-methylthio-N(6)-dimethylallyladenosine syntha	CP000029.1:871516-873060	0.863367	0.329206	0.4501	-
SE1860	modB	Molybdenum ABC transporter, permease protein, putati	CP000029.1:1886814-1887485	0.890779	0.33018	0.451139	-
SE0282	SERP0282	monovalent cation/proton antiporter, MnhA/PhaA famil	CP000029.1:286332-286757	0.426317	0.330593	0.451412	-
SE0111	SERP0111	membrane protein, putative	CP000029.1:99494-100489	0.879328	0.332027	0.453077	-
SE1813	rpmD	50S ribosomal protein L30	CP000029.1:1853672-1853854	0.412953	0.332542	0.453486	-
SE0910	SERP0910	-	CP000029.1:920030-920263	1.330953	0.333362	0.454102	-
SE1828	rplB	50S ribosomal protein L2	CP000029.1:1859633-1860466	0.83235	0.333512	0.454102	-
SE2286	SERP2286	Phosphonate ABC transporter, substrate-binding protei	CP000029.1:2326415-2327371	0.947704	0.333639	0.454102	-
SE0219	SERP0219	AMP-binding protein	CP000029.1:228679-230040	0.009032	0.335094	0.454617	-
SE1057	scpB	segregation and condensation protein B	CP000029.1:1103303-1103845	1.371616	0.335094	0.454617	-
SE1474	SERP1474	-	CP000029.1:1538530-1539261	0.43329	0.334603	0.454617	-
SE2316	SERP2316	UPF0176 protein LMOf2365_1403	CP000029.1:2354749-2355711	0.009032	0.335094	0.454617	-
SERP_Se1	rrsD	Uncharacterized protein	CP000029.1:1460720-1462273	1.371616	0.335094	0.454617	-
SE1075	SERP1075	conserved hypothetical protein	CP000029.1:1120244-1120681	0.965156	0.335804	0.455287	-
SE1933	SERP1933	conserved hypothetical protein	CP000029.1:1950722-1951282	0.388181	0.338333	0.458421	-
SE2177	betB	Betaine aldehyde dehydrogenase	CP000029.1:2208852-2210342	0.849113	0.338794	0.458752	-
SE0349	SERP0349	Deoxyribodipyrimidine photolyase	CP000029.1:349758-351131	0.374223	0.340338	0.460252	-
SE2253	SERP2253	acetylornithine deacetylase	CP000029.1:2282118-2283362	0.346127	0.340334	0.460252	-
SE1712	atpH	ATP synthase F1, delta subunit	CP000029.1:1753252-1753791	0.831467	0.340663	0.460397	-
SE2292	icaR	Probable transcriptional regulatory protein (Probably Te	CP000029.1:2333498-2334055	0.867325	0.34379	0.464325	-
SE0728	trxA	Thioredoxin	CP000029.1:727476-727790	0.401103	0.344803	0.465396	-
SE0740	SERP0740	hydrolase, haloacid dehalogenase-like family	CP000029.1:736952-737638	0.420802	0.346042	0.466769	-
SE0395	SERP0395	membrane protein	CP000029.1:396498-397373	0.850506	0.346731	0.467401	-
SE0342	norR	HTH-type transcriptional regulator MgrA	CP000029.1:344895-345338	0.854538	0.348699	0.469754	-
SE0661	SERP0661	ABC transporter, ATP-binding protein	CP000029.1:656368-657771	0.924485	0.350538	0.47193	-
SE0338	SERP0338	Transcriptional regulator, AraC family	CP000029.1:339499-339963	1.146621	0.35226	0.473042	-
SE0615	SERP0615	-	CP000029.1:610224-610409	-0.16079	0.352234	0.473042	-
SE2248	SERP2248	-	CP000029.1:2276328-2276468	-0.16079	0.352234	0.473042	-
SE2418	SERP2418	-	CP000029.1:2478690-2479100	1.387548	0.352234	0.473042	-
SE1027	papS	CCA-adding enzyme	CP000029.1:1073139-1074341	0.834796	0.352543	0.47312	-
SE1436	SERP1436	Diacylglycerol kinase	CP000029.1:1497756-1498706	0.8405	0.353426	0.474004	-
SE0296	SERP0296	Teichoic acids export ATP-binding protein TagH	CP000029.1:298225-299019	0.38239	0.354351	0.474943	-
SE0019	SERP0019	-	CP000029.1:15885-16076	-0.57598	0.355755	0.475617	-
SE1480	SERP1480	-	CP000029.1:1542759-1542854	2.179154	0.355755	0.475617	-
SE1837	SERP1837	-	CP000029.1:1866982-1867131	-0.57598	0.355755	0.475617	-
SE1894	SERP1894	Uncharacterized protein	CP000029.1:1916183-1916998	0.337717	0.355435	0.475617	-
SE0210	SERP0210	Arsenical pump membrane protein	CP000029.1:221788-223080	1.331087	0.356672	0.475638	-
SE1486	SERP1486	CAAX amino terminal protease family protein	CP000029.1:1554224-1554964	0.833622	0.356657	0.475638	-
SE1679	rsbV	Anti-sigma factor antagonist	CP000029.1:1724031-1724357	0.846767	0.356489	0.475638	-
SE2161	SERP2161	-	CP000029.1:2190298-2191176	0.422401	0.356128	0.475638	-
SE0856	SERP0856	Uncharacterized protein	CP000029.1:866996-868756	0.828613	0.358728	0.478077	-
SE0037	SERP0037	cystathionine gamma-synthase/O-acetylhomoserine thio	CP000029.1:33662-34765	1.079503	0.360272	0.479832	-
SE1809	infA	translation initiation factor IF-1	CP000029.1:1850852-1851070	0.444186	0.360514	0.479851	-
SE0028	SERP0028	-	CP000029.1:24022-24756	0.990482	0.361182	0.480135	-
SE1387	fumC	Fumarate hydratase class II	CP000029.1:1444326-1445711	0.823343	0.361071	0.480135	-
SE1775	SERP1775	ferric citrate ABC transporter membrane subunit FecD	CP000029.1:1819007-1819972	0.397554	0.362674	0.481815	-
SE0513	SERP0513	conserved hypothetical protein	CP000029.1:499995-500261	0.129359	0.364482	0.483313	-
SE0569	SERP0569	-	CP000029.1:562770-563141	0.129359	0.364482	0.483313	-
SE0960	SERP0960	Phosphate-binding protein PstS	CP000029.1:976643-977620	0.943504	0.364488	0.483313	-
SE0281	SERP0281	Na(+)/H(+) antiporter subunit A	CP000029.1:283943-286345	0.447999	0.365572	0.484143	-

SE1098	SERP1098	rhodanese-like domain protein	CP000029.1:1141555-1141941	0.844625	0.365403	0.484143	-
SE2013	SERP2013	-	CP000029.1:2030648-2031349	0.322754	0.368179	0.487289	-
SE0133	ispE	4-diphosphocytidyl-2C-methyl-D-erythritol kinase	CP000029.1:123693-124541	0.834267	0.370156	0.489299	-
SE0450	SERP0450	Ribonuclease R	CP000029.1:454724-457102	0.449714	0.370162	0.489299	-
SE1033	SERP1033	TPR domain protein	CP000029.1:1077915-1079159	0.440564	0.370625	0.489605	-
SE0725	SERP0725	cvpA family protein	CP000029.1:722554-723075	0.833525	0.372183	0.491356	-
SE0968	SERP0968	Amidohydrolase	CP000029.1:986332-987483	0.826654	0.372512	0.491484	-
SE1666	ilvB	acetolactate synthase, large subunit, biosynthetic type	CP000029.1:1704708-1706411	0.836337	0.372806	0.491564	-
SE1434	SERP1434	-	CP000029.1:1495620-1496162	0.831673	0.37335	0.491974	-
SE2131	SERP2131	Copper-exporting P-type ATPase A	CP000029.1:2155815-2158199	0.819673	0.373583	0.491974	-
SE1737	SERP1737	pantothenate kinase	CP000029.1:1776659-1777456	0.876648	0.374043	0.492272	-
SE0541	SERP0541	general stress protein 13	CP000029.1:524454-524834	0.823663	0.374475	0.492533	-
SE1086	SERP1086	conserved hypothetical protein	CP000029.1:1131246-1131608	0.411198	0.375961	0.494181	-
SE1945	SERP1945	conserved hypothetical protein	CP000029.1:1960345-1960992	1.2315	0.376638	0.494762	-
SE2015	SERP2015	-	CP000029.1:2033203-2033727	0.387612	0.377149	0.495126	-
SE0126	SERP0126	UPF0213 protein SP_1535	CP000029.1:117227-117475	2.330418	0.381173	0.498655	-
SE1356	SERP1356	conserved hypothetical protein TIGR00278	CP000029.1:1413022-1413270	-0.14289	0.381255	0.498655	-
SE1458	SERP1458	-	CP000029.1:1524506-1524643	2.330418	0.381173	0.498655	-
SE2125	SERP2125	hypothetical protein	CP000029.1:2149540-2149722	2.330418	0.381173	0.498655	-
SE2329	SERP2329	-	CP000029.1:2366772-2366873	2.330418	0.381173	0.498655	-
SE2420	SERP2420	-	CP000029.1:2479838-2480083	-1.57573	0.381173	0.498655	-
SE1076	SERP1076	dihydrolipoamide acetyltransferase	CP000029.1:1120886-1122205	0.447764	0.38257	0.499911	-
SE2281	SERP2281	-	CP000029.1:2316305-2321917	0.816037	0.382689	0.499911	-
SE0181	rplL	50S ribosomal protein L7/L12	CP000029.1:184545-184913	0.814879	0.384554	0.501807	-
SE1710	atpG	ATP synthase F1, gamma subunit	CP000029.1:1750767-1751633	0.454654	0.384616	0.501807	-
SE0474	SERP0474	-	CP000029.1:473369-473572	0.931931	0.385025	0.502031	-
SE0264	SERP0264	transcriptional activator tipA, putative	CP000029.1:271720-272472	0.265453	0.38765	0.505142	-
SE0490	SERP0490	Uncharacterized protein	CP000029.1:481981-482676	0.838478	0.390771	0.508638	-
SE0717	SERP0717	conserved hypothetical protein	CP000029.1:712384-712938	0.450777	0.390815	0.508638	-
SE2197	SERP2197	-	CP000029.1:2230118-2230867	0.918899	0.392913	0.511053	-
SE0749	SERP0749	Cell division protein DivIB	CP000029.1:746749-748140	0.812043	0.393304	0.511247	-
SE0289	SERP0289	putative transport protein, monovalent cation:proton ant	CP000029.1:290256-292295	0.810488	0.395096	0.51326	-
SE2016	SERP2016	-	CP000029.1:2033790-2034599	0.398747	0.396118	0.514271	-
SE2055	SERP2055	Phosphoglucomutase	CP000029.1:2078964-2080604	0.814011	0.399698	0.5186	-
SE0294	SERP0294	-	CP000029.1:296468-297214	0.8453	0.400072	0.518766	-
SE0738	SERP0738	-	CP000029.1:736521-736655	-0.47644	0.401742	0.519974	-
SE1203	tgt	Queuine tRNA-ribosyltransferase	CP000029.1:1242020-1243159	0.809806	0.401332	0.519974	-
SE2238	SERP2238	-	CP000029.1:2267485-2267580	1.746021	0.401742	0.519974	-
SE1256	phoP	DNA-binding response regulator PhoP	CP000029.1:1295169-1295879	0.304539	0.405	0.523869	-
SE0958	SERP0958	Phosphate transport system permease protein PstA	CP000029.1:974603-975508	0.93511	0.405557	0.524269	-
SE0981	SERP0981	glyoxylase family protein	CP000029.1:998220-999029	0.804763	0.406129	0.524686	-
SE0115	SERP0115	conserved hypothetical protein TIGR00103	CP000029.1:104039-104356	-0.12831	0.407173	0.525252	-
SE0351	SERP0351	-	CP000029.1:351447-352064	1.424241	0.407173	0.525252	-
SE1166	mtn	MTA/SAH nucleosidase	CP000029.1:1201325-1202011	0.425961	0.407313	0.525252	-
SE1736	SERP1736	Uncharacterized protein	CP000029.1:1775496-1776356	0.450545	0.412413	0.531504	-
SE1315	SERP1315	conserved hypothetical protein TIGR00275	CP000029.1:1363987-1365249	0.881993	0.413785	0.532948	-
SE0403	SERP0403	vibriobactin and enterobactin ABC transporter, periplas	CP000029.1:404708-405751	0.938714	0.422219	0.543478	-
SE0317	SERP0317	Uncharacterized protein	CP000029.1:321131-322141	0.800965	0.423379	0.543976	-
SE0378	SERP0378	urea amidolyase-related protein	CP000029.1:374374-375378	0.836422	0.423327	0.543976	-
SE2395	bioA	Adenosylmethionine-8-amino-7-oxononanoate aminotra	CP000029.1:2451723-2453078	0.28664	0.423245	0.543976	-
SE1859	modC	molybdenum ABC transporter, ATP-binding protein	CP000029.1:1886193-1886813	0.843757	0.42364	0.54398	-
SE2263	SERP2263	IspC	CP000029.1:2295783-2297750	0.460478	0.424007	0.544121	-
SE2040	SERP2040	-	CP000029.1:2060849-2061631	0.917967	0.425142	0.545246	-
SE1917	SERP1917	NADP+-dependent aldehyde reductase	CP000029.1:1937106-1937987	0.470402	0.425687	0.545613	-
SE0328	SERP0328	transcriptional regulator, LysR family	CP000029.1:331971-332825	0.464308	0.428295	0.548623	-
SE2047	ppk	polyphosphate kinase	CP000029.1:2068227-2070392	0.469866	0.428629	0.548718	-
SE1017	SERP1017	UPF0398 protein LMOf2365_1918	CP000029.1:1061972-1062538	0.461155	0.43005	0.54987	-
SE1186	SERP1186	aminotransferase, class V	CP000029.1:1221884-1223026	0.45709	0.430025	0.54987	-
SE0195	ilvE	Branched-chain-amino-acid aminotransferase	CP000029.1:204021-205097	0.467468	0.43049	0.550099	-
SE1788	SERP1788	-	CP000029.1:1833830-1834696	1.053478	0.431468	0.551016	-
SE2273	SERP2273	conserved hypothetical protein	CP000029.1:2305586-2305933	0.406691	0.432187	0.5516	-
SE0467	SERP0467	-	CP000029.1:469792-470016	0.877868	0.43246	0.551615	-
SE0530	SERP0530	comA operon protein, putative	CP000029.1:515268-515642	0.85413	0.433554	0.552677	-
SE0495	SERP0495	conserved hypothetical protein	CP000029.1:484369-485229	0.430139	0.435238	0.554488	-
SE0988	arlS	Signal transduction histidine-protein kinase ArlS	CP000029.1:1005701-1007071	0.800445	0.43575	0.554806	-
SE0125	SERP0125	conserved hypothetical protein	CP000029.1:116509-117234	0.853985	0.436749	0.555742	-
SE1805	rpoA	DNA-directed RNA polymerase, alpha subunit	CP000029.1:1848886-1849830	0.474266	0.437147	0.555914	-
SE0353	norA	Quinolone resistance norA protein, putative	CP000029.1:352474-353637	0.460399	0.437426	0.555934	-
SE1266	dnaE	DNA polymerase III, alpha subunit	CP000029.1:1308406-1311603	0.795023	0.438508	0.556638	-
SE1301	SERP1301	FtsK/SpoIIIE family protein	CP000029.1:1349405-1352914	0.79662	0.438361	0.556638	-
SE0699	SERP0699	conserved hypothetical protein	CP000029.1:696078-696233	0.009089	0.439814	0.557961	-
SE0003	trmE	tRNA modification GTPase TrmE	CP000029.1:1329-2708	0.463477	0.440844	0.558597	-
SE0779	priA	Primosomal protein N'	CP000029.1:777823-780231	0.469107	0.440712	0.558597	-
SE1298	SERP1298	-	CP000029.1:1346204-1347427	0.792421	0.441112	0.558601	-
SE2019	SERP2019	conserved hypothetical protein	CP000029.1:2038557-2040074	0.449403	0.441762	0.559088	-
SE2359	SERP2359	Uncharacterized protein	CP000029.1:2404823-2405311	0.169575	0.443645	0.561135	-

SE0482	SERP0482	nitroreductase family protein	CP000029.1:477677-478219	0.852804	0.445875	0.563618	-
SE0177	nusG	Transcription termination/antitermination protein NusG	CP000029.1:181522-182070	0.79961	0.446941	0.564289	-
SE0869	SERP0869	Hydrolase, alpha/beta fold family domain protein	CP000029.1:884189-885115	0.826234	0.4468	0.564289	-
SE0850	pgsA	CDP-diacylglycerol--glycerol-3-phosphate 3-phosphatid	CP000029.1:860437-861018	0.814905	0.447286	0.564388	-
SE1864	SERP1864	Biotin transporter BioY	CP000029.1:1890197-1890745	0.366021	0.448044	0.565006	-
SE1255	phoR	sensory box histidine kinase PhoR	CP000029.1:1293472-1295169	0.841537	0.450669	0.567978	-
SE2308	SERP2308	conserved hypothetical protein	CP000029.1:2347487-2347903	1.179054	0.451618	0.568834	-
SE1339	SERP1339	Putative fluoride ion transporter CrcB	CP000029.1:1396901-1397254	0.918335	0.452409	0.569491	-
SE0572	oppD	oligopeptide ABC transporter, ATP-binding protein	CP000029.1:565394-566473	0.815577	0.453202	0.569544	-
SE0905	rpsN-1	30S ribosomal protein S14	CP000029.1:916473-916742	0.958486	0.453711	0.569544	-
SE0927	SERP0927	Sodium:alanine symporter family protein, putative	CP000029.1:941660-943117	0.470197	0.453791	0.569544	-
SE0949	SERP0949	vibriobactin and enterobactin ABC transporter, periplas	CP000029.1:965895-966905	0.231516	0.453801	0.569544	-
SE1406	mutY	Adenine DNA glycosylase	CP000029.1:1470441-1471484	0.231516	0.453801	0.569544	-
SE1759	SERP1759	Uncharacterized protein	CP000029.1:1795685-1796554	0.471624	0.454158	0.569653	-
SE0212	SERP0212	GTP cyclohydrolase FolE2	CP000029.1:223538-224416	0.812472	0.457292	0.573244	-
SE0523	SERP0523	conserved hypothetical protein	CP000029.1:507965-508285	1.063565	0.457819	0.573564	-
SE1916	SERP1916	Uncharacterized protein	CP000029.1:1936208-1936819	0.476827	0.459877	0.575801	-
SE2070	SERP2070	Regulatory protein TENI	CP000029.1:2096555-2097013	-0.40561	0.461168	0.577075	-
SE0640	SERP0640	transcription antiterminator, LytR family	CP000029.1:633418-634656	0.81014	0.467237	0.584323	-
SE0009	SERP0009	-	CP000029.1:6661-7020	0.315761	0.467705	0.584406	-
SE0624	SERP0624	-	CP000029.1:616775-616990	0.36863	0.468292	0.584406	-
SE0769	carB	Carbamoyl-phosphate synthase large chain	CP000029.1:767528-770701	0.485159	0.46812	0.584406	-
SE0836	infB	Translation initiation factor IF-2	CP000029.1:842826-844988	0.783162	0.468411	0.584406	-
SE2039	SERP2039	-	CP000029.1:2060538-2060720	0.908161	0.468717	0.584443	-
SE1175	udk	Uridine kinase	CP000029.1:1209566-1210189	0.795483	0.471556	0.587636	-
SE0010	SERP0010	-	CP000029.1:7036-7431	1.009053	0.474229	0.59027	-
SE0130	SERP0130	Ribonuclease M5	CP000029.1:121501-122046	1.009053	0.474229	0.59027	-
SE1695	cls-2	Cardiolipin synthase	CP000029.1:1737709-1739175	0.471496	0.477297	0.593738	-
SE1153	rpsT	ribosomal protein S20	CP000029.1:1191458-1191709	0.451945	0.478566	0.594966	-
SE0316	SERP0316	conserved hypothetical protein	CP000029.1:320499-321116	0.484725	0.47956	0.595851	-
SE0101	SERP0101	glycosyl transferase, group 1 family protein	CP000029.1:86000-87505	0.881338	0.481819	0.598305	-
SE2046	SERP2046	Ppx/GppA phosphatase family protein	CP000029.1:2066632-2068164	0.787442	0.482267	0.59851	-
SE0176	secE	preprotein translocase, SecE subunit	CP000029.1:181329-181511	1.009101	0.483809	0.59967	-
SE1096	SERP1096	-	CP000029.1:1140306-1140524	1.108642	0.483743	0.59967	-
SE1858	SERP1858	Uncharacterized protein	CP000029.1:1885116-1886117	0.806214	0.484054	0.59967	-
SE1212	SERP1212	Rod shape-determining protein MreD	CP000029.1:1248796-1249317	0.468078	0.489225	0.60572	-
SE1999	SERP1999	conserved hypothetical protein	CP000029.1:2017873-2018469	0.81081	0.49238	0.60927	-
SE0278	SERP0278	-	CP000029.1:282518-282664	Inf (-)	0.502488	0.612797	-
SE0356	SERP0356	-	CP000029.1:355125-355223	Inf (+)	0.502488	0.612797	-
SE0381	SERP0381	-	CP000029.1:379917-380024	Inf (+)	0.502488	0.612797	-
SE0393	SERP0393	-	CP000029.1:395687-395782	Inf (+)	0.502488	0.612797	-
SE0406	SERP0406	-	CP000029.1:407287-407814	0.009164	0.499675	0.612797	-
SE0502	SERP0502	-	CP000029.1:491547-491771	Inf (-)	0.502488	0.612797	-
SE0931	msrA-1	peptide methionine sulfoxide reductase	CP000029.1:948493-949008	0.454507	0.498095	0.612797	-
SE0973	SERP0973	-	CP000029.1:990960-991268	Inf (+)	0.502488	0.612797	-
SE1052	SERP1052	-	CP000029.1:1098520-1098624	Inf (-)	0.502488	0.612797	-
SE1130	SERP1130	Transcriptional repressor CcpN	CP000029.1:1169972-1170595	0.832844	0.497555	0.612797	-
SE1314	SERP1314	-	CP000029.1:1363803-1363901	Inf (-)	0.502488	0.612797	-
SE1319	SERP1319	permease, putative	CP000029.1:1380147-1381361	0.788411	0.497019	0.612797	-
SE1496	SERP1496	-	CP000029.1:1563810-1563902	Inf (+)	0.502488	0.612797	-
SE1738	SERP1738	-	CP000029.1:1777677-1778342	0.481851	0.49789	0.612797	-
SE1823	rpmC	50S ribosomal protein L29	CP000029.1:1857594-1857803	0.474099	0.497429	0.612797	-
SE1923	SERP1923	-	CP000029.1:1942565-1942750	Inf (+)	0.502488	0.612797	-
SE1991	SERP1991	-	CP000029.1:2011744-2011848	Inf (-)	0.502488	0.612797	-
SE2078	SERP2078	-	CP000029.1:2105085-2105213	Inf (+)	0.502488	0.612797	-
SE2135	SERP2135	-	CP000029.1:2160907-2161047	Inf (-)	0.502488	0.612797	-
SE2243	SERP2243	-	CP000029.1:2271304-2271429	Inf (-)	0.502488	0.612797	-
SE2298	SERP2298	-	CP000029.1:2339963-2340103	Inf (-)	0.502488	0.612797	-
SE2301	hisF	Imidazole glycerol phosphate synthase subunit hisF, put	CP000029.1:2341841-2342599	Inf (+)	0.502488	0.612797	-
SE2350	SERP2350	-	CP000029.1:2395767-2395991	Inf (-)	0.502488	0.612797	-
SE2376	SERP2376	-	CP000029.1:2424381-2424482	Inf (+)	0.502488	0.612797	-
SERP_Se1	rrsB	Uncharacterized protein	CP000029.1:150374-151927	Inf (-)	0.502488	0.612797	-
SE2331	rarD	rarD protein	CP000029.1:2368097-2369002	0.488151	0.502922	0.612973	-
SE1922	SERP1922	-	CP000029.1:1942408-1942587	1.146571	0.504731	0.614495	-
SE2531	yycI	yycI protein	CP000029.1:2587118-2587909	0.775191	0.504754	0.614495	-
SE0806	trmD	tRNA (guanine-N(1)-)-methyltransferase	CP000029.1:807190-807927	0.862706	0.505614	0.615101	-
SE0864	hexB	DNA mismatch repair protein MutL	CP000029.1:876779-878716	0.77532	0.505834	0.615101	-
SE0731	SERP0731	succinate dehydrogenase, flavoprotein subunit	CP000029.1:730806-732572	0.497616	0.506374	0.615403	-
SE1328	ribD	Riboflavin biosynthesis protein RibD	CP000029.1:1388743-1389786	0.491344	0.506937	0.615733	-
SE0409	SERP0409	glycerate kinase 2	CP000029.1:409561-410691	0.493522	0.510382	0.619561	-
SE0832	SERP0832	Ribosome maturation factor RimP	CP000029.1:840491-840958	0.39612	0.511556	0.620288	-
SE2272	SERP2272	peptide methionine sulfoxide reductase MsrA	CP000029.1:2304937-2305416	0.835068	0.511569	0.620288	-
SE0562	SERP0562	conserved hypothetical protein	CP000029.1:552943-553251	0.428321	0.512877	0.621517	-
SE2254	SERP2254	-	CP000029.1:2283725-2284201	1.816526	0.517394	0.626631	-
SE1824	rplP	50S ribosomal protein L16	CP000029.1:1857793-1858227	0.767289	0.51844	0.627538	-
SE0966	dapB	4-hydroxy-tetrahydrodipicolinate reductase	CP000029.1:984709-985431	0.500827	0.522223	0.631755	-

SE0254	SERP0254	conserved hypothetical protein	CP000029.1:261972-262748	0.481041	0.522997	0.632329	-
SE0712	SERP0712	UPF0298 protein LMOf2365_2085	CP000029.1:708974-709225	0.009133	0.523805	0.632943	-
SE1692	SERP1692	-	CP000029.1:1736728-1736865	0.465616	0.525832	0.635029	-
SE1338	SERP1338	crcB protein	CP000029.1:1396539-1396904	0.947706	0.526363	0.635308	-
SE0292	sitA	metal ABC transporter, ATP-binding protein	CP000029.1:294793-295539	0.761544	0.533002	0.642953	-
SE2166	fdaB	Fructose-bisphosphate aldolase, class I	CP000029.1:2194558-2195448	0.760235	0.535814	0.645976	-
SE0057	SERP0057	lipoprotein, putative	CP000029.1:45446-46069	1.009114	0.537425	0.647178	-
SE2315	drp35	Lactonase drp35	CP000029.1:2353490-2354467	0.826711	0.537161	0.647178	-
SE0631	SERP0631	Putative 2-succinyl-6-hydroxy-2,4-cyclohexadiene-1-ca	CP000029.1:622550-623353	0.761651	0.541207	0.651135	-
SE2000	SERP2000	-	CP000029.1:2018493-2018855	0.472408	0.541327	0.651135	-
SE1456	aldA-1	Aldehyde dehydrogenase	CP000029.1:1521751-1523130	0.771384	0.542874	0.652623	-
SE0964	asd	aspartate-semialdehyde dehydrogenase	CP000029.1:982834-983823	0.500439	0.543546	0.65306	-
SE0938	trpG	Anthranilate synthase component II, putative	CP000029.1:955487-956053	0.134607	0.545091	0.654543	-
SE2357	SERP2357	Amino acid ABC transporter, permease protein, putativ	CP000029.1:2402869-2403504	0.331068	0.546645	0.656037	-
SE2553	dnaA	Chromosomal replication initiator protein DnaA	CP000029.1:2614976-2616331	0.757605	0.552314	0.662463	-
SE1857	moaB	Molybdenum cofactor biosynthesis protein B	CP000029.1:1884565-1885095	0.497271	0.555585	0.666008	-
SE0588	SERP0588	Uncharacterized protein	CP000029.1:580800-581654	0.844484	0.556057	0.666195	-
SE0579	SERP0579	conserved hypothetical protein	CP000029.1:573537-574517	0.449673	0.556544	0.666401	-
SE1088	accB	oxaloacetate decarboxylase, alpha subunit	CP000029.1:1132978-1133445	0.87981	0.557509	0.667178	-
SE2151	panC	pantoate--beta-alanine ligase	CP000029.1:2177826-2178686	0.7713	0.557975	0.667357	-
SE0630	menD	2-succinyl-6-hydroxy-2,4-cyclohexadiene-1-carboxylic	CP000029.1:620890-622563	0.755023	0.559412	0.668697	-
SE2548	gyrA	DNA gyrase subunit A	CP000029.1:2607094-2609775	0.753391	0.56108	0.670311	-
SE0432	trxB	thioredoxin reductase	CP000029.1:436779-437711	0.509242	0.562051	0.671091	-
SE0174	SERP0174	-	CP000029.1:180424-180999	0.75756	0.563866	0.672878	-
SE2025	lytR	response regulator LytR	CP000029.1:2046131-2046889	0.78774	0.564595	0.673368	-
SE1062	SERP1062	mutT/nudix family protein	CP000029.1:1106728-1107270	0.782824	0.569664	0.679029	-
SE2393	bioW	6-carboxyhexanoate--CoA ligase	CP000029.1:2449900-2450586	0.284737	0.570099	0.679165	-
SE0790	rpmB	ribosomal protein L28	CP000029.1:789876-790064	0.516574	0.57256	0.681711	-
SE1825	rpsC	30S ribosomal protein S3	CP000029.1:1858230-1858883	0.518255	0.573905	0.682928	-
SE0898	thrC	Threonine synthase	CP000029.1:909067-910131	0.757367	0.580689	0.690611	-
SE1808	rpmJ	ribosomal protein L36	CP000029.1:1850707-1850820	1.087083	0.581089	0.690697	-
SE1804	rplQ	50S ribosomal protein L17	CP000029.1:1848501-1848869	0.520727	0.582522	0.692011	-
SE0054	SERP0054	-	CP000029.1:44849-45079	0.490374	0.585824	0.695542	-
SE1476	SERP1476	-	CP000029.1:1540154-1540813	0.520585	0.586699	0.696189	-
SE1044	SERP1044	ribosomal protein S1	CP000029.1:1089517-1090695	0.522033	0.587656	0.696934	-
SE0148	SERP0148	tRNA(Ile)-lysidine synthetase	CP000029.1:138560-139858	0.824669	0.591288	0.700841	-
SE2144	pyrD	Dihydroorotate dehydrogenase (quinone)	CP000029.1:2171689-2172753	0.50731	0.591615	0.700841	-
SE0559	SERP0559	-	CP000029.1:550250-550405	-0.21335	0.593113	0.701435	-
SE0690	SERP0690	-	CP000029.1:685561-686712	0.745186	0.592954	0.701435	-
SE1897	SERP1897	-	CP000029.1:1919092-1919427	-0.21335	0.593113	0.701435	-
SE0573	oppF	oligopeptide ABC transporter, ATP-binding protein	CP000029.1:566466-567404	0.755981	0.595693	0.704092	-
SE1383	SERP1383	-	CP000029.1:1440498-1440962	0.921651	0.600301	0.709141	-
SE0956	SERP0956	Phosphate-specific transport system accessory protein P	CP000029.1:972986-973633	0.888791	0.601004	0.709574	-
SE1705	fabZ	(3R)-hydroxymyristoyl-(acyl-carrier-protein) dehydrata	CP000029.1:1746681-1747118	0.742804	0.602312	0.710722	-
SE0217	SERP0217	SIS domain protein	CP000029.1:227245-227793	0.527559	0.604568	0.712985	-
SE2140	SERP2140	conserved hypothetical protein	CP000029.1:2166137-2167561	0.769911	0.605783	0.714019	-
SE1305	pepA	peptidase, M42 family	CP000029.1:1354863-1355948	0.521804	0.607435	0.715567	-
SE0012	SERP0012	prophage LambdaBa04, DNA-binding protein	CP000029.1:8156-8725	1.079445	0.610546	0.71883	-
SE1108	SERP1108	comG operon protein 2	CP000029.1:1149143-1150210	0.343524	0.617782	0.726944	-
SE0701	SERP0701	conserved hypothetical protein	CP000029.1:696862-697137	0.526249	0.618806	0.727743	-
SE0982	SERP0982	-	CP000029.1:999229-999402	1.146489	0.620334	0.728732	-
SE2421	SERP2421	Uncharacterized protein	CP000029.1:2480169-2481056	0.75569	0.620337	0.728732	-
SE0715	coaD	phosphopantetheine adenylyltransferase	CP000029.1:710235-710720	0.498484	0.621787	0.730029	-
SE1155	SERP1155	DNA internalization-related competence protein ComE	CP000029.1:1192846-1195062	0.481463	0.625036	0.733435	-
SE0151	hslO	chaperonin, 33 kDa	CP000029.1:143029-143910	0.769266	0.626116	0.733888	-
SE0170	cysS	Cysteine--tRNA ligase	CP000029.1:177282-178682	0.530615	0.625837	0.733888	-
SE0051	SERP0051	Uncharacterized membrane protein YrhP	CP000029.1:43190-43822	2.0084	0.628704	0.735287	-
SE1479	SERP1479	-	CP000029.1:1542402-1542572	2.0084	0.628704	0.735287	-
SE1880	SERP1880	Putative secreted antigen GbpB/SagA putative peptidog	CP000029.1:1904431-1905204	2.0084	0.628704	0.735287	-
SE2549	gyrB	DNA gyrase subunit B	CP000029.1:2609812-2611743	0.531269	0.628497	0.735287	-
SE1484	groEL	chaperonin, 60 kDa	CP000029.1:1552090-1553709	0.534138	0.62969	0.736032	-
SE0004	gidA	glucose-inhibited division protein A	CP000029.1:2781-4658	0.737707	0.63112	0.737296	-
SE2117	SERP2117	conserved hypothetical protein	CP000029.1:2142373-2142765	0.851551	0.631629	0.737483	-
SE0839	ribF	riboflavin biosynthesis protein RibF	CP000029.1:846712-847683	0.760186	0.636064	0.742251	-
SE0674	SERP0674	potassium uptake protein, TrkA family	CP000029.1:670003-670662	0.507272	0.63789	0.74397	-
SE1750	SERP1750	-	CP000029.1:1787551-1788927	0.488571	0.638974	0.744823	-
SE1161	nadD	Probable nicotinate-nucleotide adenylyltransferase	CP000029.1:1197987-1198562	0.741547	0.639669	0.745221	-
SE0417	SERP0417	comF operon protein 1	CP000029.1:417958-419121	0.785058	0.643075	0.748776	-
SE1160	SERP1160	conserved hypothetical protein TIGR00488	CP000029.1:1197416-1198000	0.517038	0.64369	0.749079	-
SE1872	ureE	Urease accessory protein UreE	CP000029.1:1897309-1897761	0.524074	0.645746	0.751057	-
SE1949	tcaR	-	CP000029.1:1964658-1965113	0.952513	0.646181	0.75115	-
SE1236	hemA	glutamyl-tRNA reductase	CP000029.1:1271095-1272441	0.538891	0.648072	0.752934	-
SE0587	SERP0587	inorganic polyphosphate/ATP-NAD kinase, putative	CP000029.1:579994-580803	0.729467	0.653019	0.75743	-
SE0680	pdhA	pyruvate dehydrogenase complex E1 component, alpha	CP000029.1:675508-676620	0.72583	0.652518	0.75743	-
SE1713	atpF	ATP synthase F0, B subunit	CP000029.1:1753791-1754306	0.727581	0.652716	0.75743	-
SE1237	SERP1237	GTP-binding protein	CP000029.1:1272611-1273198	0.473767	0.659944	0.765042	-

SE0041	SERP0041	GTP-binding protein YchF	CP000029.1:37608-38705	0.538503	0.660781	0.765592	-
SE0373	SERP0373	7-cyano-7-deazaguanine synthase	CP000029.1:370075-370746	1.231564	0.662502	0.765905	-
SE1145	SERP1145	Ribosomal RNA small subunit methyltransferase E	CP000029.1:1181514-1182275	1.231564	0.662502	0.765905	-
SE1382	SERP1382	conserved hypothetical protein	CP000029.1:1439186-1440316	1.231564	0.662502	0.765905	-
SE1664	SERP1664	conserved hypothetical protein TIGR00150	CP000029.1:1702024-1702485	1.231564	0.662502	0.765905	-
SE1876	sarR	Transcriptional regulator SarA	CP000029.1:1900011-1900355	0.522007	0.663359	0.766476	-
SE0167	SERP0167	conserved hypothetical protein	CP000029.1:173355-174431	0.739564	0.66485	0.767358	-
SE0708	SERP0708	conserved hypothetical protein	CP000029.1:705805-706857	0.517098	0.664608	0.767358	-
SE0411	SERP0411	putative membrane protein	CP000029.1:412041-412535	0.760786	0.665787	0.76802	-
SE0533	mnhF	monovalent cation/proton antiporter subunit, putative	CP000029.1:517494-517787	0.7479	0.669228	0.771568	-
SE0370	SERP0370	-	CP000029.1:368235-368834	0.730273	0.67084	0.772583	-
SE1336	tal	transaldolase, putative	CP000029.1:1395204-1395917	0.728636	0.670502	0.772583	-
SE0069	guaB	inosine-5'-monophosphate dehydrogenase	CP000029.1:57085-58551	0.546399	0.67308	0.774739	-
SE0250	SERP0250	Uncharacterized protein	CP000029.1:258272-258748	0.499738	0.674452	0.775472	-
SE1035	aroB	3-dehydroquinate synthase	CP000029.1:1080474-1081538	0.728529	0.674344	0.775472	-
SE1275	ackA	acetate kinase	CP000029.1:1319186-1320439	0.546065	0.675012	0.775695	-
SE0908	SERP0908	-	CP000029.1:918967-919068	0.799173	0.67755	0.778188	-
SE0998	SERP0998	Phosphotransferase system enzyme IIA, putative	CP000029.1:1014570-1015070	0.543834	0.67809	0.778384	-
SE2287	SERP2287	conserved hypothetical protein	CP000029.1:2327606-2329156	0.535565	0.678855	0.778839	-
SE2415	SERP2415	-	CP000029.1:2476845-2476940	0.146489	0.679314	0.778942	-
SE0116	recR	Recombination protein RecR	CP000029.1:104363-104959	0.536342	0.682545	0.7816	-
SE0571	oppC	Uncharacterized protein	CP000029.1:564438-565379	0.737345	0.682743	0.7816	-
SE0629	SERP0629	Isochorismate synthase MenF	CP000029.1:619533-620903	0.542694	0.682178	0.7816	-
SE1146	prmA	ribosomal protein L11 methyltransferase	CP000029.1:1182268-1183206	0.547834	0.685199	0.783987	-
SE2313	SERP2313	-	CP000029.1:2352266-2352370	0.759733	0.685733	0.784172	-
SE0267	SERP0267	ABC-type cobalamin/Fe3+-siderophores transport syste	CP000029.1:274083-275027	0.536886	0.686929	0.785115	-
SE0493	SERP0493	-	CP000029.1:483779-483949	-0.57573	0.692096	0.788885	-
SE1395	SERP1395	ABC transporter, permease protein, putative	CP000029.1:1450590-1452047	0.009235	0.692096	0.788885	-
SE2053	SERP2053	mutator mutT protein	CP000029.1:2076591-2076989	-0.57573	0.692096	0.788885	-
SE2241	SERP2241	transcriptional regulator, MarR family	CP000029.1:2268844-2269290	0.009235	0.692096	0.788885	-
SERP_Se1	rrsA	Uncharacterized protein	CP000029.1:105734-107287	-0.57573	0.692096	0.788885	-
SE0237	SERP0237	lipoate-protein ligase A, putative	CP000029.1:243586-244425	0.552152	0.692771	0.789228	-
SE1084	xseA	exodeoxyribonuclease VII, large subunit	CP000029.1:1129429-1130766	0.538721	0.696768	0.793354	-
SE2533	yycG	Two-component sensor histidine kinase (Paired with Y	CP000029.1:2589240-2591072	0.552918	0.698277	0.794643	-
SE0263	SERP0263	DNA repair protein, HhH-GPD family	CP000029.1:271030-271665	0.780832	0.698668	0.794659	-
SE2141	SERP2141	conserved hypothetical protein	CP000029.1:2167662-2168720	0.53436	0.702542	0.798206	-
SE2221	SERP2221	-	CP000029.1:2257793-2258410	0.476869	0.702204	0.798206	-
SE1282	SERP1282	conserved hypothetical protein	CP000029.1:1327666-1328136	0.520435	0.703266	0.798417	-
SE1937	fni-2	Isopentenyl-diphosphate delta-isomerase	CP000029.1:1953279-1954328	0.726544	0.703484	0.798417	-
SE1008	SERP1008	conserved hypothetical protein	CP000029.1:1021490-1021684	0.357042	0.707153	0.800858	-
SE1394	SERP1394	amino acid ABC transporter, ATP-binding protein	CP000029.1:1449875-1450597	0.357042	0.707153	0.800858	-
SE1418	SERP1418	-	CP000029.1:1481985-1482200	0.92669	0.707153	0.800858	-
SE2090	SERP2090	Uncharacterized protein	CP000029.1:2117387-2117881	0.357042	0.707153	0.800858	-
SE1162	SERP1162	conserved hypothetical protein TIGR00253	CP000029.1:1198559-1198852	0.729717	0.70759	0.800924	-
SE0159	SERP0159	Pyridoxal 5'-phosphate synthase subunit PdxT	CP000029.1:163130-163690	0.550629	0.711896	0.805189	-
SE0251	SERP0251	Uncharacterized protein	CP000029.1:258840-260138	0.717258	0.712122	0.805189	-
SE1878	sarY	-	CP000029.1:1901119-1901847	0.522363	0.714817	0.807804	-
SE0803	ffh	signal recognition particle protein	CP000029.1:804611-805978	0.557207	0.716448	0.809215	-
SE0300	tagD	glycerol-3-phosphate cytidyltransferase	CP000029.1:302776-303174	0.857118	0.717749	0.810251	-
SE2032	SERP2032	alcohol dehydrogenase, zinc-containing	CP000029.1:2052409-2053461	0.561199	0.723971	0.816838	-
SE0445	pgm	2,3-bisphosphoglycerate-independent phosphoglycerate	CP000029.1:449762-451279	0.562471	0.728035	0.820683	-
SE0757	SERP0757	Septum site-determining protein DivIVA	CP000029.1:754608-755264	0.559144	0.728546	0.820683	-
SE1844	femX	-	CP000029.1:1874093-1875343	0.707455	0.728256	0.820683	-
SE1482	SERP1482	-	CP000029.1:1544501-1550422	0.535161	0.729297	0.821091	-
SE0463	SERP0463	Uncharacterized protein	CP000029.1:465874-466377	0.52733	0.730235	0.821377	-
SE0919	mscL	Large-conductance mechanosensitive channel	CP000029.1:929728-930078	0.008979	0.731885	0.821377	-
SE0987	SERP0987	-	CP000029.1:1005308-1005418	0.008979	0.731885	0.821377	-
SE0995	SERP0995	-	CP000029.1:1012226-1012342	0.008979	0.731885	0.821377	-
SE1107	comGC	Uncharacterized protein	CP000029.1:1148808-1149125	0.008979	0.731885	0.821377	-
SE2163	SERP2163	-	CP000029.1:2192083-2192271	1.594091	0.731885	0.821377	-
SE2153	SERP2153	Putative 2-dehydropantoate 2-reductase	CP000029.1:2179573-2180442	0.525348	0.73245	0.821574	-
SE1483	SERP1483	-	CP000029.1:1550599-1551672	0.532052	0.733807	0.822659	-
SE0376	SERP0376	-	CP000029.1:373430-373636	0.52036	0.734319	0.822796	-
SE0531	SERP0531	Predicted protein	CP000029.1:515701-516855	0.48716	0.735902	0.82409	-
SE1850	moaA	Cyclic pyranopterin monophosphate synthase	CP000029.1:1879893-1880915	0.556889	0.736254	0.82409	-
SE1998	SERP1998	Activator of Hsp90 ATPase 1 family protein	CP000029.1:2017121-2017591	0.289242	0.739035	0.826763	-
SE1069	SERP1069	Uncharacterized protein	CP000029.1:1113297-1114178	0.73378	0.745355	0.833065	-
SE1168	SERP1168	LamB/YcsF family protein	CP000029.1:1203466-1204224	0.560946	0.745457	0.833065	-
SE0228	ung	uracil-DNA glycosylase	CP000029.1:236766-237416	0.55272	0.749096	0.836689	-
SE2406	SERP2406	Two-component system sensor histidine kinase/respons	CP000029.1:2466930-2467685	0.473046	0.753783	0.841478	-
SE2288	SERP2288	Escherichia coli K-12	CP000029.1:2329253-2330788	0.56689	0.755795	0.84291	-
SE2370	SERP2370	Nickel ABC transporter, permease protein, putative	CP000029.1:2417888-2418766	0.424158	0.755864	0.84291	-
SE1662	SERP1662	Ribosomal-protein-alanine acetyltransferase	CP000029.1:1700947-1701384	0.713178	0.75629	0.84294	-
SE0284	SERP0284	Na(+)/H(+) antiporter subunit D	CP000029.1:287091-288590	0.697362	0.757522	0.843868	-
SE0180	rplJ	ribosomal protein L10	CP000029.1:184007-184507	0.570218	0.757996	0.84395	-
SE2006	SERP2006	-	CP000029.1:2023570-2023674	0.008953	0.759851	0.844679	-

SE2268	SERP2268	-	CP000029.1:2301515-2301670	1.231371	0.759851	0.844679	-
SE2454	SERP2454	-	CP000029.1:2507320-2508177	0.008953	0.759851	0.844679	-
SE1127	rpoD	RNA polymerase sigma-43 factor	CP000029.1:1165795-1166901	0.5701	0.763037	0.847774	-
SE0305	SERP0305	membrane protein, putative	CP000029.1:307937-308770	0.525886	0.764461	0.84891	-
SE0184	rpoC	DNA-directed RNA polymerase subunit beta'	CP000029.1:189612-193235	0.694272	0.76681	0.850858	-
SE0967	dapD	2,3,4,5-tetrahydropyridine-2-carboxylate N-succinyltran	CP000029.1:985453-986175	0.571962	0.767021	0.850858	-
SE2152	panB	3-methyl-2-oxobutanoate hydroxymethyltransferase	CP000029.1:2178679-2179497	0.70542	0.770637	0.85442	-
SE1116	rpmG-3	50S ribosomal protein L33 2	CP000029.1:1155527-1155676	0.85712	0.776602	0.860254	-
SE1904	SERP1904	-	CP000029.1:1925764-1926120	0.570449	0.776714	0.860254	-
SE0606	ypfP	Processive diacylglycerol beta-glucosyltransferase	CP000029.1:598429-599604	0.569814	0.777676	0.860416	-
SE2008	SERP2008	Uncharacterized protein	CP000029.1:2024788-2025669	0.540628	0.777571	0.860416	-
SE0798	acpP	acyl carrier protein	CP000029.1:798242-798475	0.559462	0.778549	0.86093	-
SE0719	SERP0719	-	CP000029.1:713669-716143	0.724377	0.780128	0.861733	-
SE1414	SERP1414	conserved hypothetical protein	CP000029.1:1478533-1478694	1.179154	0.780907	0.861733	-
SE2083	SERP2083	glyoxalase family protein	CP000029.1:2109745-2110542	0.565798	0.780135	0.861733	-
SE2322	SERP2322	-	CP000029.1:2359691-2359798	1.179154	0.780907	0.861733	-
SE1323	SERP1323	Uncharacterized protein	CP000029.1:1384106-1384933	0.816454	0.782585	0.862683	-
SE1325	ribH	6,7-dimethyl-8-ribityllumazine synthase	CP000029.1:1386436-1386897	0.701815	0.782414	0.862683	-
SE0551	SERP0551	conserved hypothetical protein	CP000029.1:537549-538124	0.705362	0.783609	0.86336	-
SE0932	SERP0932	putative transcriptional regulator	CP000029.1:949144-950133	0.723189	0.786881	0.866513	-
SE0804	rpsP	30S ribosomal protein S16	CP000029.1:806285-806560	0.729711	0.788851	0.868069	-
SE1012	SERP1012	5'-3' exonuclease family protein	CP000029.1:1054324-1055202	0.699522	0.789116	0.868069	-
SE1918	SERP1918	N-acyl-L-amino acid amidohydrolase	CP000029.1:1938084-1939202	0.569749	0.791475	0.87021	-
SE0109	gltD	NADH-glutamate synthase small subunit, putative	CP000029.1:96862-98325	0.692912	0.798116	0.87596	-
SE0713	SERP0713	-	CP000029.1:709235-709624	0.557109	0.797225	0.87596	-
SE0773	SERP0773	-	CP000029.1:772548-772682	0.769911	0.798905	0.87596	-
SE1378	cbf1	3'-5' exoribonuclease YhaM	CP000029.1:1433435-1434376	0.580266	0.799194	0.87596	-
SE1703	SERP1703	single-stranded DNA-binding protein	CP000029.1:1745337-1745732	0.769911	0.798905	0.87596	-
SE1871	ureC	Urease subunit alpha	CP000029.1:1895580-1897295	0.580596	0.799095	0.87596	-
SE2529	SERP2529	Ribosomal RNA large subunit methyltransferase H	CP000029.1:2584176-2584655	0.456595	0.803068	0.879749	-
SERP_Se5	rrfG	-	CP000029.1:149424-149538	0.539622	0.806183	0.882704	-
SE1475	SERP1475	ABC transporter, ATP-binding protein	CP000029.1:1539261-1540136	0.684598	0.806734	0.882849	-
SE0872	gpxA-1	glutathione peroxidase	CP000029.1:886540-887016	0.714916	0.808125	0.883376	-
SE1802	SERP1802	Energy-coupling factor transporter ATP-binding protein	CP000029.1:1846504-1847364	0.714916	0.808125	0.883376	-
SE2530	yycJ	metallo-beta-lactamase family protein	CP000029.1:2585697-2586497	0.72454	0.808471	0.883376	-
SE1803	SERP1803	ABC transporter, ATP-binding protein	CP000029.1:1847361-1848170	0.513568	0.809307	0.883832	-
SE0124	SERP0124	conserved hypothetical protein	CP000029.1:116066-116413	0.581387	0.809979	0.884108	-
SE0027	SERP0027	ABC transporter, ATP-binding protein	CP000029.1:23740-23994	0.371716	0.81105	0.884363	-
SE1007	SERP1007	PBS lyase HEAT-like repeat domain protein	CP000029.1:1021218-1021478	0.371716	0.81105	0.884363	-
SE0166	radA	DNA repair protein RadA	CP000029.1:171946-173319	0.575299	0.813908	0.887021	-
SE0185	SERP0185	ribosomal protein L7A family	CP000029.1:193563-193823	0.685472	0.815186	0.887314	-
SE1711	atpA	ATP synthase F1, alpha subunit	CP000029.1:1751718-1753229	0.681504	0.815437	0.887314	-
SE2413	SERP2413	tRNA-dihydrouridine synthase	CP000029.1:2474977-2475957	0.718323	0.814751	0.887314	-
SE1188	SERP1188	-	CP000029.1:1224464-1224607	0.53635	0.816369	0.887869	-
SE1215	SERP1215	-	CP000029.1:1250787-1251272	0.752747	0.820746	0.89217	-
SE0360	nagA	N-acetylglucosamine-6-phosphate deacetylase	CP000029.1:359264-360436	0.563677	0.823492	0.894695	-
SE1836	topB	DNA topoisomerase III	CP000029.1:1864678-1866813	0.580054	0.824989	0.89586	-
SE0750	ftsA	Cell division protein FtsA	CP000029.1:748246-749640	0.680358	0.825626	0.896091	-
SE1401	hemL-2	Glutamate-1-semialdehyde 2,1-aminomutase 2	CP000029.1:1464964-1466253	0.680066	0.82607	0.896112	-
SE0593	SERP0593	-	CP000029.1:586848-587051	0.902154	0.832022	0.902105	-
SE0985	sucB	2-oxoglutarate dehydrogenase, E2 component, dihydrol	CP000029.1:1001204-1002466	0.591164	0.835896	0.904911	-
SE1158	SERP1158	Uncharacterized protein	CP000029.1:1196340-1197059	0.578073	0.835856	0.904911	-
SE2150	panD	aspartate 1-decarboxylase	CP000029.1:2177447-2177833	0.508676	0.835873	0.904911	-
SE1036	aroC	Chorismate synthase	CP000029.1:1081566-1082732	0.568524	0.836648	0.905261	-
SE0685	SERP0685	transcriptional regulator, Cro/CI family	CP000029.1:681163-681702	0.272047	0.840383	0.907765	-
SE1028	SERP1028	glycosyl transferase, group 1 family protein	CP000029.1:1074331-1075473	0.680864	0.840757	0.907765	-
SE1680	rsbU	SigmaB regulation protein RsbU, putative	CP000029.1:1724457-1725458	0.558427	0.841112	0.907765	-
SE1702	sceD	-	CP000029.1:1744290-1744949	0.916079	0.840383	0.907765	-
SE1907	SERP1907	hydrolase, haloacid dehalogenase-like family	CP000029.1:1926825-1927460	0.916079	0.840383	0.907765	-
SE0812	rnhB	Ribonuclease HII	CP000029.1:814956-815726	0.692915	0.842386	0.908675	-
SE0344	SERP0344	cobalamin synthesis protein/P47K family protein	CP000029.1:345560-346486	0.680307	0.846357	0.911096	-
SE1109	SERP1109	comG operon protein 1	CP000029.1:1150182-1151165	0.560525	0.84605	0.911096	-
SE1667	SERP1667	acetolactate synthase, small subunit	CP000029.1:1706411-1706644	0.543448	0.84605	0.911096	-
SE2068	SERP2068	conserved hypothetical protein	CP000029.1:2094025-2094315	0.587429	0.84562	0.911096	-
SE1826	rplV	50S ribosomal protein L22	CP000029.1:1858907-1859260	0.594214	0.848312	0.912735	-
SE0073	SERP0073	-	CP000029.1:62664-63218	0.591913	0.849992	0.914077	-
SE2401	SERP2401	-	CP000029.1:2462460-2463194	0.755339	0.851571	0.915308	-
SE2318	SERP2318	-	CP000029.1:2356576-2356968	0.456595	0.854227	0.917696	-
SE2203	SERP2203	efflux ABC transporter, permease protein	CP000029.1:2237571-2239583	0.703286	0.856017	0.919151	-
SE1000	msrA-2	peptide methionine sulfoxide reductase	CP000029.1:1015508-1016032	0.583791	0.857073	0.919817	-
SE1468	SERP1468	-	CP000029.1:1534168-1534338	0.568519	0.857912	0.92025	-
SE0275	SERP0275	efflux transporter, RND family, MFP subunit	CP000029.1:280211-281164	0.424145	0.860052	0.921336	-
SE0992	SERP0992	PAP2 family protein	CP000029.1:1009969-1010583	0.670923	0.860234	0.921336	-
SERP_Se5	rrfE	-	CP000029.1:1713943-1714057	0.424145	0.860052	0.921336	-
SE2112	SERP2112	Zinc-binding oxidoreductase, putative	CP000029.1:2135452-2136504	0.669285	0.864853	0.925814	-
SE1822	rpsQ	30S ribosomal protein S17	CP000029.1:1857306-1857569	0.670881	0.866914	0.92755	-

SE1913	SERP1913	-	CP000029.1:1933161-1933673	0.715375	0.867888	0.928121	-
SE0440	SERP0440	-	CP000029.1:444816-445052	0.449634	0.870082	0.929526	-
SE1928	SERP1928	-	CP000029.1:1948055-1948156	0.81643	0.870082	0.929526	-
SE0427	uvrA	excinuclease ABC, A subunit	CP000029.1:429809-432643	0.666875	0.873495	0.9327	-
SE0637	SERP0637	Putative acetyltransferase SAS0989	CP000029.1:631904-632326	0.768131	0.874444	0.933242	-
SE1034	aroA	3-phosphoshikimate 1-carboxyvinyltransferase	CP000029.1:1079163-1080464	0.665746	0.879356	0.937859	-
SE1078	SERP1078	3-methyl-2-oxobutanoate dehydrogenase, alpha subunit	CP000029.1:1123194-1124192	0.675287	0.879659	0.937859	-
SE0127	SERP0127	Ribosomal RNA small subunit methyltransferase I	CP000029.1:117477-118316	0.676522	0.881961	0.939364	-
SE1847	SERP1847	major facilitator family transporter	CP000029.1:1877107-1878300	0.668609	0.881763	0.939364	-
SE0439	SERP0439	-	CP000029.1:444023-444643	0.506594	0.885577	0.942264	-
SE2311	SERP2311	Uncharacterized protein	CP000029.1:2349132-2349650	0.802662	0.885577	0.942264	-
SE2275	SERP2275	Glycosyltransferase Gtf1	CP000029.1:2307372-2308880	0.667801	0.886672	0.942954	-
SE0724	SERP0724	conserved hypothetical protein	CP000029.1:722287-722553	0.698167	0.888581	0.944509	-
SE2031	SERP2031	Glycine betaine/carnitine/choline ABC transporter, ATI	CP000029.1:2050663-2051919	0.605205	0.890877	0.946473	-
SE1170	SERP1170	Biotin carboxyl carrier protein (FabE)	CP000029.1:1205593-1206033	0.59055	0.893842	0.949145	-
SE0954	SERP0954	-	CP000029.1:970496-970834	0.568507	0.894526	0.949393	-
SE0025	SERP0025	Sugar transport related protein	CP000029.1:20789-22153	0.594059	0.895851	0.949845	-
SE2208	SERP2208	alkaline phosphatase	CP000029.1:2242520-2243989	0.697151	0.895439	0.949845	-
SE1684	acpS	holo-(acyl-carrier-protein) synthase	CP000029.1:1727634-1727987	0.545175	0.897141	0.950735	-
SE0123	SERP0123	Uncharacterized protein	CP000029.1:115246-116049	0.662199	0.898279	0.951463	-
SE1729	SERP1729	Putative aldehyde dehydrogenase SAOUHSC_02363	CP000029.1:1766042-1767469	0.607915	0.900216	0.953037	-
SE0994	SERP0994	Putative acetyltransferase	CP000029.1:1011687-1012190	0.674676	0.900995	0.953384	-
SE1767	SERP1767	-	CP000029.1:1811567-1812037	0.594063	0.901624	0.953571	-
SE1471	SERP1471	conserved hypothetical protein	CP000029.1:1536645-1537202	0.667178	0.906412	0.958155	-
SE0236	pta	phosphate acetyltransferase	CP000029.1:242597-243586	0.657982	0.907199	0.958421	-
SE1485	groES	10 kDa chaperonin	CP000029.1:1553765-1554049	0.586865	0.907572	0.958421	-
SE0194	SERP0194	conserved hypothetical protein	CP000029.1:202608-203564	0.770916	0.910059	0.960567	-
SE1967	SERP1967	Uncharacterized protein	CP000029.1:1986078-1987025	0.604405	0.921605	0.972268	-
SE0603	SERP0603	conserved hypothetical protein	CP000029.1:595577-596332	0.565502	0.926049	0.976469	-
SE2354	SERP2354	S-formylglutathione hydrolase FrmB	CP000029.1:2400433-2401200	0.606657	0.927137	0.977127	-
SE0087	SERP0087	conserved hypothetical protein	CP000029.1:74338-74706	0.676519	0.928287	0.977851	-
SE0997	SERP0997	UPF0346 protein SP_0947	CP000029.1:1014346-1014570	0.661177	0.929011	0.978126	-
SE1147	dnaJ	chaperone protein dnaJ	CP000029.1:1183210-1184331	0.656694	0.932028	0.980814	-
SE1744	pdp	Pyrimidine-nucleoside phosphorylase	CP000029.1:1783289-1784590	0.651431	0.933282	0.981644	-
SE1827	rpsS	30S ribosomal protein S19	CP000029.1:1859288-1859566	0.655568	0.93469	0.982635	-
SE0607	murE	UDP-N-acetylmuramoylalanyl-D-glutamyl-2,6-diamino|	CP000029.1:600125-601609	0.604832	0.936115	0.983643	-
SE1261	pyk	pyruvate kinase	CP000029.1:1302046-1303803	0.649573	0.938499	0.985658	-
SE1408	SERP1408	-	CP000029.1:1472825-1473655	0.612302	0.939	0.985693	-
SE2009	SERP2009	Putative sugar translocase	CP000029.1:2025960-2026346	0.583804	0.940518	0.986796	-
SE0416	SERP0416	degV family protein	CP000029.1:416849-417715	0.605551	0.942924	0.987357	-
SE0734	SERP0734	Non-canonical purine NTP pyrophosphatase	CP000029.1:734395-734982	0.609493	0.942304	0.987357	-
SE1855	SERP1855	Molybdopterin biosynthesis protein moeA, putative	CP000029.1:1882695-1883954	0.653768	0.942723	0.987357	-
SE1863	SERP1863	-	CP000029.1:1889405-1890160	0.661182	0.941706	0.987357	-
SE2198	SERP2198	membrane protein, putative	CP000029.1:2230873-2232129	0.610084	0.944239	0.988244	-
SE1067	zwf-2	glucose-6-phosphate 1-dehydrogenase	CP000029.1:1111612-1113096	0.647475	0.948609	0.992326	-
SE0636	atlE	Gp58	CP000029.1:627656-631663	0.646126	0.952653	0.995077	-
SE0710	SERP0710	Glycerophosphodiester phosphodiesterase domain-conta	CP000029.1:707827-708753	0.647509	0.952615	0.995077	-
SE1766	SERP1766	drug resistance transporter, EmrB/QacA family	CP000029.1:1809858-1811297	0.646218	0.952489	0.995077	-
SE1415	SERP1415	conserved hypothetical protein	CP000029.1:1478853-1479995	0.614817	0.953731	0.995711	-
SE0900	SERP0900	Uncharacterized protein	CP000029.1:911116-911922	0.646529	0.956559	0.998169	-
SE0038	SERP0038	Stage 0 sporulation protein J	CP000029.1:35494-36336	1.330997	1	1	-
SE0043	SERP0043	prophage LambdaBa04, glycosyl hydrolase, family 25, p	CP000029.1:39203-40012	0.009428	1	1	-
SE0052	SERP0052	Phosphoglycerate mutase family protein	CP000029.1:43843-44433	0.009428	1	1	-
SE0075	SERP0075	-	CP000029.1:64144-64260	0.494483	1	1	-
SE0113	SERP0113	Uncharacterized protein	CP000029.1:101647-102177	0.816526	1	1	-
SE0119	SERP0119	lysine decarboxylase	CP000029.1:111697-113034	0.63058	1	1	-
SE0140	pth	Peptidyl-tRNA hydrolase	CP000029.1:130306-130878	0.594034	1	1	-
SE0147	SERP0147	-	CP000029.1:138376-138474	1.008849	1	1	-
SE0175	rpmG-1	ribosomal protein L33	CP000029.1:181123-181266	0.634254	1	1	-
SE0196	SERP0196	2-C-methyl-D-erythritol 4-phosphate cytidylyltransferas	CP000029.1:205369-206085	0.85711	1	1	-
SE0202	SERP0202	deoxynucleoside kinase family protein	CP000029.1:212435-213097	0.648922	0.964186	1	-
SE0243	SERP0243	rrf2 family protein	CP000029.1:250392-250814	0.641349	1	1	-
SE0252	SERP0252	conserved hypothetical protein	CP000029.1:260186-260701	0.594055	1	1	-
SE0321	sarX	-	CP000029.1:326756-327115	0.330997	1	1	-
SE0345	SERP0345	-	CP000029.1:346508-346801	1.008979	1	1	-
SE0352	SERP0352	-	CP000029.1:352059-352244	-0.99057	1	1	-
SE0355	SERP0355	ybaK/ebsC family protein	CP000029.1:354519-355025	0.816526	1	1	-
SE0361	SERP0361	CBS domain protein	CP000029.1:360673-362022	0.631955	0.998086	1	-
SE0369	SERP0369	-	CP000029.1:367730-367885	1.593812	1	1	-
SE0402	SERP0402	vibriobactin and enterobactin ABC transporter, ATP-bi	CP000029.1:403850-404629	0.657599	1	1	-
SE0412	SERP0412	conserved hypothetical protein	CP000029.1:412551-413312	0.641887	1	1	-
SE0446	eno	Enolase	CP000029.1:451418-452722	0.639515	0.97693	1	-
SE0454	SERP0454	-	CP000029.1:458978-459166	0.008849	1	1	-
SE0489	SERP0489	ABC transporter, ATP-binding protein	CP000029.1:480963-481988	0.629832	0.99121	1	-
SE0492	SERP0492	-	CP000029.1:483588-483686	0.009428	1	1	-
SE0500	sufB	FeS assembly protein SufB	CP000029.1:489683-491080	0.643821	0.96096	1	-

SE0501	SERP0501	-	CP000029.1:491410-491556	0.008849	1	1	-
SE0544	SERP0544	ISChy2, transposase	CP000029.1:527330-528343	0.709591	1	1	-
SE0592	SERP0592	-	CP000029.1:586312-586575	0.574713	1	1	-
SE0618	SERP0618	competence transcription factor, putative	CP000029.1:612111-612686	0.648926	0.97252	1	-
SE0622	SERP0622	-	CP000029.1:615966-616124	0.594091	1	1	-
SE0677	SERP0677	UPF0356 protein LMOf2365_1049	CP000029.1:673326-673544	0.423823	1	1	-
SE0702	SERP0702	-	CP000029.1:697382-697516	0.008849	1	1	-
SE0761	SERP0761	Putative secreted antigen GbpB/SagA putative peptidog	CP000029.1:759899-760369	1.008849	1	1	-
SE0791	SERP0791	conserved hypothetical protein	CP000029.1:790422-790796	0.606321	1	1	-
SE0801	ftsY	signal recognition particle-docking protein FtsY	CP000029.1:803020-804246	0.64256	0.976792	1	-
SE0875	glnR	transcriptional repressor GlnR	CP000029.1:890102-890467	0.625916	0.980717	1	-
SE0880	SERP0880	-	CP000029.1:894062-894253	0.009428	1	1	-
SE0923	SERP0923	conserved hypothetical protein	CP000029.1:935697-935993	1.008849	1	1	-
SE1001	SERP1001	degV family protein	CP000029.1:1016135-1016983	0.622906	0.960322	1	-
SE1002	folA-1	dihydrofolate reductase	CP000029.1:1016993-1017478	0.494483	1	1	-
SE1031	SERP1031	membrane protein, putative	CP000029.1:1076733-1077326	0.655466	0.964183	1	-
SE1077	SERP1077	3-methyl-2-oxobutanoate dehydrogenase, beta subunit	CP000029.1:1122218-1123201	0.626232	0.977798	1	-
SE1157	SERP1157	competence protein	CP000029.1:1195607-1196281	0.68716	1	1	-
SE1225	SERP1225	DNA repair protein RadC	CP000029.1:1258518-1259072	0.68716	1	1	-
SE1241	SERP1241	-	CP000029.1:1277261-1277872	0.594191	1	1	-
SE1262	pfkA	ATP-dependent 6-phosphofructokinase	CP000029.1:1303827-1304795	0.642971	0.969334	1	-
SE1326	ribBA	3,4-dihydroxy-2-butanone 4-phosphate synthase/GTP cy	CP000029.1:1386910-1388091	0.635221	1	1	-
SE1334	SERP1334	-	CP000029.1:1394191-1394634	0.594191	1	1	-
SE1359	SERP1359	-	CP000029.1:1416692-1416838	1.593812	1	1	-
SE1402	SERP1402	lipoprotein, putative	CP000029.1:1466560-1467654	0.633074	0.999092	1	-
SE1410	SERP1410	conserved hypothetical protein	CP000029.1:1475197-1475487	1.008936	1	1	-
SE1424	SERP1424	conserved hypothetical protein	CP000029.1:1486401-1487102	0.626299	1	1	-
SE1472	SERP1472	-	CP000029.1:1537255-1537800	0.635489	1	1	-
SE1497	scrR	sucrose operon repressor	CP000029.1:1563904-1564857	0.650651	1	1	-
SE1665	ilvD	Dihydroxy-acid dehydratase	CP000029.1:1702964-1704652	0.646532	0.983876	1	-
SE1746	SERP1746	-	CP000029.1:1785278-1785397	0.424102	1	1	-
SE1753	SERP1753	-	CP000029.1:1790675-1790824	0.746214	1	1	-
SE1755	czrA	transcriptional regulator, ArsR family	CP000029.1:1791823-1792164	0.594391	1	1	-
SE1861	modA	Molybdenum ABC transporter, periplasmic molybdate-l	CP000029.1:1887499-1888284	0.643459	1	1	-
SE1870	ureB	Urease subunit alpha	CP000029.1:1895176-1895577	0.656782	1	1	-
SE1887	sugE-2	Multidrug resistance protein YkkD	CP000029.1:1910341-1910658	0.883571	1	1	-
SE1903	SERP1903	-	CP000029.1:1925480-1925578	0.423823	1	1	-
SE1932	SERP1932	conserved hypothetical protein	CP000029.1:1949922-1950725	0.797588	1	1	-
SE1934	SERP1934	conserved hypothetical protein	CP000029.1:1951279-1951599	1.008979	1	1	-
SE1963	SERP1963	Uncharacterized protein	CP000029.1:1982520-1982921	0.641349	1	1	-
SE1996	SERP1996	Uncharacterized protein	CP000029.1:2015086-2015622	0.630602	1	1	-
SE2164	SERP2164	-	CP000029.1:2192579-2192725	1.008849	1	1	-
SE2178	SERP2178	conserved hypothetical protein	CP000029.1:2210814-2211377	0.424241	1	1	-
SE2181	SERP2181	-	CP000029.1:2214989-2215114	0.009428	1	1	-
SE2204	SERP2204	efflux ABC transporter, ATP-binding protein	CP000029.1:2239585-2240334	0.668061	1	1	-
SE2207	SERP2207	-	CP000029.1:2242043-2242222	0.424102	1	1	-
SE2211	SERP2211	-	CP000029.1:2248378-2248479	0.580226	1	1	-
SE2265	SERP2265	Organic hydroperoxide resistance protein	CP000029.1:2300398-2300826	0.617513	1	1	-
SE2276	SERP2276	Protein translocase subunit SecA	CP000029.1:2308898-2311288	0.625873	0.974379	1	-
SE2333	SERP2333	conserved hypothetical protein	CP000029.1:2369726-2371429	0.631056	0.995084	1	-
SE2341	SERP2341	-	CP000029.1:2387496-2387744	1.593812	1	1	-
SE2349	fosB	fosfomycin resistance protein	CP000029.1:2395235-2395663	0.008849	1	1	-
SE2361	SERP2361	conserved hypothetical protein	CP000029.1:2406273-2407169	0.330997	1	1	-
SE2377	SERP2377	-	CP000029.1:2424484-2424930	0.594391	1	1	-
SE2378	SERP2378	ABC transporter, ATP-binding protein	CP000029.1:2424945-2425634	0.709519	1	1	-
SE2396	bioD	dethiobiotin synthetase	CP000029.1:2453071-2453742	0.746068	1	1	-
SE2403	SERP2403	-	CP000029.1:2463875-2464216	0.330997	1	1	-
SE2466	SERP2466	-	CP000029.1:2520289-2520420	0.816526	1	1	-
SE2526	SERP2526	ISSod8 transposase, TnpA_ISSod8_1	CP000029.1:2581238-2581912	0.745841	1	1	-
SE2540	SERP2540	conserved hypothetical protein	CP000029.1:2598411-2599337	0.626053	0.973074	1	-
SE2546	SERP2546	-	CP000029.1:2605087-2605179	1.008849	1	1	-
SERP_Se5	rrfB	-	CP000029.1:155183-155297	0.709543	1	1	-
